# Psychotherapies for Generalized Anxiety Disorder in Adults: A Systematic Review and Network Meta-Analysis of Randomized Clinical Trials

**DOI:** 10.1001/jamapsychiatry.2023.3971

**Published:** 2023-10-18

**Authors:** Davide Papola, Clara Miguel, Mariacristina Mazzaglia, Pamela Franco, Federico Tedeschi, Sara A. Romero, Anushka R. Patel, Giovanni Ostuzzi, Chiara Gastaldon, Eirini Karyotaki, Mathias Harrer, Marianna Purgato, Marit Sijbrandij, Vikram Patel, Toshi A. Furukawa, Pim Cuijpers, Corrado Barbui

**Affiliations:** 1 Department of Global Health and Social Medicine, Harvard Medical School, Boston, Massachusetts; 2 WHO Collaborating Centre for Research and Training in Mental Health and Service Evaluation, University of Verona, Verona, Italy; 3 Section of Psychiatry, Department of Neuroscience, Biomedicine and Movement Sciences, University of Verona, Verona, Italy; 4 Section of Clinical Psychology, Department of Clinical, Neuro and Developmental Psychology, Vrije Universiteit Amsterdam, the Netherlands; 5 WHO Collaborating Centre for Research and Dissemination of Psychological Interventions, Amsterdam Public Health Research Institute, Amsterdam, the Netherlands; 6 Department of Psychology, Pontificia Universidad Católica de Chile, Santiago, Chile; 7 Millennium Institute for Research in Depression and Personality (MIDAP), Santiago, Chile; 8 Harvard T.H. Chan School of Public Health, Harvard University, Boston, Massachusetts; 9 Psychology & Digital Mental Health Care, Department of Health Sciences, Technical University Munich, Munich, Germany; 10 Department of Health Promotion and Human Behavior, Kyoto University Graduate School of Medicine/School of Public Health, Kyoto, Japan

## Abstract

**Importance:**

Generalized anxiety disorder (GAD) is one of the most common mental disorders in adults. Psychotherapies are among the most recommended treatments for GAD, but which should be considered as first-line treatment needs to be clarified.

**Objective:**

To use a network meta-analysis to examine the short- and long-term associations of different psychotherapies with outcomes of effectiveness and acceptability in adults with GAD.

**Data Sources:**

MEDLINE, Embase, PsycINFO, and the Cochrane Register of Controlled Trials were searched from database inception to January 1, 2023, to identify randomized clinical trials (RCTs) of psychotherapies for adults with GAD.

**Study Selection:**

RCTs comparing any type of psychotherapy against another or with a control condition for the treatment of adults (≥18 years, both sexes) with a primary diagnosis of GAD were eligible for inclusion.

**Data Extraction and Synthesis:**

This study followed Cochrane standards for extracting data and assessing data quality and used the PRISMA guideline for reporting. Risk of bias of individual studies was assessed using the second version of the Cochrane risk of bias tool, and the Confidence in Network Meta-Analysis was used to rate the certainty of evidence for meta-analytical results.

**Main Outcomes and Measures:**

Eight psychotherapies were compared against one another and with 2 control conditions. Primary outcomes were severity of GAD symptoms and acceptability of the psychotherapies. Random-effects model pairwise and network meta-analyses were conducted. For effectiveness, standardized mean differences (SMDs) were pooled, and for acceptability, relative risks with 95% CIs were calculated.

**Results:**

Data from 65 RCTs were included. Effect size estimates on data from 5048 participants (mean [SD], 70.9% [11.9%] women; mean [SD] age, 42.2 [12.5] years) suggested that third-wave cognitive behavior therapies (CBTs) (SMD, −0.76 [95% CI, −1.15 to −0.36]; certainty, moderate), CBT (SMD, −0.74 [95% CI, −1.09 to −0.38]; certainty, moderate), and relaxation therapy (SMD, −0.59 [95% CI, −1.07 to −0.11]; certainty, low) were associated with reduced GAD symptoms vs treatment as usual. Relative risks for all-cause discontinuation (indication of acceptability) signaled no differences compared with treatment as usual for all psychotherapies (eg, relative risk, 1.04 [95% CI, 0.64-1.67] for CBT vs treatment as usual). When excluding studies at high risk of bias, relaxation therapy lost its superiority over treatment as usual (SMD, −0.47; 95% CI, −1.18 to 0.23). When considering anxiety severity at 3 to 12 months after completion of the intervention, only CBT remained significantly associated with greater effectiveness than treatment as usual (SMD, −0.60; 95% CI, −0.99 to −0.21).

**Conclusions and Relevance:**

Given the evidence in this systematic review and network meta-analysis for its associations with both acute and long-term effectiveness, CBT may represent the first-line therapy of GAD. Third-wave CBTs and relaxation therapy were associated with short-term effectiveness and may also be offered.

## Key Points

**Question:**

Which psychotherapies are associated with the most effective and acceptable outcomes for adults with generalized anxiety disorder?

**Findings:**

In this systematic review and network meta-analysis of 65 studies comprising 5048 participants, cognitive behavior therapy (CBT), third-wave CBTs, and relaxation therapy outperformed treatment as usual for measures of effectiveness; after removing studies with high risk of bias, only CBT and third-wave CBTs remained superior to treatment as usual, and only CBT was associated with long-term effectiveness. Treatment as usual was not outperformed by any psychotherapy in terms of treatment acceptability.

**Meaning:**

Considering the trade-off between effectiveness and acceptability, effectiveness in the long term, and certainty in the level of evidence, CBT should be considered a first-line choice for treatment of generalized anxiety disorder.

## Teaser

This systematic review and network meta-analysis of randomized clinical trials assesses the associations of various psychotherapies with short- and long-term effectiveness and acceptability for treatment of generalized anxiety disorder among adults.

## Introduction

In recent decades, a large number of randomized clinical trials (RCTs) have been conducted to examine the effects of psychotherapies for generalized anxiety disorder. These studies have shown that psychological treatments have beneficial effects, both in terms of symptom reductions and increased well-being.^1^ So far, however, quantitative syntheses of RCTs informing psychotherapies for generalized anxiety disorder have been explored only by pairwise meta-analyses, through which it is possible to compare no more than 2 interventions at the same time. Due to the intrinsic limitations of the pairwise method, previous meta-analyses have mixed different treatments with active and inactive control conditions, generating useful but not specific results regarding the efficacy and acceptability profiles of individual types of psychotherapy.^2,3^ Which types of psychotherapy should be prioritized for generalized anxiety disorder is yet to be substantiated by a thorough and consistent investigation using a method suited for this purpose. In network meta-analysis, it is possible to rank treatment options by comparing multiple treatments using both direct comparisons of interventions within RCTs and indirect comparisons across trials.^4^ Because psychotherapy for mental health problems is dynamic and controversial,^5,6,7^ shedding light on the most appropriate psychotherapies in terms of risk to benefit ratio is a priority that aims to increase recourse to psychological interventions backed by trustworthy evidence-based science.^8^ Given this background, the present systematic review and network meta-analysis assessed the comparative effectiveness and acceptability of the different types of psychotherapy for the treatment of adults experiencing generalized anxiety disorder.

## Methods

This systematic review and network meta-analysis was reported according to the Preferred Reporting Items for Systematic Reviews and Meta-Analyses (PRISMA) guideline specific for network meta-analyses^9,10^ (eAppendix A in Supplement 1). The study protocol was published in advance in PROSPERO.

### Identification and Selection of Studies

Two independent investigators (D.P., P.F.) searched MEDLINE, Embase, PsycINFO, and the Cochrane Register of Controlled Trials from database inception to January 1, 2023, to identify RCTs examining the effects of psychotherapy for any anxiety disorder compared with any other psychotherapy or control conditions, an enterprise that we termed the *anxiety meta-analytical research domain*.^11^ From this pool of RCTs, the same 2 investigators further selected only RCTs comparing any kind of psychotherapy against another, or with a control condition, for the treatment of adults (18 years or older, both sexes) having a primary diagnosis of generalized anxiety disorder according to any standard operationalized criteria, including the Research Diagnostic Criteria, *DSM-III*, *DSM-III-R*, *DSM-IV*, *DSM-IV-TR*, *DSM-5*, *International Statistical Classification of Diseases and Related Health Problems, Tenth Revision*, or *International Classification of Diseases, 11th Revision* or that selected patients with anxiety according to a cutoff on a self-report scale of anxiety. Psychotherapies could be delivered in any type of treatment delivery format.^12^ Two independent raters (D.P., M.M.) extracted relevant data on study characteristics and outcome measures. For both screening and data extraction, disagreements were resolved by discussion and arbitration by senior review authors (P.C., C.B.). For the full search strategy, see eAppendix B in Supplement 1. We grouped therapies in 8 nodes (behavior therapy, cognitive behavior therapy [CBT], cognitive restructuring, psychoeducation, psychodynamic therapy, relaxation therapy, supportive psychotherapy, and third-wave CBTs) and controls in 2 nodes. Two independent researchers (D.P., C.M.) classified the psychotherapies, and conflicts were resolved through discussion with senior authors (C.B., T.A.F., and P.C.) Definitions of interventions and controls are given in Table 1.

**Table 1.** Definition of Interventions and Controls

Treatment	Definition
Experimental intervention	
Behavior therapy	Intervention, with or without physiological elements, aimed at either patient habituation or extinction to anxiety-provoking situations and sensations through repeated symptom induction (eg, in vivo exposure, interoceptive exposure).
CBT	Intervention, with or without psychoeducational components, containing cognitive restructuring plus behavior or relaxation therapy elements or both.
Cognitive restructuring	Intervention that aims to identify and dispute cognitive distortions, ie, irrational or maladaptive thoughts using strategies such as Socratic questioning, thought recording, and guided imagery.
Psychoeducation	Intervention in which patients are only provided information about their disorder.
Psychodynamic therapy	Focused on revealing and resolving intrapsychic or unconscious conflicts.
Relaxation therapy	Intervention using a type of physiological training (eg, progressive muscle relaxation, or applied relaxation) to reduce physiological manifestations of anxiety.
Supportive psychotherapy	Intervention with or without a psychoeducational component, intended as sessions in which patients are administered an active, although nonspecific, psychological treatment.
Third-wave CBT	Intervention including acceptance and commitment therapy, mindfulness-based therapy, and other so-called third-wave therapies administered with or without other CBT components (eg, exposure, cognitive restructuring, breathing retraining, or muscle relaxation).
Control	
Treatment as usual	Participants receive assessment only, with or without simple provision of informational material or minimal therapist contact, or both, and participants know they will not receive the active treatment after the trial. Participants in this condition are typically allowed to seek or continue treatment as available in the community; when such additive treatments are substantive, we included such trials only if there was balance between the 2 compared groups.
Waiting list	Participants receive assessment, with or without simple provision of informational material or minimal therapist contact, or both, and participants know they will receive the active treatment in question after the waiting phase.

Abbreviation: CBT, cognitive behavioral therapy.

### Risk of Bias

We assessed the risk of bias of the included studies using version 2 of the Cochrane risk of bias tool for randomized trials (ROB 2).^13^ Investigators (D.P., C.G., and M.P.) independently used the ROB 2 signaling questions to form judgments on the 5 ROB 2 domains. Disagreements were resolved by discussion and arbitration by senior review authors (P.C., C.B.).

### Outcomes

We considered 2 primary outcomes: generalized anxiety disorder symptoms at study end point (continuous outcome, indicated as effectiveness) and all-cause trial discontinuation (dichotomous outcome, indicated as acceptability). For the effectiveness outcome, we selected 1 scale for each study using a preplanned hierarchical algorithm (eAppendix C in Supplement 1), giving priority to scales specifically developed for generalized anxiety disorder. All-cause discontinuation was measured as the proportion of participants who dropped out from the end-of-treatment assessment for any reason. As a secondary outcome, we analyzed severity of anxiety symptoms at 3 to 12 months of follow-up after completion of the intervention.

### Statistical Analysis

We conducted a series of pairwise meta-analyses for all direct comparisons using a random-effects pooling model. For each outcome, we performed a frequentist network meta-analysis with a random-effects model. For the continuous outcome of effectiveness, we pooled the standardized mean differences (SMDs) using intention-to-treat data when available and completers data otherwise. A 2-sided *P* < .05 or a 95% CI excluding 0 was considered statistically significant. For the dichotomous outcome of acceptability, we calculated relative risks (RRs) with a 95% CI. A 2-sided *P* < .05 or a 95% CI excluding 1 was considered statistically significant. Dichotomous data were calculated on a strict intention-to-treat basis, considering the total number of randomly allocated participants as the denominator. Corresponding to intervention definitions (Table 1), when a study included different groups with a slightly different version of the same intervention, we pooled these groups into a single one.^14^

To assess the transitivity assumption, we compared the distribution of the percentage of women, mean age, number of psychotherapy sessions, and baseline symptoms severity across comparisons. Furthermore, we performed meta-regression analyses on the same variables, to identify possible treatment effect moderators. We considered that distribution differences in specific study characteristics across the different comparisons were only relevant in case of significant imbalances according to visual inspection of box plots generated for the purpose, the Kruskal-Wallis test, and meta-regression analyses showing an association with treatment effect.^12,15,16^ The variance in the random-effects distribution was assessed by means of τ^2^ in comparison with empirically derived evidence.^17,18^ For each comparison, we evaluated the presence of incoherence by comparing direct and indirect evidence within each closed loop and through the side-splitting approach by using the Stata commands *mvmeta*, *ifplot*, and *network sidesplit all* in the Stata network suite.^19^

For the effectiveness outcome, we conducted a series of preplanned sensitivity analyses to test the consistency of our preplanned outcome hierarchy and to examine whether the results for the primary outcome of effectiveness changed when we excluded studies that included participants without formal diagnosis of generalized anxiety disorder and studies that used *DSM-III* criteria to diagnose generalized anxiety disorder. A further set of sensitivity analyses was carried out excluding trials judged to be at high risk of bias to explore the putative associations of study quality with effectiveness and to test whether the results could be influenced by considering behavior experiments either as a cognitive or a behavioral component. If 10 or more studies were included in a direct pairwise comparison, we assessed publication bias by visually inspecting the funnel plot and testing for asymmetry with the Egger regression test.^20,21^

We assessed the certainty in the body of evidence for the primary outcomes through the Confidence in Network Meta-Analysis application.^22^ We also produced a treatment hierarchy by means of surface under the cumulative ranking curve (SUCRA) and mean ranks, having treatment as usual as the reference.

Statistical evaluations and production of network graphs and figures were performed using the network and network graph packages in Stata/SE, version 16.1 (StataCorp LLC).^23^

## Results

### Characteristics of Included Studies

**Figure 1.**
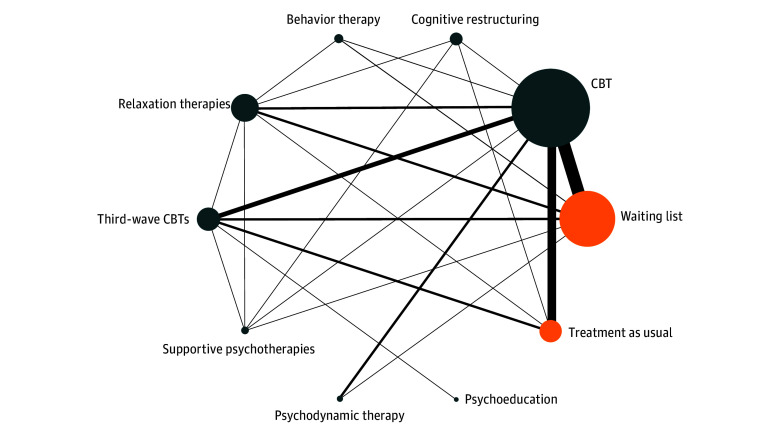
Network Plot of Evidence for Effectiveness Line thickness is proportional to the precision of each direct estimate; circle size is proportional to the number of studies including that treatment. Gray circles represent psychotherapies; orange circles, controls. CBT indicates cognitive behavior therapy.

The searches identified 19 487 records. After removing duplicates and examining titles and abstracts we selected 125 records for full-text assessment. Eventually, we selected 65 studies^24,25,26,27,28,29,30,31,32,33,34,35,36,37,38,39,40,41,42,43,44,45,46,47,48,49,50,51,52,53,54,55,56,57,58,59,60,61,62,63,64,65,66,67,68,69,70,71,72,73,74,75,76,77,78,79,80,81,82,83,84,85,86,87,88^ for inclusion in the network analysis (eAppendixes D, E, F, and G in Supplement 1). Overall, 5048 participants were randomly assigned to the 8 different psychotherapies (behavior therapy, CBT, cognitive restructuring, psychoeducation, psychodynamic therapy, relaxation therapy, supportive psychotherapy, and third-wave CBTs) and 2 different control conditions (treatment as usual and waiting list) (Figure 1). As shown in the Table 2, the mean (SD) age of the participants was 42.2 (12.5) years. The mean (SD) percentage of included women was 70.9% (11.9%) and of men was 29.1% (4.9%). The included studies were published across 42 years (1980 to 2022), following a progressive trend in terms of number of publications per decade. Studies were generally short (1-12 weeks), with follow-up observations up to 1 year after treatment completion (mean [SD], 23.6 [13.6] weeks). The mean (SD) number of therapy sessions was 11 (5) per RCT. Twenty-six studies (40%)^24,25,27,28,34,36,39,41,49,52,54,58,59,64,65,66,67,68,69,71,75,76,77,78,87,88^ used scales specifically designed to capture generalized anxiety disorder symptoms.

**Table 2.** Characteristics of Randomized Clinical Trials Included in the Network Meta-Analysis

Characteristic	Studies, No. (%)
Number of studies	65
Number of patients	5048
Sex, mean (SD), % of participants	
Men	29.1 (4.9)
Women	70.9 (11.9)
Age, mean (SD), y	42.2 (12.5)
Year of publication	
1980-1990	5 (8)
1991-2000	8 (12)
2001-2010	18 (28)
2011-2022	34 (52)
Study duration, wk	
1-12	42 (65)
13-26	21 (32)
27-36	2 (3)
Follow-up duration, wk^a^	
1-12	13 (39)
13-26	13 (39)
27-56	7 (22)
Number of sessions	
4-8	23 (35)
9-12	23 (35)
13-30	19 (30)
Risk of bias	
Low risk	11 (17)
Some concerns	31 (48)
High risk	23 (35)
Type of analysis	
Intention to treat	22 (36)
Per protocol	43 (64)
Type of outcome scale^b^	
Focused on generalized anxiety disorder	26 (40)
Focused on anxiety	37 (57)
Focused on worry	2 (3)

^a^ Follow-up data were provided by 36 studies.

^b^ For the hierarchy of outcomes, see eAppendix C in Supplement 1.

### Risk of Bias Evaluation

Twenty-three studies (35%)^27,29,32,35,37,40,42,46,47,48,49,51,57,60,61,63,65,73,74,81,83,84,87^ were at high risk of bias, 31 studies (48%)^24,25,26,28,30,34,36,38,41,43,44,45,50,52,55,56,58,59,64,66,69,70,71,72,75,77,80,82,85,86,88^ were evaluated as having “some concerns,” and 11 studies (17%)^31,33,39,53,54,62,67,68,76,78,79^ were considered at low risk of bias (Table 2, eAppendix H in Supplement 1). In the domain of “selection of the reported result,” the majority of the RCTs failed to provide information on the study protocol and the preplanned analysis plan, leading to “some concerns” judgment in 41 studies (63%)^24,26,28,29,30,32,34,35,37,38,40,42,43,44,46,47,48,49,50,51,52,55,57,61,63,64,65,66,69,71,72,73,74,75,81,82,83,84,86,87,88^; 41 studies (63%)^24,25,26,28,29,30,32,34,35,37,38,40,44,45,46,47,48,50,51,52,55,56,57,58,59,60,63,64,65,70,71,73,74,75,77,80,83,84,86,87,88^ failed to adequately report on the randomization process in the “randomization process” domain, with details on allocation concealment being almost never reported. That RCT statistical analyses were carried out mainly following a per-protocol approach (43 [64%]) had a backlash on the “deviations from the intended interventions” domain, with more than half the studies being classified as either having some concerns (21 [32%])^24,25,26,28,30,34,36,40,41,45,49,52,61,64,66,71,75,82,85,86,88^ or at high risk (19 [29%]).^27,29,32,35,37,46,47,48,51,57,60,63,65,73,74,81,83,84,87^ On the other hand, 59 RCTs (91%)^24,25,26,27,28,29,30,31,33,34,35,36,37,38,39,40,41,42,43,44,45,46,48,49,50,51,52,53,54,55,56,57,58,59,60,61,62,64,66,67,68,69,70,71,72,74,75,76,77,78,79,80,81,82,84,85,86,87,88^ had low risk of bias in the measurement of the outcome.

### Study Outcomes

**Figure 2.**
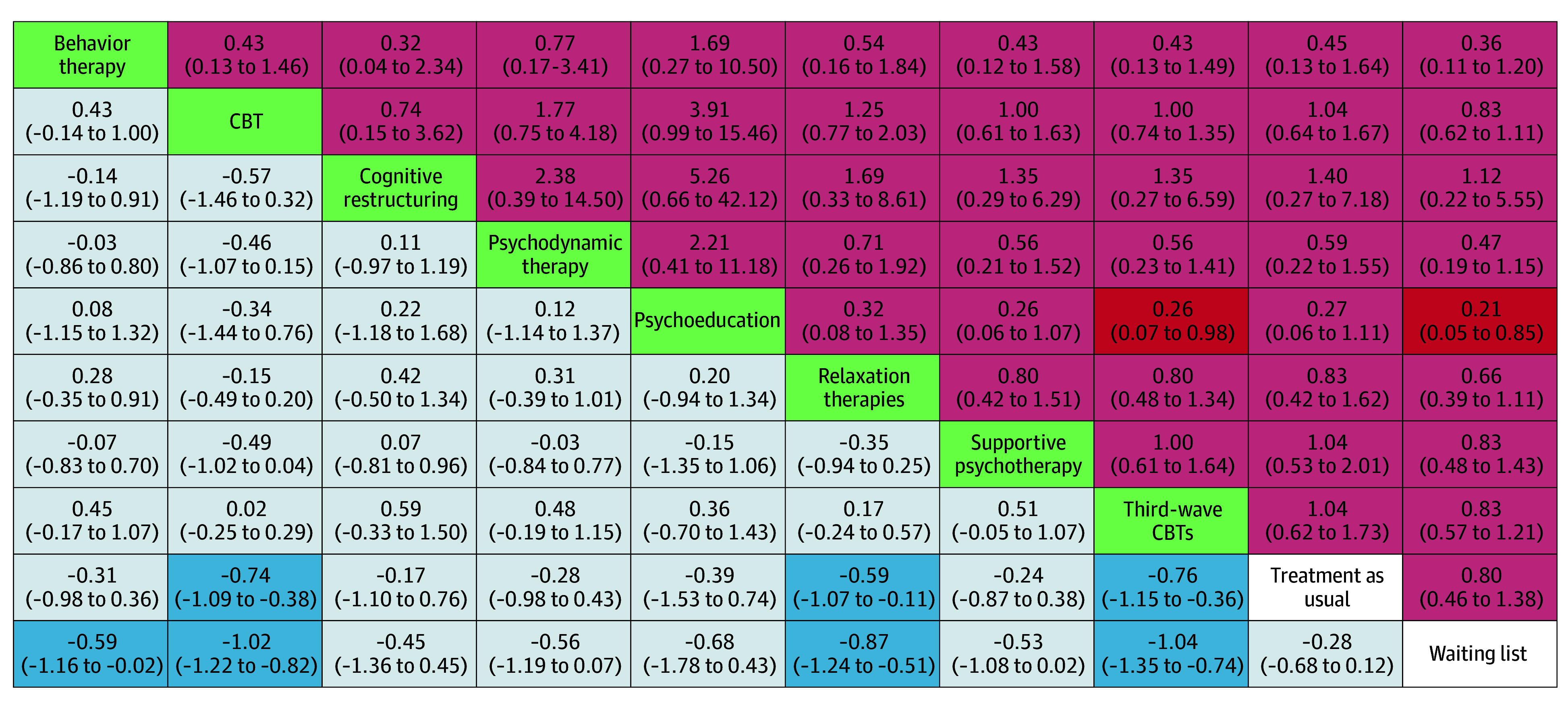
Net League Table of Head-to-Head Comparisons Standardized mean differences and 95% CIs are reported for the primary outcome of effectiveness (light blue), with standardized mean differences lower than 0 favoring the column-defining treatment. Relative risks and 95% CIs are reported for the primary outcome of acceptability (light red), with relative risk lower than 1 favoring the column-defining treatment. Green represents interventions; white (Treatment as usual, Waiting list), controls. Statistically significant results are in dark red and blue boxes. CBT indicates cognitive behavior therapy.

**Figure 3.**
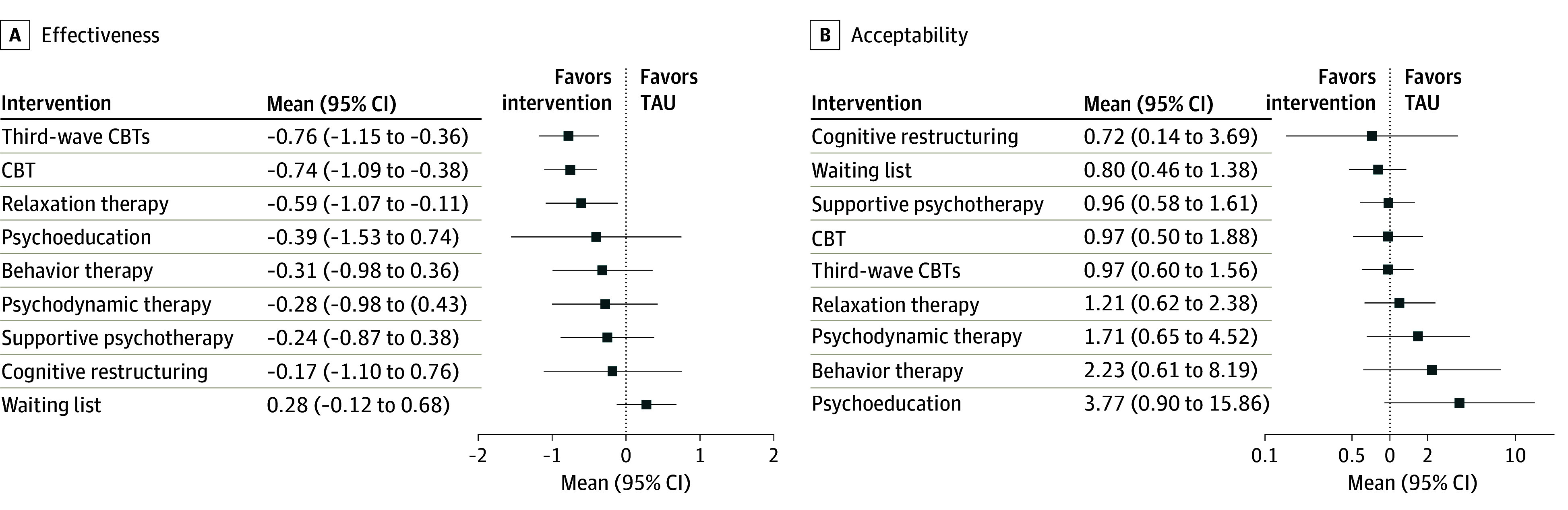
Forest Plots for Effectiveness and Acceptability, Comparing Each Psychotherapy With Treatment as Usual (TAU) Reference treatment for both plots is TAU. CBT represents cognitive behavior therapy.

Figure 1 shows the network plot for the primary effectiveness outcome. We found no evidence of violations of the transitivity assumption when assessing the distribution of effect modifiers across comparisons (eAppendix I in Supplement 1). Results for both primary outcomes (effectiveness and acceptability) are shown in Figure 2 in the form of a net league table and in Figure 3 as forest plots. For the 2 primary outcomes, all standard pairwise meta-analyses, network meta-analyses, assessments of heterogeneity and incoherence, small study effect, and quality of evidence are reported in eAppendix J and eAppendix K in Supplement 1.

Third-wave CBTs (SMD, −0.76 [95% CI, −1.15 to −0.36]; SUCRA, 87.2%; moderate certainty), CBT (SMD, −0.74 [95% CI, −1.09 to −0.38]; SUCRA, 85.1%; moderate certainty), and relaxation therapy (SMD, −0.59 [95% CI, −1.07 to −0.11]; SUCRA, 70.4%; low certainty) were superior both to treatment as usual (reference) and waiting list (SMD, 0.28 [95% CI, −0.12 to 0.68]; SUCRA, 5.1%; low certainty) in relieving the symptoms of generalized anxiety disorder. Behavior therapy was also found to be superior to waiting list (SMD, −0.59 [95% CI, −1.16 to −0.02]). No significant differences in terms of effectiveness between psychotherapies were found (Figure 2 and Figure 3). The global τ^2^ was 0.24, and there was no evidence of global inconsistency according to the design-by-treatment interaction test (χ^2^, 15.27; *P* = .91). None of 23 loops (22 triangular, 1 quadratic) showed signs of incoherence when tested through the loop-specific approach. There was no inconsistency between direct and indirect estimates, as investigated through the *sidesplit all* Stata command. Regarding the certainty in the evidence assessed through the Confidence in Network Meta-Analysis tool, we did not rate any of the comparisons as high certainty, mainly because of within-study bias and heterogeneity. Certainty in the estimate was mainly low, with selected comparisons scoring moderate or very low (mostly indirect comparisons). We identified 2 comparisons featuring more than 10 studies (waiting list vs CBT^27,32,33,35,37,38,39,40,43,44,45,48,52,54,55,57,58,60,62,66,67,68,70,74,75,78,79,80,84,88^ and third-wave CBTs vs CBT^24,25,28,31,53,62,77,80,81,83,85^); both the visual inspection of the funnel plot and the Egger test were negative for small study effects.

Apart from psychoeducation, which was slightly less accepted than third-wave CBTs and waiting list, no differences for the primary acceptability outcome were found between different psychotherapies, between psychotherapies and comparators, and between comparators (eg, RR, 1.04 [95% CI, 0.64-1.67] for CBT vs treatment as usual) (Figure 2). The acceptability network showed low heterogeneity (τ^2^ = 0.08; *P* = .10), no incoherence was found at the loop level, and the design-by-treatment interaction model indicated no incoherence in the network (χ^2^ = 16.8; *P* = .81). There was no evidence of inconsistency between all direct and indirect estimates. No comparison gained the rating of high certainty in the estimate. Most comparisons were rated at moderate or low certainty. We identified just 1 comparison featuring more than 10 studies (waiting list vs CBT^27,32,35,37,38,39,40,43,44,45,52,54,55,57,58,60,62,66,67,68,69,70,74,75,78,80,84,88^), but we detected no small study effect.

At 3 to 12 months after the completion of the study (secondary outcome: effectiveness at follow-up) (eAppendix L in Supplement 1), only CBT performed better than treatment as usual (SMD, −0.60; 95% CI, −0.99 to −0.21), and the effect sizes for third-wave CBTs and relaxation therapy became demonstrably smaller than for the acute phase. eAppendix L in Supplement 1 shows that τ^2^ decreased to 0.20, and the other tests provided no evidence of inconsistency at both the network (χ^2^ = 5.05; *P* = .93) and loop levels.

### Sensitivity Analysis

Preplanned sensitivity analyses (eAppendix M in Supplement 1) indicated the internal consistency of our outcome hierarchy, with results of the effectiveness analysis remaining overall unaltered when considering each of the 3 types of outcome scales at the top of the hierarchy and when each of these types of outcome scales was considered as unique contributors of data for the primary effectiveness outcome. Also, results remained unaltered after excluding studies^24,25,67,81^ that enrolled participants scoring above threshold on anxiety questionnaires but who had not received a formal diagnosis of generalized anxiety disorder and studies^37,40,47,57,86^ that used *DSM-III* criteria to establish the diagnosis of generalized anxiety disorder. Three additional post hoc sensitivity analyses conducted (eAppendix N in Supplement 1) showed that excluding 23 studies with high risk of bias (35%)^27,29,32,35,37,40,42,46,47,48,49,51,57,60,61,63,65,73,74,81,83,84,87^ from the network caused a decrease in the effectiveness of relaxation therapy (SMD, −0.47; 95% CI, −1.18 to 0.23), which lost its superiority over treatment as usual. The effectiveness ranking of psychotherapies did not change when CBT trial groups delivering cognitive restructuring and behavior experiments to test belief were counted as cognitive restructuring instead of CBT. Finally, the exclusion of RCTs that performed statistical analyses following a per-protocol approach led to the exclusion of two-thirds of the studies,^24,25,26,27,29,30,31,32,34,35,36,37,38,40,41,42,45,46,47,48,49,51,54,57,58,60,61,63,64,65,66,71,73,74,75,78,81,82,83,84,86,87,88^ and the network analysis became underpowered to detect differences between intervention and control conditions. eAppendix O in Supplement 1 gives the differences between the prespecified protocol and this report.

## Discussion

This systematic review and network meta-analysis found moderate to large effect sizes favoring third-wave CBTs, CBT, and relaxation therapy over treatment as usual for treatment of the acute phase of generalized anxiety disorder. We noted no effectiveness differences between different types of psychotherapies and did not detect critical differences in terms of acceptability. The latter finding suggests that any psychotherapy is as accepted as treatment as usual. Results from our secondary outcome analysis, suggesting that after a mean (SD) of 23.6 (13.6) weeks from the end of the psychotherapy only CBT remained more effective than treatment as usual, are consistent with previous findings.^3^

Although network meta-analyses are more specific than pairwise meta-analyses in disentangling and systematizing the different psychotherapy protocols, the boundaries between different kinds of psychotherapies are often blurred, and 1 stand-alone intervention can also be a component of a more structured psychotherapy. For example, a standard package of CBT for generalized anxiety disorder typically includes both cognitive restructuring and applied relaxation along with education about the nature of anxiety, training in the recognition and monitoring of situational, physiological, cognitive, and behavior cues associated with anxious responding, and imaginal exposure to anxiety cues coupled with coping skill rehearsal.^89,90,91^ We limited the overlap between therapies by isolating the 2 most important CBT components in 2 separate nodes: cognitive restructuring and relaxation therapy. While pure cognitive restructuring was not a decisively active ingredient when considered alone, relaxation therapy continues to occupy an ambiguous role in the treatment of generalized anxiety disorder.^3,92^ Relaxation therapy outperformed treatment as usual in the main analysis but could not stand its ranking position when high–risk of bias studies were removed in a sensitivity analysis or at follow-up assessment. Also, the level of certainty in the evidence for relaxation therapy in the main analysis was judged as low, mainly for concerns related to statistical heterogeneity.

While our findings support other research showing that traditional CBT itself is the leading psychotherapy for generalized anxiety disorder,^3^ as well as for other anxiety disorders,^93,94,95^ third-wave CBTs have recently emerged as solid alternatives.^96^ In recent times, there has been growing interest in testing third-wave CBTs across mood and anxiety disorders. The burst in the production of randomized evidence on third-wave CBTs was captured by our systematic review, as only 1 of the 20 RCTs assessing them was published before 2010.^69^ The third wave of CBT hit the shore 2 decades ago,^96^ leaning on a set of new behavior and cognitive approaches based on contextual concepts focused more on the persons’ relationship to thought and emotion than on their content.^97^ Our findings on the equal effectiveness associated with traditional CBT and third-wave CBTs are consistent with those from RCTs comparing such psychotherapies head-to-head and are also aligned with the results of previous meta-analyses.^98,99^

Our findings have implications for policy and practice. Clinical guidelines unanimously recommend CBT for the treatment of adults with generalized anxiety disorder.^92,100,101^ National Institute for Health and Care Excellence guidelines also recommend applied relaxation as the first-line choice.^100^ While our results largely confirm these indications, caution is needed when recommending relaxation techniques as stand-alone interventions. Relaxation techniques may be best valued when considered embedded in CBT protocols. Cognitive behavior therapy is equally more effective than treatment as usual when delivered in the individual, group, or guided self-help delivery format.^12,102^ A recent trial showed that the same CBT protocol is equally effective for generalized anxiety disorder when delivered in person or by videoconference.^103^ Policymakers should inform service organization according to a stepped care approach, in which people are first offered flexible and low-cost options (eg, guided self-help programs, videoconferencing) followed by more intensive and structured therapies (eg, in-person psychotherapy, drug therapy) in case of need. Future guidelines should also consider the mounting and solid evidence on third-wave CBTs.

To the best of our knowledge, the present study is the largest systematic review summarizing quantitative effects about the effectiveness and acceptability of psychotherapies for generalized anxiety disorder. Through the use of network meta-analysis methods, we compared all available psychotherapies, administered in any delivery format. We selected 1 outcome measure for each study using a preplanned outcome hierarchy. We tested such hierarchy in a set of prespecified sensitivity analyses, which demonstrated that findings do not change when scales on “worry symptoms” or “anxiety symptoms” were prioritized over scales on “generalized anxiety symptoms.” Also, our results demonstrated that behavior experiments to test beliefs do not tip the effectiveness balance when considered part of either cognitive restructuring or CBT protocols.

### Limitations

This study has limitations. First, the included RCTs were published over a relatively long period. This has inevitably introduced heterogeneity in terms of study design, diagnostic criteria, and follow-up periods. The overall interpretation of the findings should thus be cautious. Second, our analysis was based on aggregate-level data, and results of the present investigation are informative for prototypical patients only. Further analyses based on individual participant data are warranted to explore the influence of participant-level prognostic factors and effect modifiers on intervention outcomes. Third, one-third of the studies were judged to be at high risk of bias. The most frequent methodological shortcomings were the failure to report details of allocation concealment, lack of clarity on how trial authors handled missing participant data, and the low rates of studies that were accompanied by their prespecified analytical plans. Furthermore, two-thirds of the studies did not analyze data according to intention-to-treat principles, and this could have introduced a source of bias in favor of the experimental conditions. Fourth, since psychotherapy protocols of different therapies frequently share similar theoretical background and active components, on selected occasions it was difficult to draw straight lines between different types of psychotherapies. To maximize the reliability of our findings, 2 independent researchers classified the psychotherapies, and help was sought directly from the trial authors when needed. Component analyses are warranted to disentangle efficacy of components separately or in various combinations.^104^ Fifth, only a selection of possible outcomes was considered. While potentially interesting to investigate, information on outcomes such as functioning, quality of life, or psychotherapy adverse effects was seldom reported in the trial reports. We reasoned that pooling data on such secondary outcomes would have led to findings potentially biased by random error and of uncertain clinical meaning. Sixth, although comorbidity between generalized anxiety disorder and other mental health disorders is common, due to scant and inconsistent information in the trial reports, we were unable to test whether the presence of comorbidities at baseline was associated with the treatment outcome. Finally, the network meta-analysis approach is not free from technical and theoretical shortcomings, including risks of multiple statistical assumptions and the challenges in addressing the problems of intransitivity and inconsistency.^105^

## Conclusions

Moderate certainty in the evidence assessed in this systematic review and network meta-analysis suggests that CBT and third-wave CBTs are associated with effectiveness in the acute phase of generalized anxiety disorder. Although formally superior to treatment as usual in the main analysis, the low level of certainty in the evidence together with insights from secondary analyses call for further evidence to clarify the role of relaxation therapy when considered as a standalone intervention. In the longer term, only traditional CBT remained associated with greater effectiveness than treatment as usual; hence, CBT should be considered the first-line psychological treatment of generalized anxiety disorder. Data analyses using the component method are needed to shed light on which components are the most effective across the different psychotherapy protocols.

## Notes

Supplement 1. **eAppendix** A. PRISMA NMA checklist **eAppendix** B. Search strategy **eAppendix** C. Outcome hierarchy **eAppendix** D. PRISMA flow diagram **eAppendix** E. Characteristics of the included studies **eAppendix** F. Included studies **eAppendix** G. Excluded studies, with reasons **eAppendix** H. Risk of bias evaluation **eAppendix I.** Transitivity assessment and meta-regression **eAppendix** J. Primary outcome: efficacy **eAppendix** K. Primary outcome: acceptability **eAppendix** L. Secondary outcome: efficacy at follow-up **eAppendix** M. Sensitivity analyses (prespecified) 1 **eAppendix** N. Sensitivity analyses (post-hoc) **eAppendix** O. Differences between protocol and review

Supplement 2.
Data Sharing Statement
