# Supplementary material for: Psychotherapies for Generalized Anxiety Disorder in Adults: A Systematic Review and Network Meta-Analysis of Randomized Clinical Trials
Source: JAMA Psychiatry. 2023 Oct 18;81(3):250–9. doi: 10.1001/jamapsychiatry.2023.3971 (PMC10585589; doi:10.1001/jamapsychiatry.2023.3971)
Supplement: Supplement 1. — eAppendix A. PRISMA NMA checklist eAppendix B. Search strategy eAppendix C. Outcome hierarchy eAppendix D. PRISMA flow diagram eAppendix E. Characteristics of the included studies eAppendix F. Included studies eAppendix G. Excluded studies, with reasons eAppendix H. Risk of bias evaluation eAppendix I. Transitivity assessment and meta-regression eAppendix J. Primary outcome: efficacy eAppendix K. Primary outcome: acceptability eAppendix L. Secondary outcome: efficacy at follow-up eAppendix M. Sensitivity analyses (prespecified) 1 eAppendix N. Sensitivity analyses (post-hoc) eAppendix O. Differences between protocol and review [file jamapsychiatry-e233971-s001.pdf]

Supplementary Online Content

Papola D, Miguel C, Mazzaglia M, et al. Psychotherapies for generalized anxiety disorder in adults: a systematic review and network meta-analysis of randomized clinical trials. Published online October 18, 2023. *JAMA Psychiatry*. doi:10.1001/jamapsychiatry.2023.3971

eAppendix A. PRISMA NMA checklist
eAppendix B. Search strategy
eAppendix C. Outcome hierarchy
eAppendix D. PRISMA flow diagram
eAppendix E. Characteristics of the included studies
eAppendix F. Included studies
eAppendix G. Excluded studies, with reasons
eAppendix H. Risk of bias evaluation
eAppendix I. Transitivity assessment and meta-regression
eAppendix J. Primary outcome: efficacy
eAppendix K. Primary outcome: acceptability
eAppendix L. Secondary outcome: efficacy at follow-up
eAppendix M. Sensitivity analyses (prespecified) 1
eAppendix N. Sensitivity analyses (post-hoc)
eAppendix O. Differences between protocol and review

This supplementary material has been provided by the authors to give readers additional information about their work.

© 2023 American Medical Association. All rights reserved.

eAppendix A. PRISMA NMA checklist

Section/Topic	Item #	Checklist Item	Reported on Page #
TITLE			
Title	1	Identify the report as a systematic review incorporating a network meta-analysis (or related form of meta-analysis).	1
ABSTRACT			
Structured summary	2	Provide a structured summary including, as applicable:<br>Background: main objectives<br>Methods: data sources; study eligibility criteria, participants, and interventions; study appraisal; and synthesis methods, such as network meta-analysis.<br>Results: number of studies and participants identified; summary estimates with corresponding confidence/credible intervals; treatment rankings may also be discussed. Authors may choose to summarize pairwise comparisons against a chosen treatment included in their analyses for brevity.<br>Discussion/Conclusions: limitations; conclusions and implications of findings.<br>Other: primary source of funding; systematic review registration number with registry name.	2
INTRODUCTION			
Rationale	3	Describe the rationale for the review in the context of what is already known, including mention of why a network meta-analysis has been conducted.	4
Objectives	4	Provide an explicit statement of questions being addressed, with reference to participants, interventions, comparisons, outcomes, and study design (PICOS).	4
METHODS			
Protocol and registration	5	Indicate whether a review protocol exists and if and where it can be accessed (e.g., Web address); and, if available, provide registration information, including registration number.	4
Eligibility criteria	6	Specify study characteristics (e.g., PICOS, length of follow-up) and report characteristics (e.g., years considered, language, publication status) used as criteria for eligibility, giving rationale. Clearly describe eligible treatments included in the treatment network, and note whether any have been clustered or merged into the same node (with justification).	4,5
Information sources	7	Describe all information sources (e.g., databases with dates of coverage, contact with study authors to identify additional studies) in the search and date last searched.	4
Search	8	Present full electronic search strategy for at least one database, including any limits used, such that it could be repeated.	Appendix B
Study selection	9	State the process for selecting studies (i.e., screening, eligibility, included in systematic review, and, if applicable, included in the meta-analysis).	5
Data	10	Describe method of data extraction from reports (e.g., piloted forms,	5

© 2023 American Medical Association. All rights reserved.

collection process		independently, in duplicate) and any processes for obtaining and confirming data from investigators.	
Data items	11	List and define all variables for which data were sought (e.g., PICOS, funding sources) and any assumptions and simplifications made.	5, appendix D
Geometry of the network	S1	Describe methods used to explore the geometry of the treatment network under study and potential biases related to it. This should include how the evidence base has been graphically summarized for presentation, and what characteristics were compiled and used to describe the evidence base to readers.	6,7
Risk of bias within individual studies	12	Describe methods used for assessing risk of bias of individual studies (including specification of whether this was done at the study or outcome level), and how this information is to be used in any data synthesis.	5
Summary measures	13	State the principal summary measures (e.g., risk ratio, difference in means). Also describe the use of additional summary measures assessed, such as treatment rankings and surface under the cumulative ranking curve (SUCRA) values, as well as modified approaches used to present summary findings from meta-analyses.	5
Planned methods of analysis	14	Describe the methods of handling data and combining results of studies for each network meta-analysis. This should include, but not be limited to:<br>• Handling of multi-arm trials;<br>• Selection of variance structure;<br>• Selection of prior distributions in Bayesian analyses; and<br>• Assessment of model fit.	5,6
Assessment of Inconsistency	S2	Describe the statistical methods used to evaluate the agreement of direct and indirect evidence in the treatment network(s) studied. Describe efforts taken to address its presence when found.	6
Risk of bias across studies	15	Specify any assessment of risk of bias that may affect the cumulative evidence (e.g., publication bias, selective reporting within studies).	6
Additional analyses	16	Describe methods of additional analyses if done, indicating which were pre-specified. This may include, but not be limited to, the following:<br>• Sensitivity or subgroup analyses;<br>• Meta-regression analyses;<br>• Alternative formulations of the treatment network; and<br>• Use of alternative prior distributions for Bayesian analyses (if applicable).	6
RESULTS†			
Study selection	17	Give numbers of studies screened, assessed for eligibility, and included in the review, with reasons for exclusions at each stage, ideally with a flow diagram.	6,7
Presentation of network structure	S3	Provide a network graph of the included studies to enable visualization of the geometry of the treatment network.	6,7
Summary of network geometry	S4	Provide a brief overview of characteristics of the treatment network. This may include commentary on the abundance of trials and randomized patients for the different interventions and pairwise comparisons in the network, gaps of evidence in the treatment network, and potential biases reflected by the network structure.	6,7

© 2023 American Medical Association. All rights reserved.

Study characteristics	18	For each study, present characteristics for which data were extracted (e.g., study size, PICOS, follow-up period) and provide the citations.	6,7
Risk of bias within studies	19	Present data on risk of bias of each study and, if available, any outcome level assessment.	7
Results of individual studies	20	For all outcomes considered (benefits or harms), present, for each study: 1) simple summary data for each intervention group, and 2) effect estimates and confidence intervals. Modified approaches may be needed to deal with information from larger networks.	7,8 appendix I, appendix J
Synthesis of results	21	Present results of each meta-analysis done, including confidence/credible intervals. In larger networks, authors may focus on comparisons versus a particular comparator (e.g. placebo or standard care), with full findings presented in an appendix. League tables and forest plots may be considered to summarize pairwise comparisons. If additional summary measures were explored (such as treatment rankings), these should also be presented.	7,8 appendix I, appendix J
Exploration for inconsistency	S5	Describe results from investigations of inconsistency. This may include such information as measures of model fit to compare consistency and inconsistency models, P values from statistical tests, or summary of inconsistency estimates from different parts of the treatment network.	8
Risk of bias across studies	22	Present results of any assessment of risk of bias across studies for the evidence base being studied.	7
Results of additional analyses	23	Give results of additional analyses, if done (e.g., sensitivity or subgroup analyses, meta-regression analyses, alternative network geometries studied, alternative choice of prior distributions for Bayesian analyses, and so forth).	8,9
DISCUSSION			
Summary of evidence	24	Summarize the main findings, including the strength of evidence for each main outcome; consider their relevance to key groups (e.g., healthcare providers, users, and policy-makers).	9
Limitations	25	Discuss limitations at study and outcome level (e.g., risk of bias), and at review level (e.g., incomplete retrieval of identified research, reporting bias). Comment on the validity of the assumptions, such as transitivity and consistency. Comment on any concerns regarding network geometry (e.g., avoidance of certain comparisons).	10,11
Conclusions	26	Provide a general interpretation of the results in the context of other evidence, and implications for future research.	11
FUNDING			
Funding	27	Describe sources of funding for the systematic review and other support (e.g., supply of data); role of funders for the systematic review. This should also include information regarding whether funding has been received from manufacturers of treatments in the network and/or whether some of the authors are content experts with professional conflicts of interest that could affect use of treatments in the network.	12

© 2023 American Medical Association. All rights reserved.

eAppendix B. Search strategy

A comprehensive anxiety literature database was used as the information source of the present review and has been registered at the open science framework (https://osf.io/9xe2g/). Development of the database began with a systematic search on 25 April 2019 and it is kept update on every January by two independent researchers. A systematic search was conducted using a full range of terms related to the applicable interventions, disorders and outcomes. Similar databases exist for depression (1) treatments of suicide (2) post-traumatic stress disorder (3) and mental health problems in children and adolescents (4). Each of these includes several hundreds of randomized trials. We have recently named such living systematic reviews focusing on a specific research area as 'Meta-Analytic Research Domains' (MARDs) (5). From our anxiety MARD we selected trials on generalized anxiety disorders.

(1) Cuijpers P. Four decades of outcome research on psychotherapies for adult depression: an overview of a series of meta-analyses. Can Psychol 2017;58:7–19
(2) Hu MX, Palantza C, Setkowski K, Gilissen R, Karyotaki E, Cuijpers P, Riper H, de Beurs D, Nuij C, Christensen H, Calear A, Werner-Seidler A, Hoogendoorn A, van Balkom A, Eikelenboom M, Smit J, van Ballegooijen W. Comprehensive database and individual patient data meta-analysis of randomised controlled trials on psychotherapies reducing suicidal thoughts and behaviour: study protocol. BMJ Open. 2020 Dec 4;10(12):e037566.
(3) U.S. Department of Veterans Affairs. National center for PTSD, 2022. Available: https://www.ptsd.va.gov/ptsdrepository/index.asp.
(4) Weisz JR, Kuppens S, Ng MY, Eckshtain D, Ugueto AM, Vaughn-Coaxum R, Jensen-Doss A, Hawley KM, Krumholz Marchette LS, Chu BC, Weersing VR, Fordwood SR. What five decades of research tells us about the effects of youth psychological therapy: A multilevel meta-analysis and implications for science and practice. Am Psychol. 2017 Feb-Mar;72(2):79-117.
(5) Cuijpers P, Miguel C, Papola D, Harrer M, Karyotaki E. From living systematic reviews to meta-analytical research domains. Evid Based Ment Health. 2022 Jul 19:ebmental-2022-300509.

Last update: January 1st, 2023

PubMed

Search Strings for PubMed

Psychotherapy[MH] OR psychotherap*[All Fields] OR cbt[All Fields] OR "behavior therapies"[All Fields] OR "behavior therapy"[All Fields] OR "behavior therapeutic"[All Fields] OR "behavior therapeutical"[All Fields] OR "behavior therapeutics"[All Fields] OR "behavior therapeutist"[all Fields] OR "behavior therapeutists"[All Fields] OR "behavior treatment"[All Fields] OR "behavior treatments"[All Fields] OR "behaviors therapies"[All Fields] OR "behaviors therapy"[All Fields] OR "behaviors therapeutics"[All Fields] OR "behaviors therapeutic"[All Fields] OR "behaviors therapeutical"[All Fields] OR "behaviors therapeutist"[All Fields] OR "behaviors therapeutists"[All Fields] OR "behaviors treatment"[All Fields] OR "behaviors treatments"[All Fields] OR "behavioral therapies"[All Fields] OR "behavioral therapy"[All Fields] OR "behavioral therapeutics"[All Fields] OR "behavioral therapeutic"[All Fields] OR "behavioral therapeutical"[All Fields] OR "behavioral therapeutist"[All Fields] OR "behavioral therapeutists"[All Fields] OR "behavioral treatment"[All Fields] OR "behavioral treatments"[All Fields] OR "behaviour therapies"[All Fields] OR "behaviour therapy"[All Fields] OR "behaviour therapeutic"[All Fields] OR "behaviour therapeutical"[All Fields] OR "behaviour therapeutics"[All Fields] OR "behaviour therapeutist"[all Fields] OR "behaviour therapeutists"[All Fields] OR "behaviour treatment"[All Fields] OR "behaviour treatments"[All Fields] OR "behaviours therapies"[All Fields] OR "behaviours therapy"[All Fields] OR "behaviours therapeutics"[All Fields] OR "behaviours therapeutic"[All Fields] OR "behaviours therapeutical"[All Fields] OR "behaviours therapeutist"[All Fields] OR "behaviours therapeutists"[All Fields] OR "behaviours treatment"[All Fields] OR "behaviours treatments"[All Fields] OR "behavioural therapies"[All Fields] OR "behavioural therapy"[All Fields] OR "behavioural therapeutics"[All Fields] OR "behavioural therapeutic"[All Fields] OR "behavioural

© 2023 American Medical Association. All rights reserved.

therapeutical"[All Fields] OR "behavioural therapeutist"[All Fields] OR "behavioural therapeutists"[All Fields] OR "behavioural treatment"[All Fields] OR "behavioural treatments"[All Fields] OR "cognition therapies"[All Fields] OR "cognition therapie"[All Fields] OR "cognition therapy"[All Fields] OR "cognition therapeutical"[All Fields] OR "cognition therapeutic"[All Fields] OR "cognition therapeutics"[All Fields] OR "cognition therapeutist"[All Fields] OR "cognition therapeutists"[All Fields] OR "cognition treatment"[All Fields] OR "cognition treatments"[All Fields] OR psychodynamic[All Fields] OR Psychoanalysis[MH] OR psychoanalysis[All Fields] OR psychoanalytic*[All Fields] OR counselling[All Fields] OR counseling[All Fields] OR Counseling[MH] OR "problem-solving"[All Fields] OR mindfulness[All Fields] OR (acceptance[All Fields] AND commitment[All Fields]) OR "assertiveness training"[All Fields] OR "behavior activation"[All Fields] OR "behaviors activation"[All Fields] OR "behavioral activation"[All Fields] OR "cognitive therapies"[All Fields] OR "cognitive therapy"[All Fields] OR "cognitive therapeutic"[All Fields] OR "cognitive therapeutics"[All Fields] OR "cognitive therapeutical"[All Fields] OR "cognitive therapeutist"[All Fields] OR "cognitive therapeutists"[All Fields] OR "cognitive treatment"[All Fields] OR "cognitive treatments"[All Fields] OR "cognitive restructuring"[All Fields] OR (("compassion-focused"[All Fields] OR "compassion-focussed"[All Fields]) AND (therapy[SH] OR therapies[All Fields] OR therapy[All Fields] OR therape*[All Fields] OR therapis*[All Fields]OR Therapeutics [OR treatment*[All Fields])) OR ((therapy[SH] OR therapies[All Fields] OR therapy [All Fields] OR therape*[All Fields] OR therapis*[All Fields] OR Therapeutics[MH] OR treatment*[All Fields]) AND constructivist*[All Fields]) OR "metacognitive therapies"[All Fields] OR "metacognitive therapy"[All Fields] OR "metacognitive therapeutic"[All Fields] OR "metacognitive therapeutics"[All Fields] OR "metacognitive therapeutical"[All Fields] OR "metacognitive therapeutist"[All Fields] OR "metacognitive therapeutists"[All Fields] OR "metacognitive treatment"[All Fields] OR "metacognitive treatments"[All Fields] OR "meta-cognitive therapies"[All Fields] OR "meta-cognitive therapy"[All Fields] OR "meta-cognitive therapeutic"[All Fields] OR "meta-cognitive therapeutics"[All Fields] OR "meta-cognitive therapeutical"[All Fields] OR "meta-cognitive therapeutist"[All Fields] OR "meta-cognitive therapeutists"[All Fields] OR "meta-cognitive treatment"[All Fields] OR "meta-cognitive treatments"[All Fields] OR "solution-focused therapies"[All Fields] OR "solution-focused therapy"[All Fields] OR "solution-focused therapeutic"[All Fields] OR "solution-focused therapeutics"[All Fields] OR "solution-focused therapeutical"[All Fields] OR "solution focused therapies"[All Fields] OR "solution focused therapy"[All Fields] OR "solution focused therapeutic"[All Fields] OR "solution focused therapeutics"[All Fields] OR "solution focused therapeutical"[All Fields]OR "solution-focussed therapies"[All Fields] OR "solution-focussed therapy"[All Fields] OR "solution-focussed therapeutic"[All Fields] OR "solution-focussed therapeutics"[All Fields] OR "solution-focussed therapeutical"[All Fields]OR "solution focussed therapies"[All Fields] OR "solution focussed therapy"[All Fields] OR "solution focussed therapeutic"[All Fields] OR "solution focussed therapeutics"[All Fields] OR "solution focussed therapeutical"[All Fields] OR "self-control therapies"[All Fields] OR "self-control therapy"[All Fields] OR "self-control therapeutics"[All Fields] OR "self-control therapeutical"[All Fields] OR "self-control therapeutic"[All Fields] OR "self-control training"[All Fields] OR "self-control trainings"[All Fields] OR "self control therapies"[All Fields] OR "self control therapy"[All Fields] OR "self control therapeutics"[All Fields] OR "self control therapeutical"[All Fields] OR "self control therapeutic"[All Fields] OR "self control training"[All Fields] OR "self control trainings"[All Fields] OR exposure[All Fields] OR relaxation[All Fields] OR EMDR[All Fields] OR ("eye movement" and desensiti*[All Fields]) OR "panic management"[All Fields] OR "response prevention"[All Fields] OR ERP[All Fields]

AND

"social anxiety"[All Fields] OR shy[All Fields] OR ("shyness"[MeSH Terms] OR "shyness"[All Fields]) OR "test anxiety"[All Fields] OR gad[All Fields] OR "generalized anxiety"[All Fields] OR "generalised anxiety"[All Fields] OR worry[All Fields] OR ("panic"[MeSH Terms] OR "panic"[All Fields]) OR (agoraphobi[All Fields] OR agoraphobia[All Fields] OR agoraphobia'[All Fields] OR agoraphobia's[All Fields] OR agoraphobias[All Fields] OR agoraphobic[All Fields] OR agoraphobic's[All Fields] OR agoraphobics[All Fields] OR agoraphobics'[All Fields] OR agoraphobie[All Fields] OR agoraphobien[All Fields]) OR "anxiety disorder"[All Fields] OR "social phobia"[All Fields] OR "social anxiety disorder"[All Fields] OR Arachnophobia[All Fields] OR Ophidiophobia[All Fields] OR Acrophobia[All Fields] OR Agoraphobia[All Fields] OR Cynophobia[All Fields] OR Claustrophobia[All Fields] OR Mysophobia[All Fields] OR Aerophobia[All Fields] OR Trypophobia[All Fields] OR Carcinophobia[All Fields] OR Thanatophobia[All Fields] OR Glossophobia[All Fields] OR Monophobia[All Fields] OR Ornithophobia[All Fields] OR Alektorophobia[All Fields] OR Trypanophobia[All Fields] OR Anthropophobia[All

© 2023 American Medical Association. All rights reserved.

Fields] OR Aquaphobia[All Fields] OR Autophobia[All Fields] OR Hemophobia[All Fields] OR Xenophobia[All Fields] OR Ailurophobia[All Fields] OR Nyctophobia[All Fields] OR Phobophobia[All Fields] OR Philophobia[All Fields] OR Triskaidekaphobia[All Fields] OR Emetophobia[All Fields] OR Entomophobia[All Fields] OR Zoophobia[All Fields] OR Scelerophobia[All Fields] OR Cibophobia[All Fields] OR Tokophobia[All Fields] OR Pseudodysphagia[All Fields] OR Gerascophobia[All Fields] OR Technophobia[All Fields] OR Ergophobia[All Fields] OR Coulrophobia [All Fields] OR Photophobia[All Fields] OR Numerophobia[All Fields] OR Taphophobia

PsycINFO

DE "Psychotherapy" OR "Psychotherapy" OR "psychotherapies" OR "psychotherapeutic" OR "psychotherapeutical" OR "psychotherapeutics" OR DE "Behavior Therapy" OR DE "Cognitive Behavior Therapy" OR "CBT" OR "behavior therapies" OR "behavior therapy" OR "behavior therapeutic" OR "behavior therapeutical" OR "behavior therapeutics" OR "behavior therapeutist" OR "behavior therapeutists" OR "behavior treatment" OR "behavior treatments" OR "behaviors therapies" OR "behaviors therapy" OR "behaviors therapeutics" OR "behaviors therapeutic" OR "behaviors therapeutical" OR "behaviors therapeutist" OR "behaviors therapeutists" OR "behaviors treatment" OR "behaviors treatments" OR "behavioral therapies" OR "behavioral therapy" OR "behavioral therapeutics" OR "behavioral therapeutic" OR "behavioral therapeutical" OR "behavioral therapeutist" OR "behavioral therapeutists" OR "behavioral treatment" OR "behavioral treatments" OR "behaviour therapies" OR "behaviour therapy" OR "behaviour therapeutic" OR "behaviour therapeutical" OR "behaviour therapeutics" OR "behaviour therapeutist" OR "behaviour therapeutists" OR "behaviour treatment" OR "behaviour treatments" OR "behaviours therapies" OR "behaviours therapy" OR "behaviours therapeutics" OR "behaviours therapeutic" OR "behaviours therapeutical" OR "behaviours therapeutist" OR "behaviours therapeutists" OR "behaviours treatment" OR "behaviours treatments" OR "behavioural therapies" OR "behavioural therapy" OR "behavioural therapeutics" OR "behavioural therapeutic" OR "behavioural therapeutical" OR "behavioural therapeutist" OR "behavioural therapeutists" OR "behavioural treatment" OR "behavioural treatments" OR "cognition therapies" OR "cognition therapie" OR "cognition therapy" OR "cognition therapeutical" OR "cognition therapeutic" OR "cognition therapeutics" OR "cognition therapeutist" OR "cognition therapeutists" OR "cognition treatment" OR "cognition treatments" OR "cognitive therapies" OR "cognitive therapy" OR "cognitive therapeutic" OR "cognitive therapeutics" OR "cognitive therapeutical" OR "cognitive therapeutist" OR "cognitive therapeutists" OR "cognitive treatment" OR "cognitive treatments" OR "cognitive restructuring" OR DE "Emotion Focused Therapy" OR DE "Psychoanalysis" OR "psychoanalysis" OR "psychoanalytic" OR "psychoanalytical "OR DE "Psychodynamic Psychotherapy" OR "psychodynamic" OR DE "Psychotherapeutic Counseling" OR "counselling" OR "counseling" OR "problem-solving" OR "problem solving" OR "mindfulness" OR "acceptance and commitment" OR "assertiveness training" OR "behavior activation" OR "behaviors activation" OR "behavioral activation" OR "behaviour activation" OR "behaviours activation" OR "behavioural activation" OR "metacognitive therapies" OR "metacognitive therapy" OR "metacognitive therapeutic" OR "metacognitive therapeutics" OR "metacognitive therapeutical" OR "metacognitive therapeutist" OR "metacognitive therapeutists" OR "metacognitive treatment" OR "metacognitive treatments" OR "meta-cognitive therapies" OR "meta-cognitive therapy" OR "meta-cognitive therapeutic" OR "meta-cognitive therapeutics" OR "meta-cognitive therapeutical" OR "meta-cognitive therapeutist" OR "meta-cognitive therapeutists" OR "meta-cognitive treatment" OR "meta-cognitive treatments" OR DE "Solution Focused Therapy" OR "solution-focused therapies" OR "solution-focused therapy" OR "solution-focused therapeutic" OR "solution-focused therapeutics" OR "solution-focused therapeutical" OR "solution-focussed therapies" OR "solution-focussed therapy" OR "solution-focussed therapeutic" OR "solution-focussed therapeutics" OR "solution-focussed therapeutical" OR "solution focused therapies" OR "solution focused therapy" OR "solution focused therapeutic" OR "solution focused therapeutics" OR "solution focused therapeutical" OR "solution focussed therapies" OR "solution focussed therapy" OR "solution focussed therapeutic" OR "solution focussed therapeutics" OR "solution focussed

© 2023 American Medical Association. All rights reserved.

therapeutical" OR "self-control therapies" OR "self-control therapy" OR "self-control therapeutics" OR "self-control therapeutical" OR "self-control therapeutic" OR "self-control training" OR "self-control trainings" OR "self control therapies" OR "self control therapy" OR "self control therapeutics" OR "self control therapeutical" OR "self control therapeutic" OR "self control training" OR "self control trainings" OR "compassion-focused" OR "compassion-focussed" OR "compassion focused" OR "compassion focussed" OR "therapies" OR "therapy" OR "therapie" OR "therapist" OR "therapists" OR "therapeut" OR "treatment" OR "treatments" OR "constructivist" OR "therapies" OR "therapy" OR "therapie" OR "therapist" OR "therapists" OR "therapeut" OR "treatment" OR "treatments" OR "exposure" OR "relaxation" OR "EMDR" OR "eye movement and reprocessing" OR "panic management" OR "response prevention" OR "ERP"
AND
"social anxiety" or "shy" or "shyness" or "test anxiety" or "gad" or "generalized anxiety" or "generalised anxiety" or "worry" or "panic" or "agoraphobi*" OR "anxiety disorder" or "anxiety disorders" or "SAD" or "social phobia" or "social anxiety disorder" or "arachnophobia" or "ophidiophobia" or "acrophobia" or "agoraphobia" or "cynophobia or "claustrophobia" or "mysophobia or "aerophobia" or "trypophobia or "carcinophobia" or "thanatophobia" or "glossophobia" or "monophobia" or "ornithophobia" or "alektorophobia" or "trypanophobia" or "anthropophobia" or "aquaphobia" or "autophobia" or "hemophobia" or "xenophobia" or "ailurophobia" or "nyctophobia" or "phobophobia" or "philophobia" or "triskaidekaphobia" or "emetophobia" or "entomophobia" or "zoophobia" or "scelerophobia" or "cibophobia" or "tokophobia" or "pseudodysphagia" or "gerascophobia" or "technophobia" or "ergophobia" or "coulrophobia" or "hotophobia" or "numerophobia" or "taphophobia"

Embase

'psychotherapy'/exp OR psychotherap* OR 'psychotherapy' OR 'psychotherapy' OR ‘psychotherapies’ OR ‘psychotherapeutic’ OR ‘psychotherapeutical’ OR ‘psychotherapeutics’ OR ‘Behavior Therapy’/exp OR ‘Cognitive Behavioral Therapy’/exp OR ‘CBT’ OR ‘behavior therapies’ OR ‘behavior therapy’ OR ‘behavior therapeutic’ OR ‘behavior therapeutical’ OR ‘behavior therapeutics’ OR ‘behavior therapeutist’ OR ‘behavior therapeutists’ OR ‘behavior treatment’ OR ‘behavior treatments’ OR ‘behaviors therapies’ OR ‘behaviors therapy’ OR ‘behaviors therapeutics’ OR ‘behaviors therapeutic’ OR ‘behaviors therapeutical’ OR ‘behaviors therapeutist’ OR ‘behaviors therapeutists’ OR ‘behaviors treatment’ OR ‘behaviors treatments’ OR ‘behavioral therapies’ OR ‘behavioral therapy’ OR ‘behavioral therapeutics’ OR ‘behavioral therapeutic’ OR ‘behavioral therapeutical’ OR ‘behavioral therapeutist’ OR ‘behavioral therapeutists’ OR ‘behavioral treatment’ OR ‘behavioral treatments’ OR ‘behaviour therapies’ OR ‘behaviour therapy’ OR ‘behaviour therapeutic’ OR ‘behaviour therapeutical’ OR ‘behaviour therapeutics’ OR ‘behaviour therapeutist’ OR ‘behaviour therapeutists’ OR ‘behaviour treatment’ OR ‘behaviour treatments’ OR ‘behaviours therapies’ OR ‘behaviours therapy’ OR ‘behaviours therapeutics’ OR ‘behaviours therapeutic’ OR ‘behaviours therapeutical’ OR ‘behaviours therapeutist’ OR ‘behaviours therapeutists’ OR ‘behaviours treatment’ OR ‘behaviours treatments’ OR ‘behavioural therapies’ OR ‘behavioural therapy’ OR ‘behavioural therapeutics’ OR ‘behavioural therapeutic’ OR ‘behavioural therapeutical’ OR ‘behavioural therapeutist’ OR ‘behavioural therapeutists’ OR ‘behavioural treatment’ OR ‘behavioural treatments’ OR ‘cognition therapies’ OR ‘cognition therapie’ OR ‘cognition therapy’ OR ‘cognition therapeutical’ OR ‘cognition therapeutic’ OR ‘cognition therapeutics’ OR ‘cognition therapeutist’ OR ‘cognition therapeutists’ OR ‘cognition treatment’ OR ‘cognition treatments’ OR ‘cognitive therapies’ OR ‘cognitive therapy’ OR ‘cognitive therapeutic’ OR ‘cognitive therapeutics’ OR ‘cognitive therapeutical’ OR ‘cognitive therapeutist’ OR ‘cognitive therapeutists’ OR ‘cognitive treatment’ OR ‘cognitive treatments’ OR ‘cognitive restructuring’ OR ‘Emotion Focused Therapy’/exp OR ‘Psychoanalysis’/exp OR ‘psychoanalysis’ OR ‘psychoanalytic’ OR ‘psychoanalytical‘ OR ‘Psychodynamic Psychotherapy’/exp OR ‘psychodynamic’ OR ‘Psychotherapeutic Counseling’/exp OR ‘counselling’ OR ‘counseling’ OR ‘problem-solving’ OR ‘problem solving’ OR ‘mindfulness’ OR ‘acceptance and commitment’ OR ‘assertiveness training’ OR ‘behavior activation’ OR ‘behaviors activation’ OR ‘behavioral

© 2023 American Medical Association. All rights reserved.

activation’ OR ‘behaviour activation’ OR ‘behaviours activation’ OR ‘behavioural activation’ OR ‘metacognitive therapies’ OR ‘metacognitive therapy’ OR ‘metacognitive therapeutic’ OR ‘metacognitive therapeutics’ OR ‘metacognitive therapeutical’ OR ‘metacognitive therapeutist’ OR ‘metacognitive therapeutists’ OR ‘metacognitive treatment’ OR ‘metacognitive treatments’ OR ‘meta-cognitive therapies’ OR ‘meta-cognitive therapy’ OR ‘meta-cognitive therapeutic’ OR ‘meta-cognitive therapeutics’ OR ‘meta-cognitive therapeutical’ OR ‘meta-cognitive therapeutist’ OR ‘meta-cognitive therapeutists’ OR ‘meta-cognitive treatment’ OR ‘meta-cognitive treatments’ OR ‘Solution Focused Therapy’/exp OR ‘solution-focused therapies’ OR ‘solution-focused therapy’ OR ‘solution-focused therapeutic’ OR ‘solution-focused therapeutics’ OR ‘solution-focused therapeutical’ OR ‘solution-focussed therapies’ OR ‘solution-focussed therapy’ OR ‘solution-focussed therapeutic’ OR ‘solution-focussed therapeutics’ OR ‘solution-focussed therapeutical’ OR ‘solution focused therapies’ OR ‘solution focused therapy’ OR ‘solution focused therapeutic’ OR ‘solution focused therapeutics’ OR ‘solution focused therapeutical’ OR ‘solution focussed therapies’ OR ‘solution focussed therapy’ OR ‘solution focussed therapeutic’ OR ‘solution focussed therapeutics’ OR ‘solution focussed therapeutical’ OR ‘self-control therapies’ OR ‘self-control therapy’ OR ‘self-control therapeutics’ OR ‘self-control therapeutical’ OR ‘self-control therapeutic’ OR ‘self-control training’ OR ‘self-control trainings’ OR ‘self control therapies’ OR ‘self control therapy’ OR ‘self control therapeutics’ OR ‘self control therapeutical’ OR ‘self control therapeutic’ OR ‘self control training’ OR ‘self control trainings’ OR ‘compassion-focused’ OR ‘compassion-focussed’ OR ‘compassion focused’ OR ‘compassion focussed’ OR ‘exposure’ OR ‘relaxation’ OR ‘EMDR’ OR ‘eye movement and reprocessing’ OR ‘panic management’ OR ‘response prevention’ OR ‘ERP’
AND
'anxiety disorder'/exp OR 'anxiety disorder' OR 'gad'/exp OR 'gad' OR 'generalized anxiety disorder'/exp OR 'generalized anxiety disorder' OR 'generalised anxiety disorder'/exp OR 'generalised anxiety disorder' OR 'generalized anxiety' OR 'generalised anxiety' OR 'worry' OR 'social phobia'/exp OR 'social phobia' OR 'social anxiety disorder'/exp OR 'social anxiety disorder' OR 'social anxiety' OR 'acute stress disorder'/exp OR 'acute stress disorder' OR 'acute stress' OR 'panic'/exp OR 'panic' OR ‘agorophobia' OR ’Arachnophobia’ OR ’Ophidiophobia’ OR ’Acrophobia’ OR ’Agoraphobia’ OR ’Cynophobia’ OR ’Claustrophobia’ OR ’Mysophobia’ OR ’Aerophobia’ OR ’Trypophobia’ OR ’Carcinophobia’ OR ’Thanatophobia’ OR ’Glossophobia’ OR ’Monophobia’ OR ’Ornithophobia’ OR ’Alektorophobia’ OR ’Trypanophobia’ OR ’Anthropophobia’ OR ’Aquaphobia’ OR ’Autophobia’ OR ’Hemophobia’ OR ’Xenophobia’ OR ’Ailurophobia’ OR ’Nyctophobia’ OR ’Phobophobia’ OR ’Philophobia’ OR ’Triskaidekaphobia’ OR ’Emetophobia’ OR ’Entomophobia’ OR ’Zoophobia’ OR ’Scelerophobia’ OR ’Cibophobia’ OR ’Tokophobia’ OR ’Pseudodysphagia’ OR ’Gerascophobia’ OR ’Technophobia’ OR ’Ergophobia’ OR ’Coulrophobia‘ OR ’Photophobia’ OR ’Numerophobia’ OR ’Taphophobia’

Cochrane Library

ID	Search
#1	"social anxiety disorder":ti,ab,kw (Word variations have been searched)
#2	"GAD":ti,ab,kw (Word variations have been searched)
#3	"social anxiety":ti,ab,kw (Word variations have been searched)
#4	"test anxiety":ti,ab,kw (Word variations have been searched)
#5	"generalised anxiety disorder":ti,ab,kw (Word variations have been searched)
#6	"generalised anxiety":ti,ab,kw (Word variations have been searched)
#7	"worry":ti,ab,kw (Word variations have been searched)
#8	"panic":ti,ab,kw (Word variations have been searched)
#9	"panic disorder":ti,ab,kw (Word variations have been searched)
#10	"agoraphobia":ti,ab,kw (Word variations have been searched)
#11	"phobia":ti,ab,kw (Word variations have been searched)

© 2023 American Medical Association. All rights reserved.

#12 #1 OR #2 OR #3 OR #4 OR #5 OR #6 OR #7 OR #8 OR #9 OR #10 OR #11
#13 "psychotherapy":kw (Word variations have been searched)
#14 "CBT":ti,ab,kw (Word variations have been searched)
#15 cognitive behavi* therap*:ti,ab,kw (Word variations have been searched)
#16 psychodynamic:ti,ab,kw (Word variations have been searched)
#17 "psychoanalysis":ti,ab,kw (Word variations have been searched)
#18 psychoanalys*:ti,ab,kw (Word variations have been searched)
#19 "counseling":ti,ab,kw (Word variations have been searched)
#20 "problem solving":ti,ab,kw (Word variations have been searched)
#21 "acceptance and commitment":ti,ab,kw (Word variations have been searched)
#22 "assertiveness training":ti,ab,kw (Word variations have been searched)
#23 "behavioral activation":ti,ab,kw (Word variations have been searched)
#24 "behavioural activation":ti,ab,kw (Word variations have been searched)
#25 "mindfulness":ti,ab,kw (Word variations have been searched)
#26 "metacognitive therap*":ti,ab,kw (Word variations have been searched)
#27 "solution focused therap*":ti,ab,kw (Word variations have been searched)
#28 "self-control therap*":ti,ab,kw (Word variations have been searched)
#29 "self-control training":ti,ab,kw (Word variations have been searched)
#30 "exposure":ti,ab,kw (Word variations have been searched)
#31 "relaxation":ti,ab,kw (Word variations have been searched)
#32 "EMDR":ti,ab,kw (Word variations have been searched)
#33 "eye movement desensitization":ti,ab,kw (Word variations have been searched)
#34 "panic management":ti,ab,kw (Word variations have been searched)
#35 "response prevention":ti,ab,kw (Word variations have been searched)
#36 #13 OR #14 OR #15 OR #16 OR #17 OR #18 OR #19 OR #20 OR #21 OR #22 OR #23 #24 OR #25 OR #26 OR #27 OR #28 OR #29 OR #30 OR #31 OR #32 OR #33 OR #34 OR #35
#37 #12 AND #36 in Trials

© 2023 American Medical Association. All rights reserved.

eAppendix C. Outcome hierarchy

Hierarchy	Symptom severity rating scales	Abbreviation
1	Scales measuring generalized anxiety symptoms	
1.1	Clinician rated scales	
1.1.1	GAD clinical severity rating	
1.1.2	Other clinician rated scales specifically focused on generalized anxiety symptoms	
1.2	Patient self-reported scales	
1.2.1	General anxiety diary	
1.2.2	Generalized Anxiety Disorder Scale 7 items	GAD-7
1.2.3	Generalized Anxiety Disorder Questionnaire-IV	GAD-Q-IV
1.2.4	Generalized Anxiety Disorder Severity Scale	GADSS
1.2.5	Depression Anxiety Stress Scales-Short Form, anxiety subscale	DASS anxiety
1.2.6	Other patient self-reported scales specifically focused on generalized anxiety symptoms	
2	Scales measuring anxiety symptoms	
2.1	Clinician rated scales	
2.1.1	Hamilton anxiety rating scale	HAMA
2.1.2	Anxiety and Related Disorders Interview Schedule	ADIS
2.1.3	Clinical Interview for Depression, anxiety subscale	CID anxiety subscale
2.1.4	Generic assessor rating	
2.2	Patient self-reported scales	
2.2.1	Patient anxiety diary	
2.2.2	Beck Anxiety Inventory	BAI
2.2.3	State-Trait Anxiety Inventory-State Version	STAI-S
2.2.4	State-Trait Anxiety Inventory-Trait Version	STAI-T
2.2.5	Zung Self-rating Anxiety Scale	ZUNG
2.2.6	Symptom Checklist-90, anxiety subscale	SCL-90 anxiety
2.2.7	Other patient self-reported scales specifically focused on anxiety symptoms	

© 2023 American Medical Association. All rights reserved.

3	Scales measuring worry symptoms	
3.1	Clinician rated scales	
3.2	Patient self-reported scales	
3.2.1	Penn State Worry Questionnaire	PSWQ
3.2.2	Worry and Anxiety Questionnaire	WAQ
3.2.3	Why worry	WW
3.2.4	Worry scale	WS
3.2.5	Other patient self-reported scales specifically focused on worry symptoms	

© 2023 American Medical Association. All rights reserved.

eAppendix D. PRISMA flow diagram

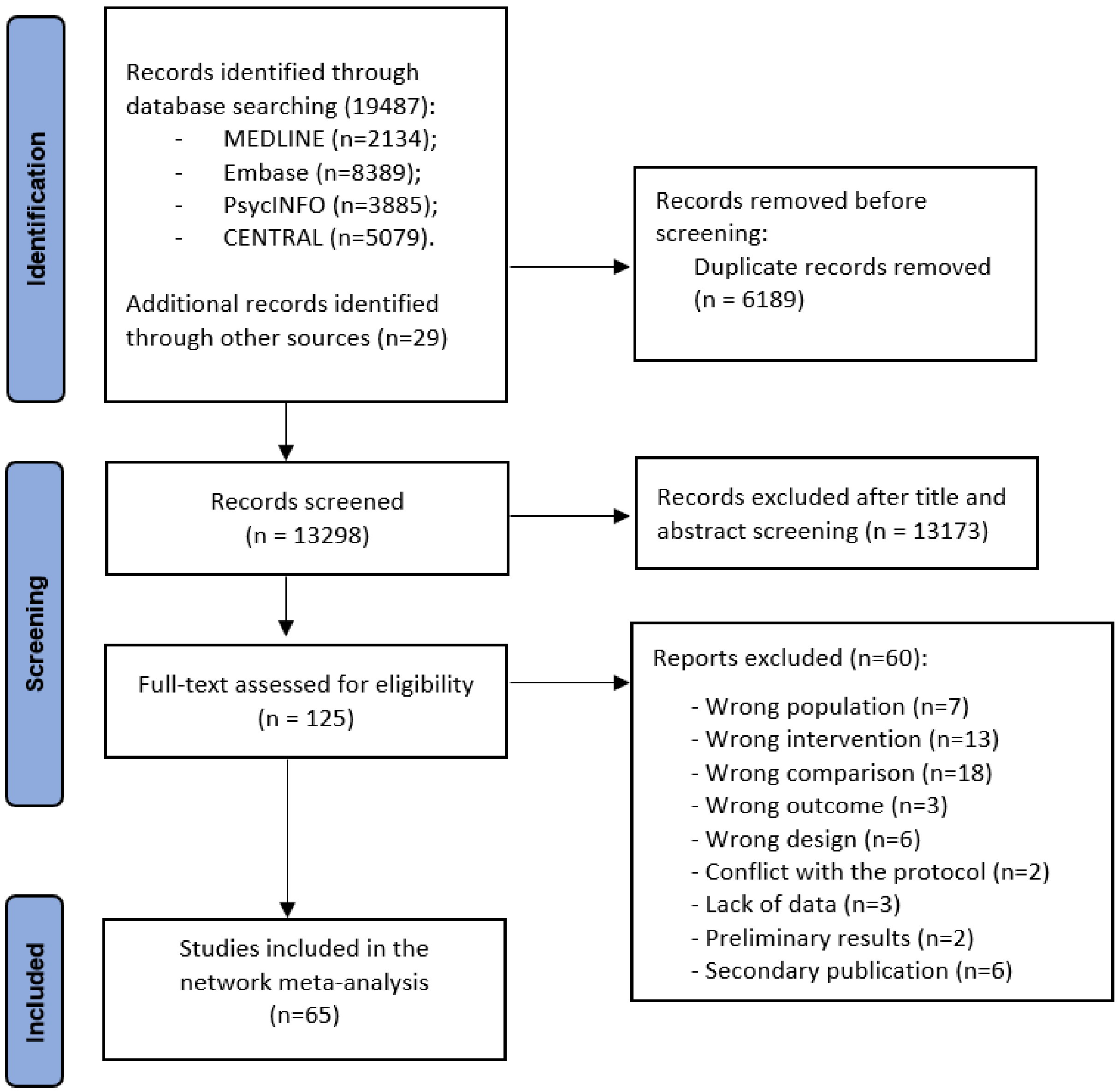

© 2023 American Medical Association. All rights reserved.

eAppendix E. Characteristics of the included studies

Unique ID	First author, year	country	Participant diagnosis	Type of recruitment	Mean age (years)	Prop % women	name of the therapy	Assigned subgroup (network node)	format	N sessions /modules	Type of comparison	Assigned subgroup (network node)	Rating scale	Self reported scale?	study end-point (weeks)	Follow-up measurements (weeks)	Analysis ITT?	ROB 2
1	Afshari, 2020[1]	Iran	Clinical diagnosis (standardized criteria not reported)	clinical	27,8	59	CBT	CBT	individual	16	-	-	GAD-7	Yes	16	12	no	Some concerns
							Dialectival behavioural therapy	3W	individual	16								
2	Afshari, 2022[2]	Iran	Clinical diagnosis (standardized criteria not reported)	clinical	27.2	65.6	CBT	CBT	individual	16	-	-	GAD-7	Yes	16	12	no	Some concerns
							Dialectival behavioural therapy	3W	individual	16								
3	Alavi, 2020[3]	Canada	DSM-IV criteria for generalized anxiety disorder	community	31,1	58,7	CBT	CBT	Guided self-help	12	TAU	TAU	BAI	Yes	12	26,52	No	Some concerns
4	Andersson, 2012[4]	Sweden	DSM-IV criteria for generalized anxiety disorder	community	40,13	77,8	Internet-based psychodynamic treatment (IPDT)	PD	Guided self-help	8	WL	WL	GAD-Q-IV	Yes	8	12, 84	no	High risk
							Internet-based CBT	CBT	Guided self-help	8								
5	Arch, 2012[5]	USA	DSM-IV criteria for a diagnosis of one or more anxiety disorders, including panic disorder with or without agoraphobia (PD/A), social anxiety disorder (SAD), specific phobia (SP), obsessive-compulsive disorder (OCD), or generalized anxiety disorder (GAD)	community	not specified for the GAD sub-sample		CBT	CBT	individual	12	-	-	ADIS-CSR	no	12	26, 52	yes	Some concerns
							Acceptance and commitment therapy	3W	Individual	12								
6	Arntz, 2003[6]	The Netherlands	DSM-III-R and DSM-IV criteria for generalized anxiety disorder	clinical	35,9	66,7	cognitive therapy	CBT	Individual	12	-	-	STAI-T	Yes	12	4, 26	No	High risk
							Applied relaxation	RT	individual	12								
7	Artiran, 2022[7]	Turkey	criteria of Generalized Anxiety Disorder (GAD) of DSM-V with	Clinical	26.7	45.2	Rational Emotive Behavior Therapy (REBT)	CR	individual	12	-	-	BAI	Yes	12	12	No	Some concerns

© 2023 American Medical Association. All rights reserved.

			comorbid mild depression				Humanistic, Client-Centered Therapy (HCCT)	SP	individual	12								
8	Avdagic, 20148	Australia	DSM-IV criteria for generalized anxiety disorder as the primary problem	community	36,2	66,7	CBT	CBT	group	6	-	-	DASS anxiety	Yes	6	12	No	Low risk
							acceptance and commitment therapy	3W	group	6								
							pharmacotherapy	Not includable in the network	-	-								
9	Barlow, 19929	USA	DSM-III-R criteria for generalized anxiety disorder	clinical	40	43,7	Progressive muscle relaxation	RT	Individual	15	WL	WL	HAMA	No	15	NR	No	High risk
							Cognitive restructuring	CBT	individual	15								
							Progressive muscle relaxation + cognitive restructuring		individual	15								
10	Berger, 201710	Europe	primary diagnosis of SAD, PDA or GDA as indicated by the MD and confirmed by the SCID	Mixed clinical and community	Not specified for the GAD sub-sample		unguided internet CBT	CBT	Unguided self-help	6	WL	WL	PSWQ	yes	9	24	yes	Low risk
11	Borkovec, 199311	USA	DSM-III-R criteria for generalized anxiety disorder	Community	37,5	65,5	CBT	CBT	Individual	12	-	-	ADIS-R	No	8	26, 52	no	Some concerns
							contrasting applied relaxation	RT	Individual	12								
							nondirective therapy	SP	individual	12								
12	Bowman, 199712	USA	DSM-III-R criteria for generalized anxiety disorder and panic disorder	community	42,9	74	self-examination therapy	CBT	Unguided self-help	-	WL	WL	HAMA	No	4	12	no	High risk
13	Brenes, 201713	USA	DSM-IV criteria for generalized anxiety disorder	Community	66,8	81,6	telephone-delivered cognitive-behavioral therapy	CBT	telephone	10	-	-	GAD-7	Yes	16	36, 60	unclear	Some concerns
							non-directive supportive therapy	SP	individual	10								
14	Butler, 198714	UK	generalised anxiety disorder (GAD) as defined by the Research Diagnostic Criteria	Clinical	37,4	64,4	Anxiety management training	CBT	Guided self-help	12	WL	WL	HAMA	No	12	26	No	High risk
15	Butler, 199115	UK	DSM-III-R criteria for generalized anxiety disorder	Clinical	35	59	Behavioural Treatment	BT	Individual	11	WL	WL	HAMA	No	12	36	No	Some concerns
							CBT	CBT	Individual	11								

© 2023 American Medical Association. All rights reserved.

16	Carl, 202016	UK	DSM-V criteria for generalized anxiety disorder	Community	30,9	68	Digital CBT	CBT	Individual	4	WL	WL	GAD-7	Yes	6	10	yes	Low risk
17	Cragan, 198417	USA	Clinical diagnosis (standardized criteria not reported)	Community	34,5	67,3	Anxiety management training	CBT	group	6	WL	WL	STAI-T	Yes	6	4	No	High risk
							Relaxation as Self-Control	RT	group	6								
18	Dahlin, 201619	Sweden	DSM-IV criteria for generalized anxiety disorder	Community	39,5	83,5	"Oroshjalpen" ("The worry help")	3W	Guided self-help	7	WL	WL	GAD-7	Yes	9	NR	No	Some concerns
19	de Almeida Sampaio, 202020	Brazil	DSM-IV criteria for generalized anxiety disorder	Community	36,5	74	Acceptance-based behavior therapy	3W	group	10	-	-	HAMA	No	14	26	no	High risk
							non-directive support therapy	SP	group	10								
20	Dugas, 201021	Canada	DSM-IV criteria for generalized anxiety disorder	clinical	38,5	66,15	CBT	CBT	Individual	12	WL	WL	ADIS-IV	No	12	NR	yes	Some concerns
							applied relaxation	RT	individual	12								
21	Dugas, 200322	Canada	DSM-IV criteria for generalized anxiety disorder	community	41,2	71,2	CBT-IU	CBT	group	14	WL	WL	ADIS-IV	No	14	NR	yes	Some concerns
22	Dugas, 202223	Canada	DSM-IV criteria for generalized anxiety disorder	community	34.6	85	Behavioral Experiments for Intolerance of Uncertainty	CBT	Individual	12	WL	WL	ADIS-CSR	No	12	26, 52	No	Some concerns
23	Durham, 199424	UK	DSM-III-R criteria for generalized anxiety disorder	Clinical	39	67	CT	CBT	Individual	14	-	-	HAMA	yes	26	26	No	high risk
							Anxiety Management Training	Not includable in the network	Individual	14								
							Analytic Psychotherapy	PD	individual	14								
24	Durham, 198725	UK	Clinical diagnosis (standardized criteria not reported)	Clinical	36,8	66	Cognitive Therapy	CBT	individual	16	-	-	Zung rater	no	16	26	no	High risk
							Behaviour Therapy	BT	individual	16								
25	Erickson, 200726	Canada	DSM IV Diagnosis of either panic disorder with or without agoraphobia, OCD, social phobia, generalised anxiety disorder, specific phobia or PTSD.	clinical	Not specified for the GAD sub-sample		CBT	CBT	group	11	WL	WL	BAI	yes	11	NR	no	High risk
26		USA		Clinical	32,9	65,4	Acceptance Based Behavior Therapy	3W	individual	16	-	-	ADIS-CSR	no	16	26	No	High risk

© 2023 American Medical Association. All rights reserved.

	Hayes-Skelton, 201327		DSM-IV criteria for generalized anxiety disorder				Applied Relaxation	RT	individual	16								
27	Hoge, 201328	USA	DSM-IV criteria for generalized anxiety disorder	Community	39	51	Mindfulness-Based Stress Reduction	3W	group	8	-	-	SIGH-A	no	8	NR	Yes	Some concerns
							Stress Management Education	PE	group	8								
28	Hoyer, 200929	Germany	DSM-IV criteria for generalized anxiety disorder	Clinical	45,4	71	Worry exposure	BT	Individual	15	WL	WL	HAMA	no	15	NR	No	High risk
							applied relaxation	RT	individual	15								
29	Hui, 201730	China	DSM-V criteria for generalized anxiety disorder	Community	65,7	42,8	CBT-IU	CBT	group	12	WL	WL	GAD-Q-IV	Yes	12	26	No	Some concerns
30	Jiang, 202231	China	DSM-V criteria for generalized anxiety disorder	clinical	36	59.45	Mindfulness-based cognitive therapy (MBCT)	3W	group	8	-	-	HAMA	no	8	12	Yes	Low risk
							CBT	CBT	group	8								
31	Jones, 201632	Canada	DSM-IV-TR criteria for generalized anxiety disorder	Community	65,1	86	Internet delivered CBT	CBT	Guided self-help	7	WL	WL	GAD-7	yes	10	NR	Yes	Low risk
32	Ladouceur, 200033	Canada	DSM-IV criteria for generalized anxiety disorder	Community	39,7	76,9	CBT	CBT	individual	16	WL	WL	ADIS-IV	no	16	NR	Yes	Some concerns
33	Leichsenring, 200934	Germany	DSM-IV criteria for generalized anxiety disorder	Community	42,5	80,7	short-term psychodynamic psychotherapy	PD	individual	30	-	-	HAMA	no	30	56	Yes	Some concerns
							CBT	CBT	Individual	30								
34	Lindsay, 198735	Scotland	Composite clinical criteria. Among them scoring at least 4 at the GHQ	Clinical	36,1	60	CBT	CBT	Individual	8	WL	WL	Zung	yes	4	NR	no	High risk
							anxiety management training	RT	individual	8								
							BDZ	Not includable in the network	/	/								
35	Lorian, 201236	Australia	DSM-IV -TR criteria for generalized anxiety disorder	Community	44,2	72,7	Internet delivered CBT	CBT	Guided self-help	6	WL	WL	GAD-7	yes	9	NR	Yes	Some concerns
36	Mennin, 201837	USA	DSM-IV criteria for generalized anxiety disorder	Community	39	75	Emotion regulation therapy	3W	individual	20	modified attention control condition	WL	ADIS-CSR	no	20	NR	yes	Some concerns

© 2023 American Medical Association. All rights reserved.

37	Mohlman, 200338	USA	DSM-IV criteria for generalized anxiety disorder	Community	66,8	65,4	CBT	CBT	individual	13	WL	WL	BAI	yes	13	NR	No	High risk
							Enhanced CBT		individual	13								
38	Newman, 202039	USA	DSM-V criteria for generalized anxiety disorder	Community	21,4	87	Mobile Program Guided Self-Help Intervention	CBT	Guided self help	8	NT	TAU	STAI-T	yes	12	38	No	High risk
39	Nordahl, 201840	Norway	DSM-IV criteria for generalized anxiety disorder	Clinical	37,7	73	CBT	CBT	individual	12	WL	WL	BAI	yes	12	NR	Yes	Low risk
							metacognitive therapy	3W	Individual	12								
40	Orvati Aziz, 202041	Iran	DSM-V criteria for generalized anxiety disorder as primary diagnosis	Other	24,7	83,3	Integrative therapy (CBT + psychodynamic therapy)	Not includable in the network	individual	15	NT	TAU	HAMA	no	15	NR	Unclear	High risk
							CBT	CBT	individual	15								
41	Ost, 200042	Sweden	DSM-III -R criteria for generalized anxiety disorder	Community	31,8	72,2	Applied relaxation	RT	individual	12	-	-	ADIS-CSR	no	12	52	No	Some concerns
							CT	CBT	individual	12								
42	Pallavicini, 200943	Italy	DSM-IV-TR criteria for generalized anxiety disorder	Community	47	75	VR and Mobile group (VRM) without biofeedback	BT	individual	8	WL	WL	GAD-7	no	8	NR	No	High risk
							VR and Mobile group (VRMB) including biofeedback		individual	8								
43	Paxling, 201144	Sweden	DSM-IV criteria for generalized anxiety disorder	Community	39,3	79,8	guided Internet-delivered CBT	CBT	Guided self-help	8	WL	WL	GAD-Q-IV	yes	8	NR	Unclear	Some concerns
44	Richards, 201645	Ireland	Self-reported GAD symptoms were confirmed to reach an acceptable clinical threshold, defined for the study as a score of 10 or above on theGAD-7measure.	Community	23,8	77,4	iCBT + clinical support	CBT	Guided self-help	7	WL	WL	GAD-7	yes	7	NR	Yes	Low risk
45	Robinson, 201046	Australia	Clinical diagnosis according to a structured diagnostic interviewMINI Version V (DSM IV)	Community	47	68,3	gSH technician-assisted	CBT	Unguided self-help	6	WL	WL	GAD-7	yes	10	NR	Yes	Low risk
							gSH clinician-assisted		Guided self-help	6								
46	Roemer, 200847	USA	DSM-IV criteria for generalized anxiety disorder	Clinical	33,6	71	acceptance-based behavioral therapy	3W	individual	16	WL	WL	ADIS - CSR	no	16	NR	Yes	Some concerns

© 2023 American Medical Association. All rights reserved.

47	Rogiers, 202148	Belgium	Clinical diagnosis according to a structured diagnostic interviewMINI Version V (DSM IV) for MDD and\or GAD	Community	42,6	66,2	'Drop it' for Repetitive negative thinking (RNT)	CBT*	Group	8	WL	WL	STAI-S	yes	12	NR	Yes	Some concerns
48	Roy, 202149	USA	Clinical diagnosis according to a structured diagnostic interviewMINI	Community	42	91	TAU + app-delivered Mindfulness training	3W	Unguided self-help	30	TAU	TAU	GAD-7	yes	8	NR	No	Some concerns
49	Roy-Byrne, 201050	USA	DSM-IV criteria for 1 or more of PD, GAD, SAD, or PTSD	clinical	Not specified for the GAD sub-sample		Coordinated Anxiety Learning and Management (CALM)	CBT	individual	8	TAU	TAU	BSI-12	no	24	48,72	yes	Low risk
50	Stanley, 199651	USA	DSM-III-R criteria for generalized anxiety disorder	Community	68,3	70,8	CBT<br>Supportive approaches	CBT<br>SP	group<br>group	14<br>14	-	-	HAMA	no	14	26	No	High risk
51	Stanley, 200352	USA	DSM-IV criteria for generalized anxiety disorder	Community	66,2	75	CBT<br>Minimal contact control	CBT<br>WL	group<br>group	15<br>15	-	-	HAMA	no	15	NR	No	High risk
52	Stanley, 201653	USA	DSM-IV criteria for generalized anxiety disorder	Community	62,9	95	Calmer Life<br>Enhanced Community Care	CBT<br>WL	Individual / telephone<br>individual	12<br>12	-	-	GAD-7	yes	12	NR	no	Some concerns
53	Stanley, 200954	USA	DSM-IV criteria for generalized anxiety disorder	Community	66,9	78,4	CBT	CBT	individual	10	TAU	TAU	GADSS	no	12	26, 39, 52, 72	yes	Low risk
54	Timulak, 202255	Ireland	DSM-V criteria for generalized anxiety disorder	Clinical	35	84,5	Emotion-Focused Therapy for GAD<br>CBT	3W<br>CBT	individual<br>individual	16<br>16	-	-	GAD-7	yes	16	26	yes	Some concerns
55	Titov, 200956	Australia	Clinical diagnosis of GAD according to a structured diagnostic interviewMINI Version V (DSM IV)	Community	44	76	Internet based clinician-assisted computerized CBT	CBT	Guided self-help	6	WL	WL	GAD-7	yes	6	NR	yes	Low risk
56	Titov, 201057	Australia	DSM-IV diagnostic criteria for GAD, social phobia, and/or panic disorder.	community	Not specified for the GAD sub-sample		iCBT - the Anxiety program	CBT	Guided self-help	6	WL	WL	PSWQ	yes	9	12	yes	Low risk
57	Van der Heiden, 201258	The Netherlands	DSM-IV-TR criteria for generalized anxiety disorder	Clinical	35	73	Meta cognitive therapy<br>Intolerance-of-uncertainty therapy	3W<br>CBT	individual<br>individual	14<br>14	WL	WL	STAI-T	Yes	14	26	Yes	Some concerns
58	Vera, 202159	Puerto RIco	Scores of at least 5.7 on the Generalized	Clinical	41	87	CBT	CBT	Individual	15	TAU	TAU		yes	28	NR	No	High risk

© 2023 American Medical Association. All rights reserved.

			Anxiety Disorder Questionnaire (GAD-Q-IV) and 56 on the Penn State Worry Questionnaire (PSWQ)				Acceptance-based behavior therapy	3W	individual	16			DASS - ANX					
59	Wells, 202060	UK	DSM-IV-TR criteria for generalized anxiety disorder	Clinical	49	60	Metacognitive Therapy	3W	individual	12	-	-	BAI	yes	12	26, 52	No	Some concerns
							Applied relaxation	RT	individual	12								
60	Wetherell, 201161	USA	DSM-IV criteria for generalized anxiety disorder	Clinical	70,8	47,5	CBT	CBT	individual	12	-	-	HAMA	No	12	26	No	High risk
							Acceptance and Commitment Therapy	3W	individual	12								
61	Wetherell, 200362	USA	DSM-IV criteria for generalized anxiety disorder	clinical	67,1	80	CBT	CBT	group	12	WL	WL	HAMA	no	12	26	No	High risk
							Discussion group	SP	group	12								
62	Wong, 201663	Hong Kong	DSM-IV criteria for generalized anxiety disorder	Clinical	50	79	mindfulness-based cognitive therapy	3W	Group	8	TAU	TAU	BAI	yes	8	12, 26, 36	Yes	Some concerns
							psychoeducation	CBT	group	8								
63	Woodward, 198064	UK	Clinical diagnosis (standardized criteria not reported)	Clinical	40,3	59,2	Cognitive restructuring	CR	Group	8	TAU	TAU	Zung	yes	8	4	No	Some concerns
							Modified systematic desensitization	RT	Group	8								
							Combined cognitive behaviour modification	CBT	group	8								
64	Zargar, 201265	Iran	DSM-IV criteria for generalized anxiety disorder	Clinical	33	100	Acceptance-based behavior therapy	3W	Individual	12	TAU	TAU	GAD-7	yes	12	NR	No	High risk
65	Zinbarg, 200766	USA	DSM-IV criteria for generalized anxiety disorder	community	41,9	63,1	CBT	CBT	individual	12	WL	WL	ADIS-CSR	no	16	NR	No	Some concerns

AD: antidepressant; ADIS-R: Anxiety Disorders Interview Schedule—Revised BAI: Beck anxiety inventory; BT: Behaviour therapy; BZP: benzodiazepine; CBT: Cognitive Behavioural Therapy; CR: cognitive restructuring; DASS: Depression Anxiety Stress Scales; DSM: Diagnostic and Statistical Manual of Mental Disorders; GAD: generalised anxiety disorder; GAD-7: General Anxiety Disorder-7 items; GAD-Q-IV: fourth edition of the Generalized Anxiety Disorder Questionnaire; GADSS: Generalized Anxiety Disorder Severity Scale; HAMA: Hamilton Rating Scale for Anxiety; ICD: International Classification of Diseases; NT: no treatment; PD: Psychodynamic therapy; PE: Psychoeducation; PSWQ: Penn State Worry Questionnaire; RT: relaxation therapies; SIGH-A: Structured Interview Guide for the Hamilton Anxiety Scale; SSRI: serotonin selective reuptake inhibitor; SP: Supportive psychotherapy; STAI: State-Trait Anxiety Inventory; TAU: Treatment as usual; UK: United Kingdom; USA: United States of America. VRET: virtual reality exposure therapy; WL: waiting list; 3W: Third-wave CBT.

© 2023 American Medical Association. All rights reserved.

eAppendix F. Included studies

1. Afshari B, Hasani J. Study of Dialectical Behavior Therapy Versus Cognitive Behavior Therapy on Emotion Regulation and Mindfulness in Patients with Generalized Anxiety Disorder. Journal of Contemporary Psychotherapy 2020; 50(4): 305-12.
2. Afshari B, Jafarian Dehkordi F, Asgharnejad Farid AA, et al. Study of the effects of cognitive behavioral therapy versus dialectical behavior therapy on executive function and reduction of symptoms in generalized anxiety disorder. Trends in psychiatry and psychotherapy 2022; 44: e20200156.
3. Alavi N, Hirji A. The Efficacy of PowerPoint-based CBT Delivered Through Email: Breaking the Barriers to Treatment for Generalized Anxiety Disorder. Journal of psychiatric practice 2020; 26(2): 89-100.
4. Andersson G, Paxling B, Roch-Norlund P, et al. Internet-based psychodynamic versus cognitive behavioral guided self-help for generalized anxiety disorder: a randomized controlled trial. Psychotherapy and psychosomatics 2012; 81(6): 344-55.
5. Arch JJ, Eifert GH, Davies C, Plumb Vilardaga JC, Rose RD, Craske MG. Randomized clinical trial of cognitive behavioral therapy (CBT) versus acceptance and commitment therapy (ACT) for mixed anxiety disorders. Journal of consulting and clinical psychology 2012; 80(5): 750-65.
6. Arntz A. Cognitive therapy versus applied relaxation as treatment of generalized anxiety disorder. Behaviour research and therapy 2003; 41(6): 633-46.
7. Artiran M, DiGiuseppe R. Rational emotive behavior therapy compared to client-centered therapy for outpatients: A randomized clinical trial with a three months follow up. Journal of Rational-Emotive & Cognitive-Behavior Therapy 2022; 40(2): 206-33.
8. Avdagic E, Morrissey SA, Boschen MJ. A randomised controlled trial of acceptance and commitment therapy and cognitive-behaviour therapy for generalised anxiety disorder. Behaviour Change 2014; 31(2): 110-30.
9. Barlow DH, Rapee RM, Brown TA. Behavioral treatment of generalized anxiety disorder. Behavior Therapy 1992; 23(4): 551-70.
10. Berger T, Urech A, Krieger T, et al. Effects of a transdiagnostic unguided Internet intervention ('velibra') for anxiety disorders in primary care: results of a randomized controlled trial. Psychological medicine 2017; 47(1): 67-80.
11. Borkovec TD, Costello E. Efficacy of applied relaxation and cognitive-behavioral therapy in the treatment of generalized anxiety disorder. Journal of consulting and clinical psychology 1993; 61(4): 611-9.
12. Bowman D, Scogin F, Floyd M, Patton E, Gist L. Efficacy of self-examination therapy in the treatment of generalized anxiety disorder. Journal of Counseling Psychology 1997; 44: 267-73.

© 2023 American Medical Association. All rights reserved.

13. Brenes GA, Danhauer SC, Lyles MF, Anderson A, Miller ME. Long-Term Effects of Telephone-Delivered Psychotherapy for Late-Life GAD. The American journal of geriatric psychiatry : official journal of the American Association for Geriatric Psychiatry 2017; 25(11): 1249-57.

14. Butler G, Cullington A, Hibbert G, Klimes I, Gelder M. Anxiety management for persistent generalised anxiety. The British journal of psychiatry : the journal of mental science 1987; 151: 535-42.

15. Butler G, Fennell M, Robson P, Gelder M. Comparison of behavior therapy and cognitive behavior therapy in the treatment of generalized anxiety disorder. Journal of consulting and clinical psychology 1991; 59(1): 167-75.

16. Carl JR, Miller CB, Henry AL, et al. Efficacy of digital cognitive behavioral therapy for moderate-to-severe symptoms of generalized anxiety disorder: A randomized controlled trial. Depression and Anxiety 2020.

17. Cragan MK, Deffenbacher JL. Anxiety management training and relaxation as self-control in the treatment of generalized anxiety in medical outpatients. Journal of counseling psychology 1984; 31(2): 123-31.

18. Dahlin M, Andersson G, Magnusson K, et al. Internet-delivered acceptance-based behaviour therapy for generalized anxiety disorder: A randomized controlled trial. Behaviour research and therapy 2016; 77: 86-95.

19. de Almeida Sampaio TP, Jorge RC, Martins DS, et al. Efficacy of an acceptance-based group behavioral therapy for generalized anxiety disorder. Depression and Anxiety 2020; 37(12): 1179-93.

20. Dugas MJ, Brillon P, Savard P, et al. A randomized clinical trial of cognitive-behavioral therapy and applied relaxation for adults with generalized anxiety disorder. Behavior therapy 2010; 41(1): 46-58.

21. Dugas MJ, Ladouceur R, Leger E, et al. Group cognitive-behavioral therapy for generalized anxiety disorder: treatment outcome and long-term follow-up. Journal of consulting and clinical psychology 2003; 71(4): 821-5.

22. Dugas MJ, Sexton KA, Hebert EA, Bouchard S, Gouin J-P, Shafran R. Behavioral experiments for intolerance of uncertainty: A randomized clinical trial for adults with generalized anxiety disorder. Behavior Therapy 2022; 53(6): 1147-60.

23. Durham RC, Murphy T, Allan T, Richard K, Treliving LR, Fenton GW. Cognitive therapy, analytic psychotherapy and anxiety management training for generalised anxiety disorder. The British journal of psychiatry : the journal of mental science 1994; 165(3): 315-23.

24. Durham RC, Turvey AA. Cognitive therapy vs behaviour therapy in the treatment of chronic general anxiety. Behaviour research and therapy 1987; 25(3): 229-34.

25. Erickson DH, Janeck AS, Tallman K. A cognitive-behavioral group for patients with various anxiety disorders. Psychiatric services (Washington, DC) 2007; 58(9): 1205-11.

© 2023 American Medical Association. All rights reserved.

26. Hayes-Skelton SA, Roemer L, Orsillo SM. A randomized clinical trial comparing an acceptance-based behavior therapy to applied relaxation for generalized anxiety disorder. Journal of consulting and clinical psychology 2013; 81(5): 761-73.

27. Hoge EA, Bui E, Marques L, et al. Randomized controlled trial of mindfulness meditation for generalized anxiety disorder: effects on anxiety and stress reactivity. The Journal of clinical psychiatry 2013; 74(8): 786-92.

28. Hoyer J, Beesdo K, Gloster AT, Runge J, Hofler M, Becker ES. Worry exposure versus applied relaxation in the treatment of generalized anxiety disorder. Psychotherapy and psychosomatics 2009; 78(2): 106-15.

29. Hui C, Zhihui Y. Group cognitive behavioral therapy targeting intolerance of uncertainty: a randomized trial for older Chinese adults with generalized anxiety disorder. Aging & mental health 2017; 21(12): 1294-302.

30. Jiang SS, Liu XH, Han N, et al. Effects of group mindfulness-based cognitive therapy and group cognitive behavioural therapy on symptomatic generalized anxiety disorder: a randomized controlled noninferiority trial. BMC psychiatry 2022; 22(1).

31. Jones SL, Hadjistavropoulos HD, Soucy JN. A randomized controlled trial of guided internet-delivered cognitive behaviour therapy for older adults with generalized anxiety. Journal of anxiety disorders 2016; 37: 1-9.

32. Ladouceur R, Dugas MJ, Freeston MH, Leger E, Gagnon F, Thibodeau N. Efficacy of a cognitive-behavioral treatment for generalized anxiety disorder: evaluation in a controlled clinical trial. Journal of consulting and clinical psychology 2000; 68(6): 957-64.

33. Leichsenring F, Salzer S, Jaeger U, et al. Short-term psychodynamic psychotherapy and cognitive-behavioral therapy in generalized anxiety disorder: a randomized, controlled trial. The American journal of psychiatry 2009; 166(8): 875-81.

34. Lindsay WR, Gamsu CV, McLaughlin E, Hood EM, Espie CA. A controlled trial of treatments for generalized anxiety. British journal of clinical psychology 1987; 26 (Pt 1): 3-15.

35. Lorian CN, Titov N, Grisham JR. Changes in risk-taking over the course of an internet-delivered cognitive behavioral therapy treatment for generalized anxiety disorder. Journal of anxiety disorders 2012; 26(1): 140-9.

36. Mennin DS, Fresco DM, O'Toole MS, Heimberg RG. A randomized controlled trial of emotion regulation therapy for generalized anxiety disorder with and without co-occurring depression. Journal of Consulting and Clinical Psychology 2018; 86(3): 268-81.

37. Mohlman J, Gorenstein EE, Kleber M, De Jesus M, Gorman JM, Papp LA. Standard and enhanced cognitive-behavior therapy for late-life generalized anxiety disorder: Two pilot investigations. American Journal of Geriatric Psychiatry 2003; 11(1): 24-32.

© 2023 American Medical Association. All rights reserved.

38. Newman MG, Jacobson NC, Rackoff GN, Bell MJ, Taylor CB. A randomized controlled trial of a smartphone-based application for the treatment of anxiety. Psychotherapy research : journal of the Society for Psychotherapy Research 2020: 1-12.
39. Nordahl HM, Borkovec TD, Hagen R, et al. Metacognitive therapy versus cognitive-behavioural therapy in adults with generalised anxiety disorder. BJPsych Open 2018; 4(5): 393-400.
40. Orvati Aziz M, Mehrinejad SA, Hashemian K, Paivastegar M. Integrative therapy (short-term psychodynamic psychotherapy & cognitive-behavioral therapy) and cognitive-behavioral therapy in the treatment of generalized anxiety disorder: A randomized controlled trial. Complement Ther Clin Pract 2020; 39: 101122.
41. Ost LG, Breitholtz E. Applied relaxation vs. cognitive therapy in the treatment of generalized anxiety disorder. Behaviour research and therapy 2000; 38(8): 777-90.
42. Pallavicini F, Algeri D, Repetto C, Gorini A, Riva G. Biofeedback, virtual reality and mobile phones in the treatment of generalized anxiety disorder (gad): A phase-2 controlled clinical trial. Journal of Cyber Therapy and Rehabilitation 2009; 2(4): 315-27.
43. Paxling B, Almlov J, Dahlin M, et al. Guided internet-delivered cognitive behavior therapy for generalized anxiety disorder: a randomized controlled trial. Cognitive behaviour therapy 2011; 40(3): 159-73.
44. Richards D, Timulak L, Rashleigh C, et al. Effectiveness of an internet-delivered intervention for generalized anxiety disorder in routine care: A randomised controlled trial in a student population. Internet Interventions 2016; 6: 80-8.
45. Robinson E, Titov N, Andrews G, McIntyre K, Schwencke G, Solley K. Internet treatment for generalized anxiety disorder: a randomized controlled trial comparing clinician vs. technician assistance. PloS one 2010; 5(6): e10942.
46. Roemer L, Orsillo SM, Salters-Pedneault K. Efficacy of an acceptance-based behavior therapy for generalized anxiety disorder: evaluation in a randomized controlled trial. Journal of consulting and clinical psychology 2008; 76(6): 1083-9.
47. Rogiers R, Baeken C, Van den Abbeele D, et al. Group Intervention 'Drop it!' Decreases Repetitive Negative Thinking in Major Depressive Disorder and/or Generalized Anxiety Disorder: A Randomised Controlled Study. Cognitive Therapy and Research 2021.
48. Roy A, Hoge EA, Abrante P, Druker S, Liu T, Brewer JA. Clinical Efficacy and Psychological Mechanisms of an App-Based Digital Therapeutic for Generalized Anxiety Disorder: Randomized Controlled Trial. Journal of Medical Internet Research 2021; 23(12).
49. Roy-Byrne P, Craske MG, Sullivan G, et al. Delivery of evidence-based treatment for multiple anxiety disorders in primary care: a randomized controlled trial. Jama 2010; 303(19): 1921-8.

© 2023 American Medical Association. All rights reserved.

50. Stanley MA, Beck JG, Glassco JD. Treatment of generalized anxiety in older adults: A preliminary comparison of cognitive-behavioral and supportive approaches. Behavior Therapy 1996; 27(4): 565-81.
51. Stanley MA, Beck JG, Novy DM, Averill PM, Swann AC, Diefenbach GJ. Cognitive-behavioral treatment of late-life generalized anxiety disorder. Journal of consulting and clinical psychology 2003; 71(2): 309-19.
52. Stanley MA, Wilson N, Shrestha S, et al. Calmer Life: A Culturally Tailored Intervention for Anxiety in Underserved Older Adults. The American journal of geriatric psychiatry : official journal of the American Association for Geriatric Psychiatry 2016; 24(8): 648-58.
53. Stanley MA, Wilson NL, Novy DM, et al. Cognitive behavior therapy for generalized anxiety disorder among older adults in primary care: a randomized clinical trial. Jama 2009; 301(14): 1460-7.
54. Timulak L, Keogh D, Chigwedere C, et al. A comparison of emotion-focused therapy and cognitive-behavioral therapy in the treatment of generalized anxiety disorder: results of a feasibility randomized controlled trial. Psychotherapy (Chicago, Ill) 2022; 59(1): 84-95.
55. Titov N, Andrews G, Robinson E, et al. Clinician-assisted Internet-based treatment is effective for generalized anxiety disorder: Randomized controlled trial. Australian and New Zealand Journal of Psychiatry 2009; 43(10): 905-12.
56. Titov N, Andrews G, Johnston L, Robinson E, Spence J. Transdiagnostic Internet treatment for anxiety disorders: A randomized controlled trial. Behaviour research and therapy 2010; 48(9): 890-9.
57. van der Heiden C, Muris P, van der Molen HT. Randomized controlled trial on the effectiveness of metacognitive therapy and intolerance-of-uncertainty therapy for generalized anxiety disorder. Behaviour research and therapy 2012; 50(2): 100-9.
58. Vera M, Obén A, Juarbe D, Hernández N, Pérez-Pedrogo C. Randomized pilot trial of cognitive-behavioral therapy and acceptance-based behavioral therapy in the treatment of Spanish-speaking Latino primary care patients with generalized anxiety disorder. Journal of Behavioral and Cognitive Therapy 2021; 31(2): 91-103.
59. Wells A, Welford M, King P, Papageorgiou C, Wisely J, Mendel E. A pilot randomized trial of metacognitive therapy vs applied relaxation in the treatment of adults with generalized anxiety disorder. Behaviour research and therapy 2010; 48(5): 429-34.
60. Wetherell JL, Afari N, Ayers CR, et al. Acceptance and Commitment Therapy for generalized anxiety disorder in older adults: a preliminary report. Behavior therapy 2011; 42(1): 127-34.
61. Wetherell JL, Gatz M, Craske MG. Treatment of generalized anxiety disorder in older adults. Journal of consulting and clinical psychology 2003; 71(1): 31-40.
62. Wong SY, Yip BH, Mak WW, et al. Mindfulness-based cognitive therapy v. group psychoeducation for people with generalised anxiety disorder: randomised controlled trial. The British journal of psychiatry : the journal of mental science 2016; 209(1): 68-75.

© 2023 American Medical Association. All rights reserved.

63. Woodward R, Jones RB. Cognitive restructuring treatment: a controlled trial with anxious patients. Behaviour research and therapy 1980; 18(5): 401-7.
64. Zargar F, Farid AAA, Atef-Vahid M, Afshar H, Maroofi M, Omranifard V. Effect of acceptance-based behavior therapy on severity of symptoms, worry and quality of life in women with generalized anxiety disorder. Iranian Journal of Psychiatry and Behavioral Sciences 2012; 6(2): 23-32.
65. Zinbarg RE, Lee JE, Yoon KL. Dyadic predictors of outcome in a cognitive-behavioral program for patients with generalized anxiety disorder in committed relationships: a "spoonful of sugar" and a dose of non-hostile criticism may help. Behaviour research and therapy 2007; 45(4): 699-713.

© 2023 American Medical Association. All rights reserved.

eAppendix G. Excluded studies, with reasons.

N	Title of the excluded study	Full explanation	Short explanation<br><br>(as reported in Appendix D)
1	Amir N, Taboas W, Montero M. Feasibility and dissemination of a computerized home-based treatment for Generalized Anxiety Disorder: a randomized clinical trial. Behaviour research and therapy 2019; 120.	Wrong intervention, biofeedback	Wrong intervention
2	Azhar MZ, Varma SL, Dharap AS. Religious psychotherapy in anxiety disorder patients. Acta psychiatrica Scandinavica 1994; 90(1): 1-3.	Wrong intervention, religious therapy	Wrong intervention
3	Bakhshani NM, Lashkaripour K, Sadjadi SA. Effectiveness of short term cognitive behavior therapy in patients with generalized anxiety disorder. Journal of Medical Sciences 2007; 7(7): 1076-81.	Study groups are not suitable for the network	Wrong comparison
4	Barlow, D. H., et al. (1984). "Panic and generalized anxiety disorders: Nature and treatment." Behavior Therapy 15(5): 431-449.	Wrong population, mixed anxiety population (GAD+Panic)	Wrong population
5	Biswas, A., & Chattopadhyay, P. K. (2001). Predicting psychotherapeutic outcomes in patients with generalised anxiety disorder. Journal of Personality and Clinical Studies, 17(1), 27–32.	wrong intervention, biofeedback	Wrong intervention
6	Blowers C, Cobb J, Mathews A. Generalised anxiety: a controlled treatment study. Behaviour research and therapy 1987; 25(6): 493-502.	Lack of data	Lack of data
7	Bond AJ, Wingrove J, Valerie Curran H, Lader MH. Treatment of generalised anxiety disorder with a short course of psychological therapy, combined with buspirone or placebo. Journal of affective disorders 2002; 72(3): 267-71.	Study groups are not suitable for the network	Wrong comparison
8	Borkovec TD, Mathews AM, Chambers A, Ebrahimi S, Lytle R, Nelson R. The effects of relaxation training with cognitive or nondirective therapy and the role of relaxation-induced anxiety in the treatment of generalized anxiety. Journal of consulting and clinical psychology 1987; 55(6): 883-8.	Study groups are not suitable for the network	Wrong comparison

© 2023 American Medical Association. All rights reserved.

9	Borkovec TD, Newman MG, Pincus AL, Lytle R. A component analysis of cognitive-behavioral therapy for generalized anxiety disorder and the role of interpersonal problems. Journal of Consulting and Clinical Psychology 2002; 70(2): 288-98.	Lack of data	Lack of data
10	Bouchard S, Dugas MJ, Belleville G, et al. A Multisite Non-Inferiority Randomized Controlled Trial of the Efficacy of Cognitive-Behavior Therapy for Generalized Anxiety Disorder Delivered by Videoconference. J Clin Med 2022; 11(19).	Study groups are not suitable for the network	Wrong comparison
11	Bouchard S, Marchand A, Gosselin P, Dugas M, Belleville G. Non-inferiority trial of the efficacy of telepsychotherapy for generalized anxiety disorder: Interim results. Journal of Cyber Therapy and Rehabilitation 2015; 8(1): 18-9.	Interim results of Bouchard 2022	Preliminary results
12	Bouchard S, Marchand A, Gosselin P, Langlois F, Dugas MG, Belleville G. Efficacy of delivering cognitivebehaviour therapy in videoconference to people suffering from generalized anxiety disorder: preliminary results of a randomized control trial. Journal of Cyber Therapy and Rehabilitation 2016; 9(1): 13.	Preliminary results of Bouchard 2022	Preliminary results
13	Brenes GA, Danhauer SC, Lyles MF, Hogan PE, Miller ME. Telephone-Delivered Cognitive Behavioral Therapy and Telephone-Delivered Nondirective Supportive Therapy for Rural Older Adults With Generalized Anxiety Disorder: A Randomized Clinical Trial. JAMA psychiatry 2015; 72(10): 1012-20.	Data from this trial have been reported in the supplementary material of Brenes 2017, which also reports on long term outcomes	Wrong outcome
14	Brenes GA, Miller ME, Williamson JD, McCall WV, Knudson M, Stanley MA. A randomized controlled trial of telephone-delivered cognitive-behavioral therapy for late-life anxiety disorders. American journal of geriatric psychiatry 2012; 20(8): 707-16.	The study is not exclusively restricted to people diagnosed with GAD, and there are no outcome data for the GAD population only.	Wrong population
15	Capobianco L, Thompson J. Group Metacognitive Therapy Versus Mindfulness Meditation Therapy in a Transdiagnostic Patient Sample: A Feasibility Study	Participants suffer from both mild anxiety and depression (Participants were assessed for suitability using the GAD-7 and PHQ-9 screening measures, and were required to score five or greater on one or both measures)	Wrong population

© 2023 American Medical Association. All rights reserved.

16	Chau SW, Tse CY, So SH, Chan SS. Attentional Bias Modification Training for Patients with Generalised Anxiety Disorder: a Randomised Controlled Study. Dong Ya jing shen ke xue zhi [East Asian archives of psychiatry] 2019; 29(1): 3-9.	Wrong intervention, attentional modification bias	Wrong intervention
17	Christensen, H., et al. (2014). "Prevention of generalized anxiety disorder using a web intervention, iChill: randomized controlled trial." J Med Internet Res 16(9): e199.	RCT is mainly about prevention. No formal GAD diagnosis. Inclusion criteria GAD 7 at least 5 (cit doi:10.1001/archinte.166.10.1092 "A score of 10 or greater on the GAD-7 represents a reasonable cut point for identifying cases of GAD."	Wrong outcome
18	Costa MA, Gonçalves FG, Tatton-Ramos T, et al. A Three-Arm Randomized Clinical Trial Comparing the Efficacy of a Mindfulness-Based Intervention with an Active Comparison Group and Fluoxetine Treatment for Adults with Generalized Anxiety Disorder. Psychotherapy and psychosomatics 2021; 90(4): 269-79.	Study groups are not suitable for the network	Wrong comparison
19	Craske MG, Stein MB, Sullivan G, et al. Disorder-specific impact of coordinated anxiety learning and management treatment for anxiety disorders in primary care. Archives of General Psychiatry 2011; 68(4): 378-88.	Secondary publication of Roy-Byrne 2010	Secondary publication
20	Crits-Christoph P, Gibbons MBC, Narducci J, Schamberger M, Gallop R. Interpersonal Problems and the Outcome of Interpersonally Oriented Psychodynamic Treatment of GAD. Psychotherapy: Theory, Research, Practice, Training 2005; 42(2): 211-24.	Wrong design	Wrong design
21	Crits-Christoph P, Newman MG, Rickels K, et al. Combined medication and cognitive therapy for generalized anxiety disorder. Journal of anxiety disorders 2011; 25(8): 1087-94.	Study groups are not suitable for the network	Wrong comparison
22	Dahlin M, Johansson A, Romare H, Carlbring P, Andersson G. Worry-specific versus self-tailored internet-based treatments for generalized anxiety disorder, with scheduled support or support on demand: a pilot factorial design trial. Internet interventions 2022; 28.	Study groups are not suitable for the network	Wrong comparison

© 2023 American Medical Association. All rights reserved.

23	Dear BF, Staples LG, Terides MD, et al. Transdiagnostic versus disorder-specific and clinician-guided versus self-guided internet-delivered treatment for generalized anxiety disorder and comorbid disorders: A randomized controlled trial. Journal of anxiety disorders 2015; 36: 63-77.	Study groups are not suitable for the network	Wrong comparison
24	Fava GA, Ruini C, Rafanelli C, et al. Well-being therapy of generalized anxiety disorder. Psychotherapy and psychosomatics 2005; 74(1): 26-30.	Study groups are not suitable for the network	Wrong comparison
25	Flückiger C, Forrer L, Schnider B, Bättig I, Bodenmann G, Zinbarg RE. A Single-blinded, Randomized Clinical Trial of How to Implement an Evidence-based Treatment for Generalized Anxiety Disorder [IMPLEMENT] - Effects of Three Different Strategies of Implementation. EBioMedicine 2016; 3: 163-71.	Study groups are not suitable for the network	Wrong comparison
26	Flückiger C, Vîslă A, Wolfer C, et al. Exploring change in cognitive-behavioral therapy for generalized anxiety disorder—A two-arms ABAB crossed-therapist randomized clinical implementation trial. Journal of Consulting and Clinical Psychology 2021; 89(5): 454-68.	ABAB crossed-therapist randomized clinical implementation trial design	Wrong design
27	Fortin-Delisle I, Marcotte-Beaumier G, Koerner N, Langlois F, Gosselin P, Dugas MJ. Modification des interprétations et thérapie cognitivo-comportementale pour le trouble d'anxiété généralisée : Un essai randomisé = Changing interpretations and cognitive behavioral therapy for generalized anxiety disorder: A randomized trial. Canadian Journal of Behavioural Science / Revue canadienne des sciences du comportement 2021; 53(1): 12-22.	Wrong intervention. This randomized clinical trial evaluates the impact of adding an Interpretation Modification Program (IMP) to CBT	Wrong intervention
28	Gaudreau C, Landreville P, Carmichael P-H, Champagne A, Camateros C. Older adults' rating of the acceptability of treatments for generalized anxiety disorder. Clinical Gerontologist: The Journal of Aging and Mental Health 2015; 38(1): 68-87.	Wrong outcome	Wrong outcome
29	Gorini A, Pallavicini F, Algeri D, Repetto C, Gaggioli A, Riva G. Virtual reality in the treatment of generalized anxiety disorders. Studies in health technology and informatics 2010; 154: 39-43.	Secondary publication of Pallavicini 2009	Secondary publication
30	Hantouche EG, Vahia VN. Interactions between psychotherapy and drug therapy in generalized anxiety disorder. Human Psychopharmacology 1999; 14(SUPPL. 1): S87-S93.	Wrong design, not an RCT	Wrong design
31	Herring MP, Jacob ML, Suveg C, Dishman RK, O'Connor PJ. Feasibility of exercise training for the short-term treatment of generalized anxiety disorder: A randomized controlled trial. Psychotherapy and Psychosomatics 2011; 81(1): 21-8.	Wrong intervention, physical activity	Wrong intervention

© 2023 American Medical Association. All rights reserved.

32	Hirsch CR, Krahé C, Whyte J, et al. Internet-delivered interpretation training reduces worry and anxiety in individuals with generalized anxiety disorder: A randomized controlled experiment. Journal of consulting and clinical psychology 2021; 89(7): 575-89.	Wrong design. flotation-REST (restricted environmental stimulation technique)	Wrong design
33	Johnston, L., et al. (2011). "A RCT of a transdiagnostic internet-delivered treatment for three anxiety disorders: examination of support roles and disorder-specific outcomes." PLoS One 6(11): e28079.	Wrong population, mixed anxiety disorders. the trial is in the "any anxiety disorder" folder	Wrong population
34	Jonsson K, Kjellgren A. Promising effects of treatment with flotation-REST (restricted environmental stimulation technique) as an intervention for generalized anxiety disorder (GAD): a randomized controlled pilot trial. BMC complementary and alternative medicine 2016; 16: 108.	Wrong intervention, floatation rest	Wrong intervention
35	Koszycki D, Raab K, Aldosary F, Bradwejn J. A multifaith spiritually based intervention for generalized anxiety disorder: a pilot randomized trial. Journal of clinical psychology 2010; 66(4): 430-41.	Wrong intervention, religious therapy	Wrong intervention
36	Koszycki D, Bilodeau C, Raab-Mayo K, Bradwejn J. A multifaith spiritually based intervention versus supportive therapy for generalized anxiety disorder: a pilot randomized controlled trial. Journal of clinical psychology 2014; 70(6): 489-509.	Wrong intervention, religious therapy	Wrong intervention
37	Lader MH, Bond AJ. Interaction of pharmacological and psychological treatments of anxiety. Br J Psychiatry Suppl 1998; (34): 42-8.	Study groups are not suitable for the network	Wrong comparison
38	LaFreniere LS, Newman MG. A BRIEF ECOLOGICAL MOMENTARY INTERVENTION FOR GENERALIZED ANXIETY DISORDER: A RANDOMIZED CONTROLLED TRIAL OF THE WORRY OUTCOME JOURNAL. Depression and anxiety 2016; 33(9): 829-39.	Wrong intervention. "Worry Outcome Journal"	Wrong intervention
39	Levy Berg A, Sandell R, Sandahl C. Affect-focused body psychotherapy in patients with generalized anxiety disorder: Evaluation of an integrative method. Journal of Psychotherapy Integration 2009; 19: 67-85.<br>17. Linden M, Zubraegel D, Baer T, Franke U, Schlattmann P. Efficacy of cognitive behaviour therapy in generalized anxiety disorders. Results of a controlled clinical trial (Berlin CBT-GAD Study). Psychotherapy and psychosomatics 2005; 74(1): 36-42.	No. study groups are not suitable for the network. Study endpoint is too delayed (conflict with the study protocol)	conflict with the study protocol

© 2023 American Medical Association. All rights reserved.

40	Linden M, Bar T, Zubragel D, Ahrens B, Schlattmann P. Effectiveness of the cognitive behavior therapy in the treatment of generalized anxiety disorders - Results of the Berlin GAD-KVT-study. Verhaltenstherapie 2002; 12(3): 173-81.	Duplicate of Linden 2005	Secondary publication
41	Linden M, Zubraegel D, Baer T, Franke U, Schlattmann P. Efficacy of cognitive behaviour therapy in generalized anxiety disorders. Results of a controlled clinical trial (Berlin CBT-GAD Study). Psychotherapy and psychosomatics 2005; 74(1): 36-42.	No. Study endpoint is too delayed (conflict with the study protocol)	conflict with the study protocol
42	Miller CB, Gu J, Henry AL, et al. Feasibility and efficacy of a digital CBT intervention for symptoms of Generalized Anxiety Disorder: A randomized multiple-baseline study. Journal of Behavior Therapy and Experimental Psychiatry 2021; 70.	Multiple-baseline design	Wrong design
43	Millstein DJ, Orsillo SM, Hayes-Skelton SA, Roemer L. Interpersonal Problems, Mindfulness, and Therapy Outcome in an Acceptance-Based Behavior Therapy for Generalized Anxiety Disorder. Cognitive behaviour therapy 2015; 44(6): 491-501.	Secondary publication of Hayes-Skelton 2013	Secondary publication
44	Newman MG, Castonguay LG, Borkovec TD, et al. A randomized controlled trial of cognitive-behavioral therapy for generalized anxiety disorder with integrated techniques from emotion-focused and interpersonal therapies. Journal of consulting and clinical psychology 2011; 79(2): 171-81.	Study groups are not suitable for the network	Wrong comparison
45	Newman MG, Przeworski A, Consoli AJ, Taylor CB. A randomized controlled trial of ecological momentary intervention plus brief group therapy for generalized anxiety disorder. Psychotherapy (Chicago, Ill) 2014; 51(2): 198-206.	Study groups are not suitable for the network	Wrong comparison
46	Newman MG, Kanuri N, Rackoff GN, Jacobson NC, Bell MJ, Taylor CB. A Randomized Controlled Feasibility Trial of Internet-Delivered Guided Self-Help for Generalized Anxiety Disorder (GAD) Among University Students in India. Psychotherapy 2021; 58(4): 591-601.	students classified as having clinical or subthreshold GAD	Wrong population
47	Olivares-Olivares PJ, Olivares J, Macia D, Macia A, Montesinos L. Community versus clinical cognitive-behavioral intervention in young-adult Spanish population with generalized social phobia. Terapia psicologica 2016; 34(1): 23-30	Wrong diagnosis, social phobia	Wrong population
48	Power KG, Jerrom DWA, Simpson RJ, Mitchell MJ, Swanson V. A controlled comparison of Cognitive-Behaviour Therapy, Diazepam and Placebo in the management of generalized anxiety. Behavioural Psychotherapy 1989; 17(1): 1-14	Study groups are not suitable for the network	Wrong comparison
49	Power KG, Simpson RJ, Swanson V, Wallace LA. Controlled comparison of pharmacological and psychological treatment of generalized anxiety disorder in primary care. The British	Study groups are not suitable for the network	Wrong comparison

© 2023 American Medical Association. All rights reserved.

	journal of general practice : the journal of the Royal College of General Practitioners 1990; 40(336): 289-94.		
50	Razali SM, Aminah K, Khan UA. Religious-cultural psychotherapy in the management of anxiety patients. Transcultural Psychiatry 2002; 39(1): 130-6.	Wrong population	Wrong population
51	Sarkar, P., Rathee, S. P., & Neera, N. (1999). Comparative efficacy of pharmacotherapy and bio-feed back among cases of generalised anxiety disorder. Journal of Projective Psychology & Mental Health, 6(1), 69–77.	Wrong intervention, biofeedback	Wrong intervention
52	Simon NM, Hofmann SG, Rosenfield D, et al. Efficacy of yoga vs cognitive behavioral therapy vs stress education for the treatment of generalized anxiety disorder: A randomized clinical trial. JAMA psychiatry 2020.	Lack of data	Lack of data
53	Teng MH, Hou YM, Chang SH, Cheng HJ. Home-delivered attention bias modification training via smartphone to improve attention control in sub-clinical generalized anxiety disorder: a randomized, controlled multi-session experiment. Journal of affective disorders 2019; 246: 444-51.	Wrong intervention, Attention bias modification	Wrong intervention
54	Treanor M, Erisman SM, Salters-Pedneault K, Roemer L, Orsillo SM. Acceptance-based behavioral therapy for GAD: effects on outcomes from three theoretical models. Depression and anxiety 2011; 28(2): 127-36.	Secondary publication of Roemer 2008	Secondary publication
55	Westra HA, Arkowitz H, Dozois DJ. Adding a motivational interviewing pretreatment to cognitive behavioral therapy for generalized anxiety disorder: a preliminary randomized controlled trial. Journal of anxiety disorders 2009; 23(8): 1106-17.	Wrong intervention, adding a motivational interview before CBT or not	Wrong intervention
56	Westra HA, Constantino MJ, Antony MM. Integrating motivational interviewing with cognitive-behavioral therapy for severe generalized anxiety disorder: An allegiance-controlled randomized clinical trial. Journal of consulting and clinical psychology 2016; 84(9): 768-82.	Study groups are not suitable for the network	Wrong comparison
57	White J, Keenan M, Brooks N. Stress Control: A Controlled Comparative Investigation of Large Group Therapy for Generalized Anxiety Disorder. Behavioural Psychotherapy 1992; 20(2): 97-113.	Wrong design. Not randomized. quote "Following an individual assessment interview, patients were referred in "batches" to whichever therapy course was scheduled next.	Wrong design

© 2023 American Medical Association. All rights reserved.

58	Zemestani M, Beheshti N, Rezaei F, Van Der Heiden C, Kendall PC. Cognitive Behavior Therapy Targeting Intolerance of Uncertainty Versus Selective Serotonin Reuptake Inhibitor for Generalized Anxiety Disorder: A Randomized Clinical Trial. Behaviour Change 2021.	Study groups are not suitable for the network	Wrong comparison
59	Zargar F, Farid AAA, Atef-Vahid MK, Afshar H, Omidi A. Comparing the effectiveness of acceptance-based behavior therapy and applied relaxation on acceptance of internal experiences, engagement in valued actions and quality of life in generalized anxiety disorder. Journal of Research in Medical Sciences 2013; 18(2): 118-22.	Secondary publication of the above Zargar 2012	Secondary publication
60	Zhang Y, Young D, Lee S, et al. Chinese Taoist cognitive psychotherapy in the treatment of generalized anxiety disorder in contemporary China. Transcultural Psychiatry 2002; 39(1): 115-29.	Study groups are not suitable for the network	Wrong comparison

© 2023 American Medical Association. All rights reserved.

eAppendix H. Risk of bias evaluation

Risk of bias 2 summary

	Study ID	D1	D2	D3	D4	D5	Overall
1	Afshari, 2020	!	!	+	+	!	!
2	Afshari, 2022	!	!	+	+	+	!
3	Alavi, 2020	!	!	+	+	!	!
4	Andersson, 2012	+	-	!	+	+	-
5	Arch, 2012	!	!	!	+	!	!
6	Arntz, 2003	-	-	!	+	!	-
7	Artiran, 2022	!	!	!	+	!	!
8	Avdagic, 2014	+	+	+	+	+	+
9	Barlow, 1992	!	-	-	-	!	-
10	Berger, 2017	+	+	+	+	+	+
11	Borkovec, 1993	!	!	+	+	!	!
12	Bowman, 1997	-	-	+	+	!	-
13	Brenes, 2017	+	!	+	+	+	!
14	Butler 1987	!	-	+	+	!	-

© 2023 American Medical Association. All rights reserved.

	Study ID	D1	D2	D3	D4	D5	Overall
15	Butler, 1991	!	+	+	+	!	!
16	Carl, 2020	+	+	+	+	+	+
17	Cragan, 1984	-	!	+	+	!	-
18	Dahlin, 2016	+	!	+	+	+	!
19	de Almeida Sampaio, 2020	+	+	-	+	!	-
20	Dugas, 2003	!	+	+	+	!	!
21	Dugas, 2010	+	+	+	+	!	!
22	Dugas, 2022	!	!	!	+	+	!
23	Durham, 1987	-	-	-	!	!	-
24	Durham, 1994	-	-	-	+	!	-
25	Erickson, 2007	!	-	!	+	!	-
26	Hayes-Skelton, 2013	+	!	-	+	!	-
27	Hoge, 2013	!	+	+	+	!	!
28	Hoyer, 2009	!	-	+	+	!	-
29	Hui, 2017	!	!	+	+	!	!

© 2023 American Medical Association. All rights reserved.

	Study ID	D1	D2	D3	D4	D5	Overall
30	Jiang, 2022	+	+	+	+	+	+
31	Jones, 2016	+	+	+	+	+	+
32	Ladouceur, 2000	!	+	+	+	!	!
33	Leichsenring, 2009	!	+	+	+	+	!
34	Lindsay, 1987	!	-	-	+	!	-
35	Lorian, 2012	!	+	+	+	+	!
36	Menning, 2018	!	+	+	+	+	!
37	Mohlman, 2003	!	-	-	+	-	-
38	Newman, 2020	+	!	-	+	!	-
39	Nordahl, 2018	+	+	+	+	+	+
40	Orvati Aziz, 2020	!	-	!	!	!	-
41	Ost, 2000	!	!	+	+	!	!
42	Pallavicini, 2009	!	-	!	-	!	-
43	Paxling, 2011	+	!	+	+	!	!
44	Richards, 2016	+	+	+	+	+	+

© 2023 American Medical Association. All rights reserved.

	Study ID	D1	D2	D3	D4	D5	Overall
45	Robinson, 2010	+	+	+	+	+	+
46	Roemer, 2008	+	+	+	+	!	!
47	Rogiers, 2021	!	+	+	+	+	!
48	Roy, 2021	!	!	+	+	!	!
49	Roy-Byrne, 2010	+	+	!	+	!	!
50	Stanley, 1996	!	-	-	!	!	-
51	Stanley, 2003	-	-	-	+	!	-
52	Stanley, 2009	+	+	+	+	+	+
53	Stanley, 2016	!	!	+	+	!	!
54	Timulak 2022	!	+	!	+	+	!
55	Titov, 2009	+	+	+	+	+	+
56	Titov, 2010	+	+	+	+	+	+
57	Van der Heiden, 2012	!	+	+	+	+	!
58	Vera, 2021	+	-	-	+	!	-
59	Wells, 2010	+	!	+	+	!	!

© 2023 American Medical Association. All rights reserved.

	Study ID	D1	D2	D3	D4	D5	Overall
60	Wetherell, 2003	!	-	-	+	!	-
61	Wetherell, 2011	-	-	-	-	!	-
62	Wong, 2016	+	!	!	+	+	!
63	Woodward, 1980	!	!	+	+	!	!
64	Zargar, 2012	!	-	-	+	!	-
65	Zinbarg, 2007	!	!	+	+	!	!

+	Low risk	D1	Randomisation process
!	Some concerns	D2	Deviations from the intended interventions
-	High risk	D3	Missing outcome data
		D4	Measurement of the outcome
		D5	Selection of the reported result

© 2023 American Medical Association. All rights reserved.

Risk of bias 2 graph

review authors' judgements about each risk of bias item presented as percentages across all included studies

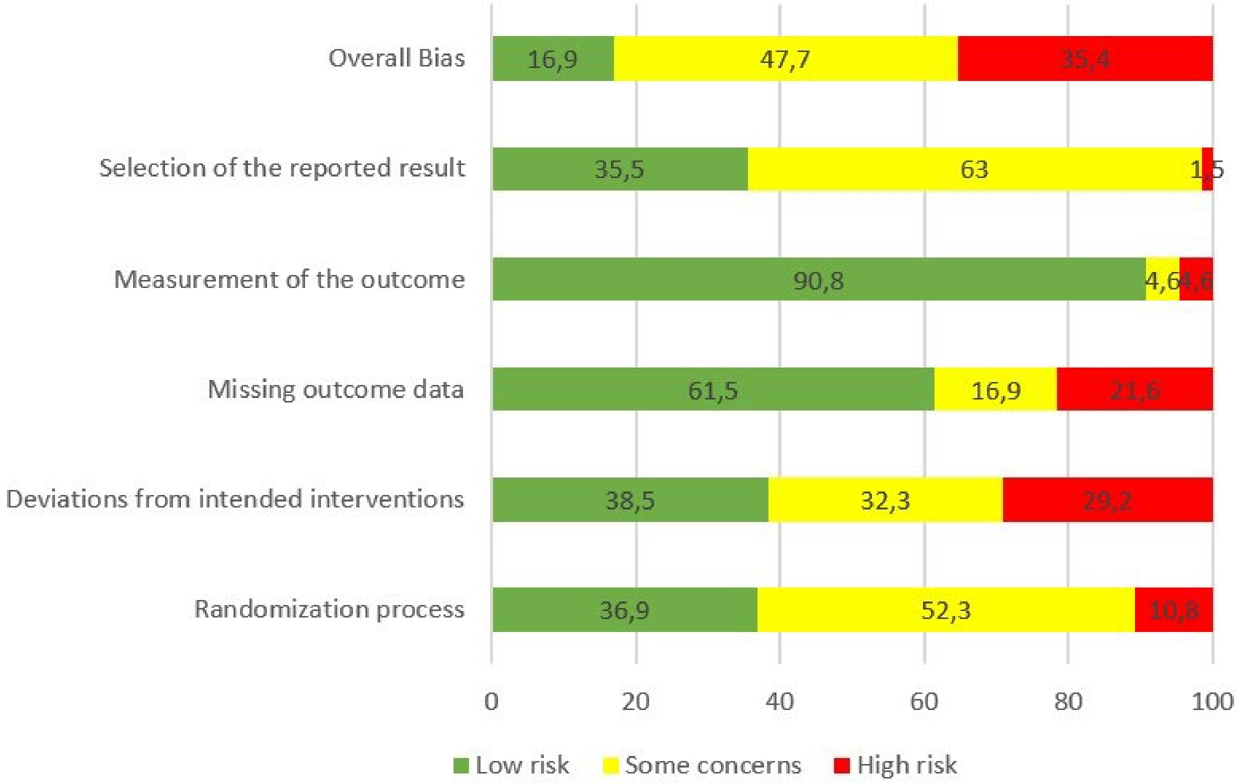

© 2023 American Medical Association. All rights reserved.

eAppendix I. Transitivity assessment and meta-regression

	HETEROGENEITY<br><br>P-value by treatment delivery formats	TRASITIVITY<br><br>P-value by set of different interventions
	meta-regression (association with outcome)	Kruskal-Wallis equality-of-populations rank test
Mean age	0.25	0.74
Percentage of women	0.89	0.034
Baseline symptomatology	0.73	0.62
number of psychotherapy sessions	0.03	0.68

Interpretation: The meaning of the Kruskal-Wallis test (KW) finding on the “percentage of women” is to be interpret as a slight imbalance of such trial-level characteristic across the comparisons, i.e. there are RCTs contributing to specific pairwise comparisons in which the percentage of women is slightly different than the average of the other pairwise comparisons. However, this fact does not influence the outcome result (meta-regression p = 0.89). Vice versa, the number of therapy sessions is evenly distributed across treatment comparisons (KW p = 0.68) and the meta-regression is borderline for signaling the influence of the n of session on outcome result (p = 0.03 – the more the n of session the better the outcome). This indication is intuitive, but given the test result, it should be interpreted as a trend rather than a meaningful finding.

MEAN AGE

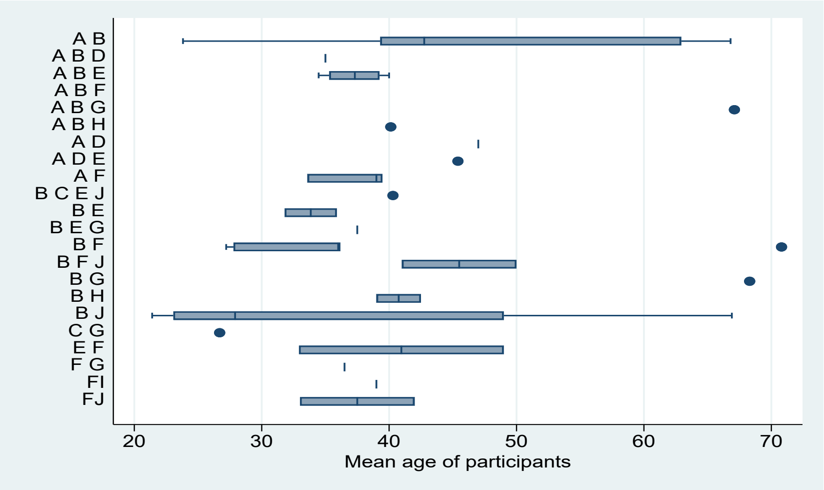

© 2023 American Medical Association. All rights reserved.

PERCENTAGE OF WOMEN

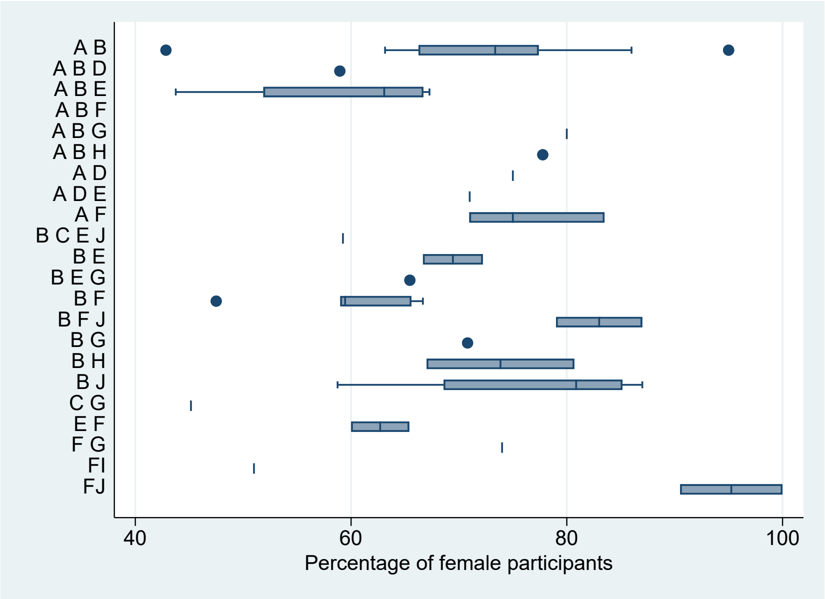

MEAN BASELINE SIMPTOMATOLOGY

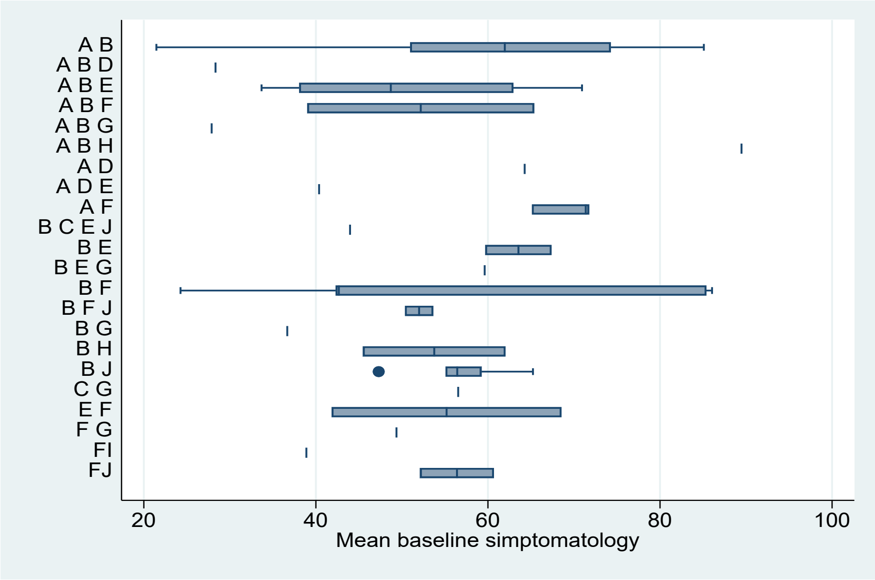

© 2023 American Medical Association. All rights reserved.

MEAN NUMBER OF PSYCHOTHERAPY SESSIONS

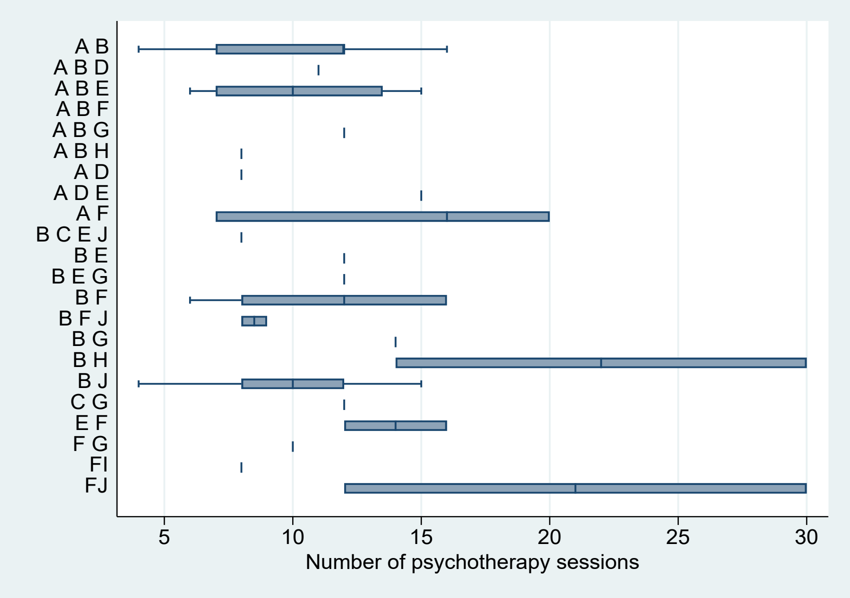

© 2023 American Medical Association. All rights reserved.

eAppendix J. Primary outcome: efficacy

Treatment codes

WL	1	A
CBT	2	B
CR	3	C
BT	4	D
RT	5	E
3W	6	F
SP	7	G
PD	8	H
PE	9	I
TAU	10	J

BT=behaviour therapy; CBT=cognitive-behaviour therapy; CR=cognitive restructuring; PE=psychoeducation; RT=relaxation therapy; SP=supportive therapy; DYN= psychodynamic therapy; 3W=third-wave CBT; TAU=treatment as usual; WL=waiting list.

Network map

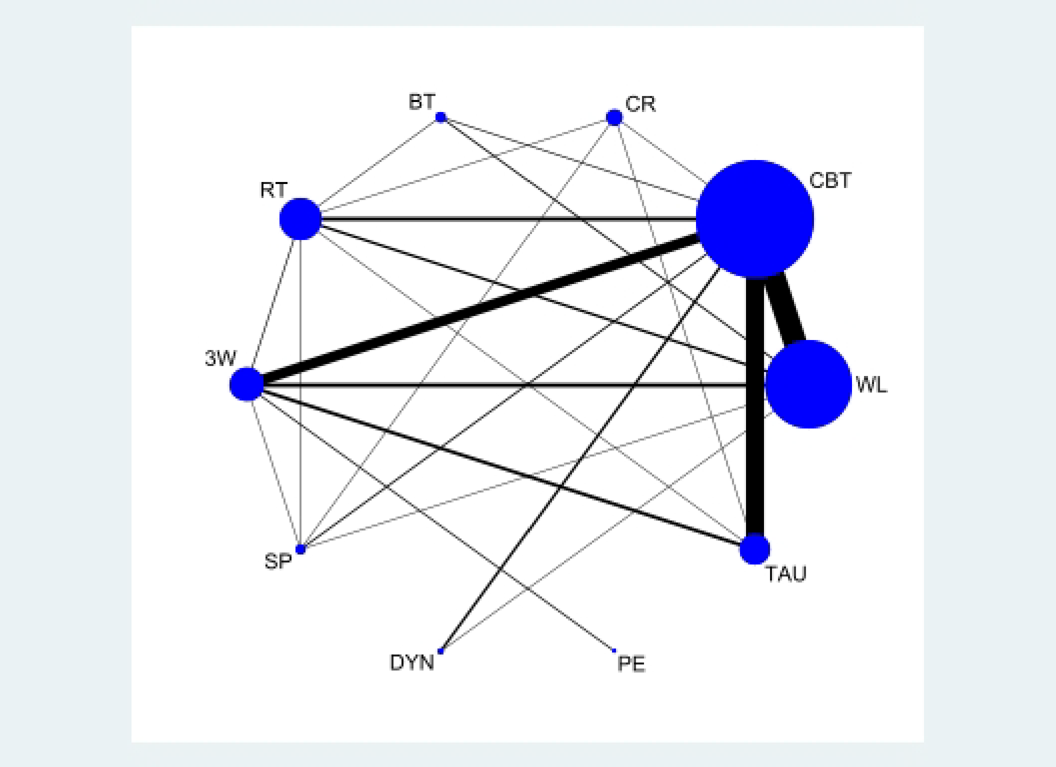

Pairwise meta-analysis

```
            Study      |      ES    [95% Conf. Interval]
-----------------------+------------------------------------------------
      F - B
Afshari, 2020          |    1.176     0.638     1.714
Afshari, 2022          |    1.587     1.033     2.141
```

© 2023 American Medical Association. All rights reserved.

Arch, 2012	-0.559	-1.348	0.230
Avdagic, 2014	-0.085	-0.721	0.551
Jiang, 2022	-0.255	-0.624	0.113
Nordahl, 2018	-0.488	-1.001	0.025
Timulak, 2022	0.614	0.086	1.142
Van der Heiden, 2012	-0.275	-0.658	0.107
Vera, 2021	-0.156	-0.748	0.435
Wetherell, 2011	-0.485	-1.555	0.586
Wong, 2016	0.107	-0.248	0.462
Sub-total			
D+L pooled ES	0.130	-0.266	0.525
J - B			
Alavi, 2020	1.676	1.142	2.211
Newman, 2020	0.315	-0.122	0.751
Orvati Aziz, 2020	2.480	1.361	3.600
Roy-Byrne, 2010	0.336	0.192	0.479
Stanley, 2009	0.322	-0.050	0.693
Vera, 2021	-0.057	-0.621	0.506
Wong, 2016	0.641	0.272	1.010
Woodward, 1980	0.736	-0.378	1.849
Sub-total			
D+L pooled ES	0.670	0.303	1.037
B - A			
Andersson, 2012	0.003	-0.558	0.564
Barlow, 1992	-1.640	-2.463	-0.818
Berger, 2017	-1.753	-2.364	-1.141
Bowman, 1997	-1.060	-1.773	-0.347
Butler, 1987	-0.594	-1.223	0.034
Butler, 1991	-0.901	-1.561	-0.242
Carl, 2020	-0.681	-0.933	-0.429
Cragan, 1984	-1.256	-1.955	-0.557
Dugas, 2010	-1.360	-2.007	-0.714
Dugas, 2003	-1.263	-1.862	-0.663
Dugas, 2022	-2.263	-2.985	-1.540
Erickson, 2007	0.403	-0.659	1.465
Hui, 2017	-1.780	-2.369	-1.192
Jones, 2016	-0.816	-1.457	-0.174
Ladouceur, 2000	-1.996	-2.964	-1.027
Lindsay, 1987	-1.169	-2.103	-0.235
Lorian, 2012	-1.257	-1.910	-0.603
Mohlman, 2003	-0.189	-1.047	0.670
Nordahl, 2018	-1.102	-1.695	-0.510
Paxling, 2011	-1.073	-1.539	-0.608
Richards, 2016	-0.324	-0.662	0.013
Robinson, 2010	-1.263	-1.639	-0.887
Rogiers, 2021	-0.566	-1.017	-0.116
Stanley, 2003	-0.986	-1.509	-0.464
Stanley, 2016	-0.594	-1.265	0.076
Titov, 2009	-1.217	-1.859	-0.575
Titov, 2010	-0.095	-0.769	0.579
Van der Heiden, 2012	-0.235	-0.751	0.282

© 2023 American Medical Association. All rights reserved.

Wetherell, 2003	-0.696	-1.339	-0.052
Zinbarg, 2007	-1.836	-2.984	-0.688
Sub-total			
D+L pooled ES	-0.962	-1.162	-0.762
H - A			
Andersson, 2012	-0.056	-0.599	0.488
Sub-total			
D+L pooled ES	-0.056	-0.599	0.488
H - B			
Andersson, 2012	-0.059	-0.620	0.502
Durham, 1994	0.681	0.174	1.188
Leichsenring, 2009	0.231	-0.291	0.752
Sub-total			
D+L pooled ES	0.297	-0.124	0.718
E - B			
Arntz, 2003	-0.430	-1.067	0.208
Barlow, 1992	-0.081	-0.819	0.657
Borkovec, 1993	0.227	-0.419	0.874
Cragan, 1984	0.581	-0.065	1.227
Dugas, 2010	0.403	-0.186	0.992
Lindsay, 1987	0.594	-0.298	1.485
Ost, 2000	0.053	-0.638	0.743
Woodward, 1980	0.172	-0.920	1.263
Sub-total			
D+L pooled ES	0.179	-0.070	0.428
G - C			
Artiran, 2022	-0.059	-0.764	0.646
Sub-total			
D+L pooled ES	-0.059	-0.764	0.646
E - A			
Barlow, 1992	-1.721	-2.677	-0.766
Cragan, 1984	-0.676	-1.343	-0.008
Dugas, 2010	-0.957	-1.586	-0.328
Hoyer, 2009	-1.114	-1.661	-0.567
Lindsay, 1987	-0.575	-1.466	0.315
Sub-total			
D+L pooled ES	-0.981	-1.296	-0.665
G - B			
Borkovec, 1993	0.909	0.240	1.579
Stanley, 1996	-0.059	-0.773	0.654
Wetherell, 2003	0.261	-0.394	0.916
Sub-total			
D+L pooled ES	0.379	-0.174	0.932
G - E			
Borkovec, 1993	0.682	0.015	1.349
Sub-total			

© 2023 American Medical Association. All rights reserved.

```
  D+L pooled ES      |   0.682     0.015     1.349
---------------------+--------------------------------------------------
     D - A
Butler, 1991         |  -0.085    -0.730     0.560
Hoyer, 2009          |  -1.117    -1.659    -0.574
Pallavicini, 2009    |  -0.271    -1.478     0.937
 Sub-total           |
  D+L pooled ES      |  -0.544    -1.299     0.211
---------------------+--------------------------------------------------
     D - B
Butler, 1991         |   0.816     0.153     1.480
Durham, 1987         |   0.249    -0.373     0.872
 Sub-total           |
  D+L pooled ES      |   0.521    -0.034     1.076
---------------------+--------------------------------------------------
     F - A
Dahlin, 2016         |  -0.976    -1.427    -0.525
Mennin, 2018         |  -2.181    -2.871    -1.490
Nordahl, 2018        |  -1.591    -2.196    -0.985
Roemer, 2008         |  -1.644    -2.473    -0.816
Van der Heiden, 2012 |  -0.510    -1.027     0.007
 Sub-total           |
  D+L pooled ES      |  -1.340    -1.911    -0.770
---------------------+--------------------------------------------------
     G - F
de Almeida Sampaio,  |   0.614     0.007     1.221
 Sub-total           |
  D+L pooled ES      |   0.614     0.007     1.221
---------------------+--------------------------------------------------
     F - E
Hayes-Skelton, 2013  |   0.220    -0.276     0.716
Wells, 2010          |  -1.237    -2.218    -0.257
 Sub-total           |
  D+L pooled ES      |  -0.445    -1.867     0.978
---------------------+--------------------------------------------------
     I - F
Hoge, 2013           |   0.364    -0.056     0.785
 Sub-total           |
  D+L pooled ES      |   0.364    -0.056     0.785
---------------------+--------------------------------------------------
     E - D
Hoyer, 2009          |   0.002    -0.517     0.522
 Sub-total           |
  D+L pooled ES      |   0.002    -0.517     0.522
---------------------+--------------------------------------------------
     J - F
Roy, 2021            |   1.512     0.936     2.088
Vera, 2021           |   0.099    -0.464     0.662
Wong, 2016           |   0.534     0.167     0.901
Zargar, 2012         |   0.751    -0.216     1.718
 Sub-total           |
  D+L pooled ES      |   0.712     0.121     1.302
---------------------+--------------------------------------------------
```

© 2023 American Medical Association. All rights reserved.

```
     G - A
Wetherell, 2003      |   -0.435    -1.070     0.200
 Sub-total           |
  D+L pooled ES      |   -0.435    -1.070     0.200
---------------------+--------------------------------------------------
     C - B
Woodward, 1980       |    0.589    -0.517     1.694
 Sub-total           |
  D+L pooled ES      |    0.589    -0.517     1.694
---------------------+--------------------------------------------------
     E - C
Woodward, 1980       |   -0.417    -1.472     0.639
 Sub-total           |
  D+L pooled ES      |   -0.417    -1.472     0.639
---------------------+--------------------------------------------------
     J - C
Woodward, 1980       |    0.147    -0.901     1.196
 Sub-total           |
  D+L pooled ES      |    0.147    -0.901     1.196
---------------------+--------------------------------------------------
     J - E
Woodward, 1980       |    0.564    -0.498     1.626
 Sub-total           |
  D+L pooled ES      |    0.564    -0.498     1.626
---------------------+--------------------------------------------------

Test(s) of heterogeneity:
               Heterogeneity  degrees of
                 statistic     freedom      P    I-squared**   Tau-
squared

F - B              63.84         10      0.000      84.3%       0.3614
J - B              40.45          7      0.000      82.7%       0.1985
B - A             100.37         29      0.000      71.1%       0.2041
H - A               0.00          0        .          .%        0.0000
H - B               3.80          2      0.150      47.4%       0.0657
E - B               7.00          7      0.429       0.0%       0.0000
G - C               0.00          0        .          .%        0.0000
E - A               4.14          4      0.387       3.4%       0.0045
G - B               3.99          2      0.136      49.8%       0.1191
G - E               0.00          0        .          .%        0.0000
D - A               6.17          2      0.046      67.6%       0.2897
D - B               1.49          1      0.222      32.9%       0.0528
F - A              18.25          4      0.001      78.1%       0.3240
G - F               0.00          0        .          .%        0.0000
F - E               6.76          1      0.009      85.2%       0.9045
I - F               0.00          0        .          .%        0.0000
E - D               0.00          0        .          .%        0.0000
J - F              12.70          3      0.005      76.4%       0.2652
G - A               0.00          0        .          .%        0.0000
C - B               0.00          0        .          .%        0.0000
E - C               0.00          0        .          .%        0.0000
J - C               0.00          0        .          .%        0.0000
```

© 2023 American Medical Association. All rights reserved.

```
J - E                0.00         0        .        .%      0.0000
** I-squared: the variation in ES attributable to heterogeneity)
```

```
Significance test(s) of ES=0

F - B                 z=  0.64     p = 0.521
J - B                 z=  3.58     p = 0.000
B - A                 z=  9.43     p = 0.000
H - A                 z=  0.20     p = 0.841
H - B                 z=  1.38     p = 0.167
E - B                 z=  1.41     p = 0.158
G - C                 z=  0.16     p = 0.870
E - A                 z=  6.09     p = 0.000
G - B                 z=  1.34     p = 0.180
G - E                 z=  2.00     p = 0.045
D - A                 z=  1.41     p = 0.158
D - B                 z=  1.84     p = 0.066
F - A                 z=  4.60     p = 0.000
G - F                 z=  1.98     p = 0.047
F - E                 z=  0.61     p = 0.540
I - F                 z=  1.70     p = 0.089
E - D                 z=  0.01     p = 0.992
J - F                 z=  2.36     p = 0.018
G - A                 z=  1.34     p = 0.180
C - B                 z=  1.04     p = 0.297
E - C                 z=  0.77     p = 0.439
J - C                 z=  0.28     p = 0.783
J - E                 z=  1.04     p = 0.298
```

Interval plot

© 2023 American Medical Association. All rights reserved.

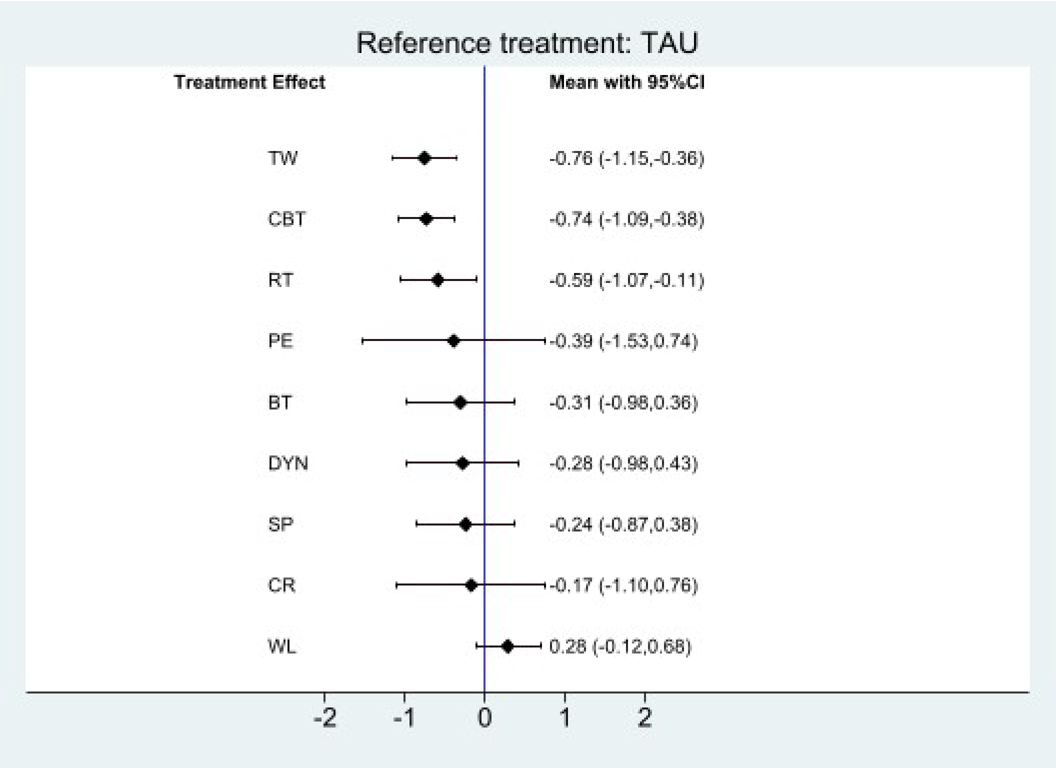

BT=behaviour therapy; CBT=cognitive-behaviour therapy; CR=cognitive restructuring; PE=psychoeducation; RT=relaxation therapy; SP=supportive therapy; DYN= psychodynamic therapy; 3W=third-wave CBT; TAU=treatment as usual; WL=waiting list.
CI=confidence interval.

Net league table

BT	-0.43 (-1.00,0.14)	0.14 (-0.91,1.19)	0.03 (-0.80,0.86)	-0.08 (-1.32,1.15)	-0.28 (-0.91,0.35)	0.07 (-0.70,0.83)	-0.45 (-1.07,0.17)	0.31 (-0.36,0.98)	0.59 (0.02,1.16)
0.43 (-0.14,1.00)	CBT	0.57 (-0.32,1.46)	0.46 (-0.15,1.07)	0.34 (-0.76,1.44)	0.15 (-0.20,0.49)	0.49 (-0.04,1.02)	-0.02 (-0.29,0.25)	0.74 (0.38,1.09)	1.02 (0.82,1.22)
-0.14 (-1.19,0.91)	-0.57 (-1.46,0.32)	CR	-0.11 (-1.19,0.97)	-0.22 (-1.63,1.18)	-0.42 (-1.34,0.50)	-0.07 (-0.96,0.81)	-0.59 (-1.50,0.33)	0.17 (-0.76,1.10)	0.45 (-0.45,1.36)
-0.03 (-0.86,0.80)	-0.46 (-1.07,0.15)	0.11 (-0.97,1.19)	DYN	-0.12 (-1.37,1.14)	-0.31 (-1.01,0.39)	0.03 (-0.77,0.84)	-0.48 (-1.15,0.19)	0.28 (-0.43,0.98)	0.56 (-0.07,1.19)
0.08 (-1.15,1.32)	-0.34 (-1.44,0.76)	0.22 (-1.18,1.63)	0.12 (-1.14,1.37)	PE	-0.20 (-1.34,0.94)	0.15 (-1.06,1.35)	-0.36 (-1.43,0.70)	0.39 (-0.74,1.53)	0.68 (-0.43,1.78)
0.28 (-0.35,0.91)	-0.15 (-0.49,0.20)	0.42 (-0.50,1.34)	0.31 (-0.39,1.01)	0.20 (-0.94,1.34)	RT	0.35 (-0.25,0.94)	-0.17 (-0.57,0.24)	0.59 (0.11,1.07)	0.87 (0.51,1.24)
-0.07 (-0.83,0.70)	-0.49 (-1.02,0.04)	0.07 (-0.81,0.96)	-0.03 (-0.84,0.77)	-0.15 (-1.35,1.06)	-0.35 (-0.94,0.25)	SP	-0.51 (-1.07,0.05)	0.24 (-0.38,0.87)	0.53 (-0.02,1.08)
0.45 (-0.17,1.07)	0.02 (-0.25,0.29)	0.59 (-0.33,1.50)	0.48 (-0.19,1.15)	0.36 (-0.70,1.43)	0.17 (-0.24,0.57)	0.51 (-0.05,1.07)	TW	0.76 (0.36,1.15)	1.04 (0.74,1.35)
-0.31 (-0.98,0.36)	-0.74 (-1.09,-0.38)	-0.17 (-1.10,0.76)	-0.28 (-0.98,0.43)	-0.39 (-1.53,0.74)	-0.59 (-1.07,-0.11)	-0.24 (-0.87,0.38)	-0.76 (-1.15,-0.36)	TAU	0.28 (-0.12,0.68)
-0.59 (-1.16,-0.02)	-1.02 (-1.22,-0.82)	-0.45 (-1.36,0.45)	-0.56 (-1.19,0.07)	-0.68 (-1.78,0.43)	-0.87 (-1.24,-0.51)	-0.53 (-1.08,0.02)	-1.04 (-1.35,-0.74)	-0.28 (-0.68,0.12)	WL

Net league table: head-to-head comparisons. Effect sizes (ES) and 95% confidence intervals (CIs) are reported. Negative ES favour the column-defining treatment. Statistically significant results are highlighted in boldface. BT=behaviour therapy; CBT=cognitive-behaviour therapy; CR=cognitive restructuring; PE=psychoeducation; RT=relaxation therapy; SP=supportive therapy; DYN= psychodynamic therapy; 3W=third-wave CBT; TAU=treatment as usual; WL=waiting list.

© 2023 American Medical Association. All rights reserved.

Evaluation of heterogeneity and incoherence

Overall heterogeneity
SD=0.49; Restricted likelihood ratio test for heterogeneity: LRT = 98.63 (d.f. = 1) P = 0.000

Overall incoherence
chi2(45) = 15.27
Prob > chi2 = 0.9127

Loop-specific approach

* 22 triangular loops found
* 1 quadratic loops found

Note: Heterogeneity of loop C-E-G cannot be estimated due to insufficient observations - set equal to 0

Note: Heterogeneity of loop C-E-J cannot be estimated due to insufficient observations - set equal to 0

Note: Heterogeneity of loop E-F-G cannot be estimated due to insufficient observations - set equal to 0

Evaluation of inconsistency using loop-specific heterogeneity estimates:

Loop	IF	seIF	z_value	p_value	CI_95	Loop_Heterog_tau2
A-B-H	0.650	0.622	1.044	0.297	(0.00,1.87)	0.176
A-B-F	0.476	0.362	1.316	0.188	(0.00,1.18)	0.261
B-E-G	0.472	0.497	0.950	0.342	(0.00,1.45)	0.048
A-D-E	0.379	0.623	0.609	0.543	(0.00,1.60)	0.146
B-F-G	0.370	0.822	0.450	0.653	(0.00,1.98)	0.337
B-D-E	0.339	0.391	0.868	0.385	(0.00,1.11)	0.008
C-E-G	0.324	0.731	0.443	0.658	(0.00,1.76)	0.000
B-E-F	0.304	0.547	0.555	0.579	(0.00,1.38)	0.285
A-F-G	0.291	0.968	0.301	0.764	(0.00,2.19)	0.324
A-B-E	0.263	0.347	0.757	0.449	(0.00,0.94)	0.168
E-F-J	0.227	1.061	0.214	0.830	(0.00,2.31)	0.360
B-F-J	0.180	0.527	0.342	0.732	(0.00,1.21)	0.312
A-B-G	0.161	0.701	0.229	0.819	(0.00,1.53)	0.207
B-C-G	0.151	0.878	0.172	0.864	(0.00,1.87)	0.119
E-F-G	0.145	0.513	0.283	0.777	(0.00,1.15)	0.000
A-E-G	0.136	0.508	0.268	0.789	(0.00,1.13)	0.005
A-B-D	0.117	0.572	0.204	0.839	(0.00,1.24)	0.211
C-F-G-J	0.109	1.184	0.092	0.927	(0.00,2.43)	0.265
B-E-J	0.098	0.854	0.114	0.909	(0.00,1.77)	0.157
B-C-J	0.067	1.518	0.044	0.965	(0.00,3.04)	0.208
B-C-E	0.008	0.817	0.010	0.992	(0.00,1.61)	0.020
A-E-F	0.001	0.593	0.002	0.999	(0.00,1.16)	0.248
C-E-J	.	.	.	.		0.000

*** Note: Loop C-E-J is formed only by multi-arm trial(s) - Consistent by definition

© 2023 American Medical Association. All rights reserved.

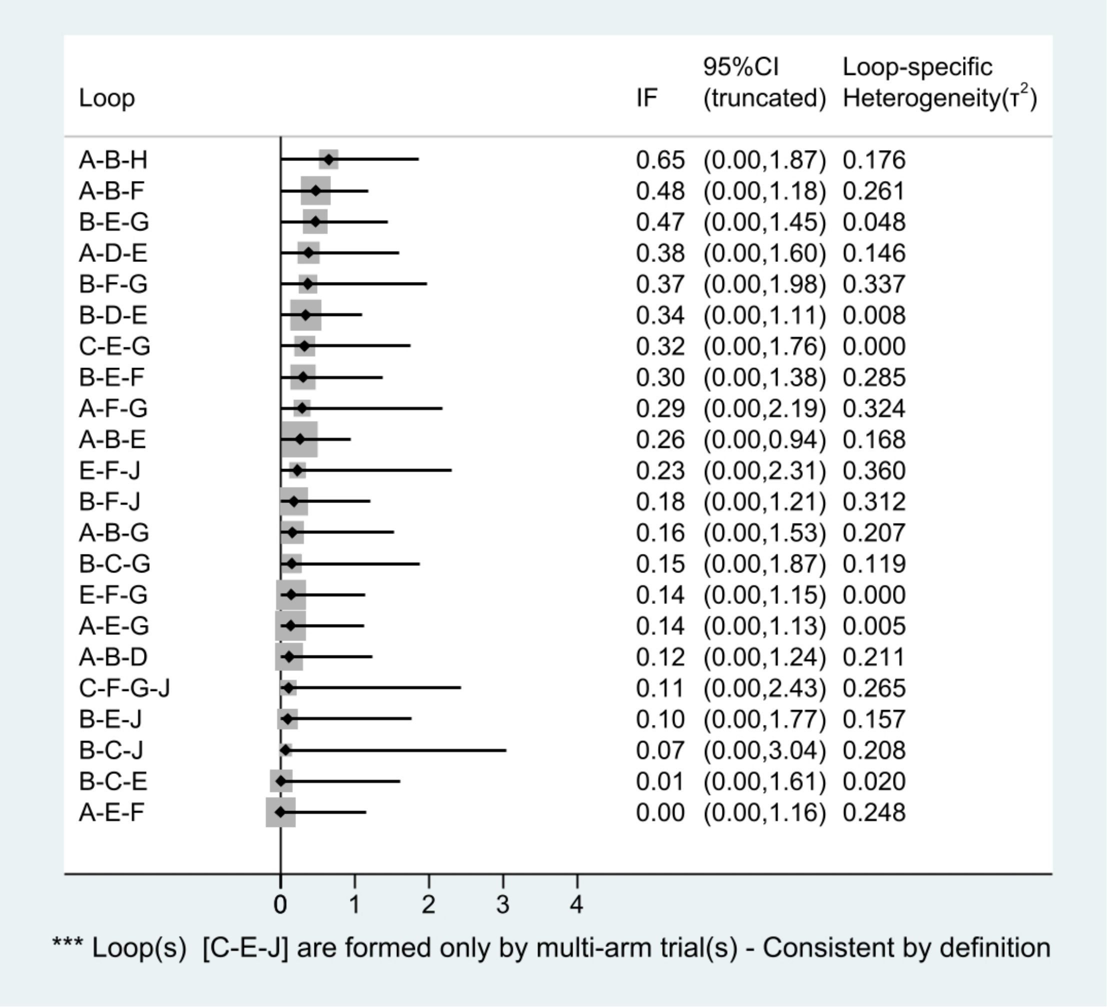

Consistency between direct and indirect estimates

Side	Direct Coef.	Std. Err.	Indirect Coef.	Std. Err.	Difference Coef.	Std. Err.	P>|z|
A B	-.9631006	.108414	-1.406193	.2827648	.4430929	.3023764	0.143
A D	-.5485477	.3691137	-.6674622	.4856404	.1189145	.6108494	0.846
A E	-.9832042	.2802767	-.7847711	.2538307	-.1984331	.3797859	0.601
A F	-1.341188	.2616791	-.8790337	.191479	-.4621541	.3246103	0.155
A G	-.4327963	.5999629	-.5543382	.3189922	.1215418	.6790615	0.858
A H	-.0555368	.5712568	-.7956729	.3888743	.7401361	.6910508	0.284
B C	.5892109	.7556374	.552452	.5797427	.0367589	.9607624	0.969
B D	.5290267	.4251715	.3357164	.4057307	.1933103	.5882232	0.742
B E	.1912513	.2216998	.0645384	.2961784	.1267129	.3700029	0.732
B F	.1339032	.1726411	-.2890618	.2272316	.4229649	.2853069	0.138
B G	.375863	.3529508	.6609827	.424238	-.2851196	.5515469	0.605
B H *	.2898853	.323306	2.047863	.9998311	-1.757978	1.052351	0.095
B J	.6844249	.2059606	.936695	.4036628	-.2522701	.4540428	0.578
C E	-.4169161	.7373482	-.4228759	.6108257	.0059598	.9573875	0.995
C G	-.0589389	.6191759	-.0910317	.6698162	.0320929	.9121592	0.972
C J	.1470866	.7349292	.1878667	.6244093	-.0407801	.9654789	0.966
D E	.0034873	.5688329	-.4160823	.3897124	.4195696	.6896554	0.543
E F	-.3443547	.4430162	-.1180558	.2336293	-.2262989	.5007228	0.651
E G	.678126	.6074114	.2369329	.3501488	.4411931	.7005398	0.529
E J	.563709	.7395672	.594566	.2603181	-.030857	.7840525	0.969
F G	.6144237	.59228	.4829355	.32987	.1314882	.677945	0.846
F I *	.3644483	.5437902	2.446519	200.5703	-2.082071	200.5708	0.992
F J	.7091945	.2973425	.8031837	.281225	-.0939891	.4091592	0.818

© 2023 American Medical Association. All rights reserved.

SUCRA and cumulative probability plots

Estimated probabilities (%) of each treatment being the best (and other ranks)
- assuming the minimum parameter is the best
- using 5000 draws
- allowing for parameter uncertainty

id_study and Rank	Treatment 1	2	3	4	5	6	7	8	9	10
1										
Best	0.0	22.4	6.2	3.4	8.3	33.6	1.2	3.5	21.3	0.0
2nd	0.0	37.7	3.5	3.4	11.6	33.2	1.4	3.3	5.7	0.0
3rd	0.0	26.7	4.1	6.2	24.3	21.4	3.8	6.1	7.4	0.1
4th	0.0	10.6	7.3	13.4	28.8	8.5	9.7	11.7	9.3	0.8
5th	0.0	2.3	10.8	19.3	17.8	2.7	17.5	16.3	9.8	3.5
6th	0.2	0.4	13.6	18.0	6.6	0.6	22.5	17.3	10.1	10.7
7th	1.0	0.0	14.5	16.3	1.9	0.1	20.7	16.5	8.9	20.0
8th	6.8	0.0	13.7	11.9	0.5	0.0	15.0	12.9	9.0	30.2
9th	28.4	0.0	11.8	6.8	0.0	0.0	6.5	9.0	7.9	29.6
Worst	63.6	0.0	14.3	1.3	0.0	0.0	1.6	3.3	10.7	5.3
MEAN RANK	9.5	2.3	6.5	5.7	3.7	2.2	6.2	5.9	5.1	7.9
SUCRA	0.1	0.9	0.4	0.5	0.7	0.9	0.4	0.5	0.5	0.2

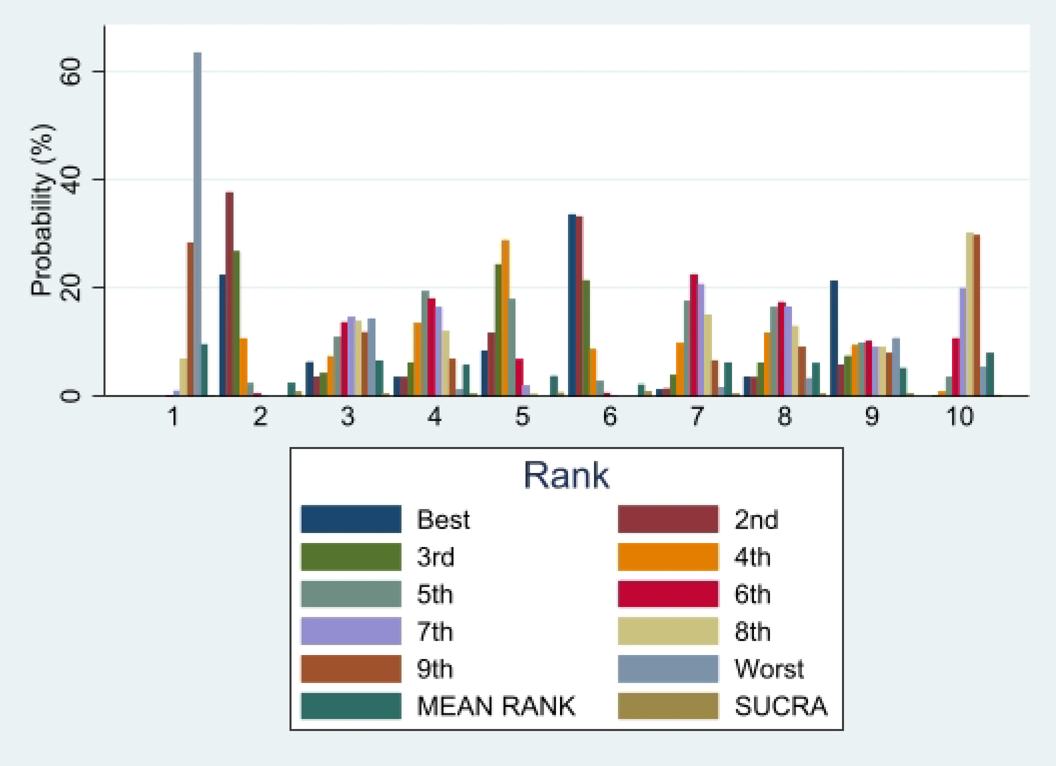

© 2023 American Medical Association. All rights reserved.

Treatment Relative Ranking of Model 1

Treatm~t	SUCRA	PrBest	MeanRank
WL	5.1	0.0	9.5
CBT	85.1	22.4	2.3
CR	38.7	6.2	6.5
BT	47.7	3.4	5.7
RT	70.4	8.3	3.7
TW	87.2	33.6	2.2
SP	42.6	1.2	6.2
DYN	45.2	3.5	5.9
PE	54.1	21.3	5.1
TAU	23.9	0.0	7.9

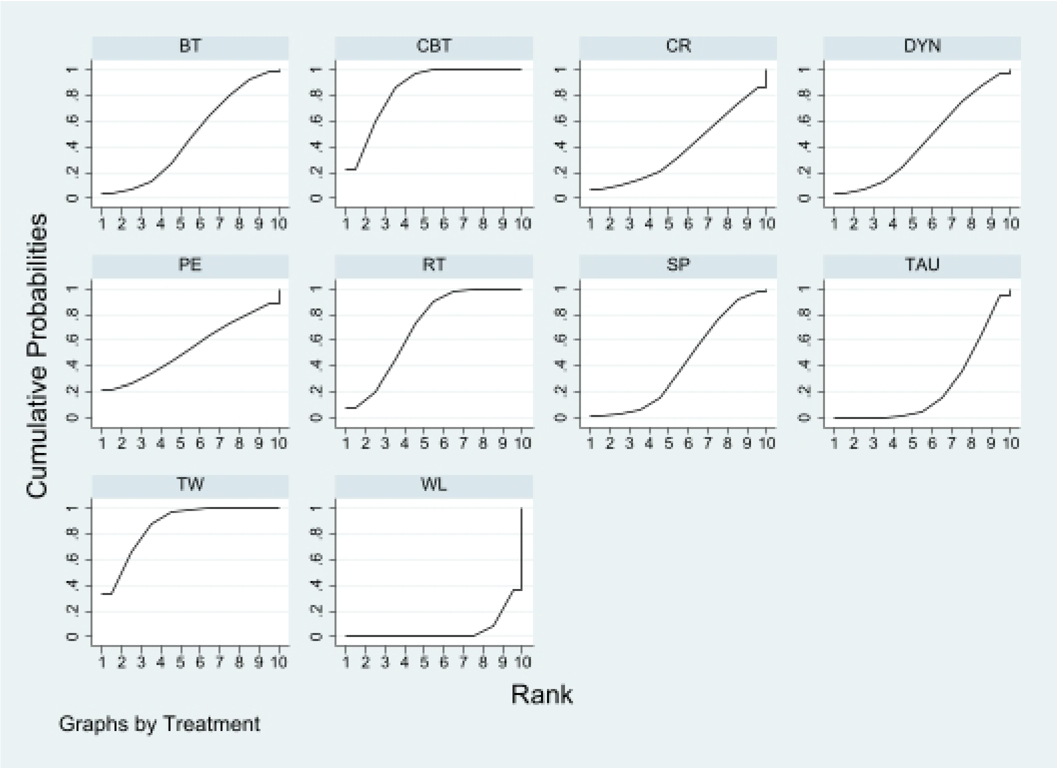

© 2023 American Medical Association. All rights reserved.

Funnel plot

2 comparisons with 10 one more studies: CBT-WL (30 studies); 3W-CBT (11 studies);

1) CBT-WL (30 studies)

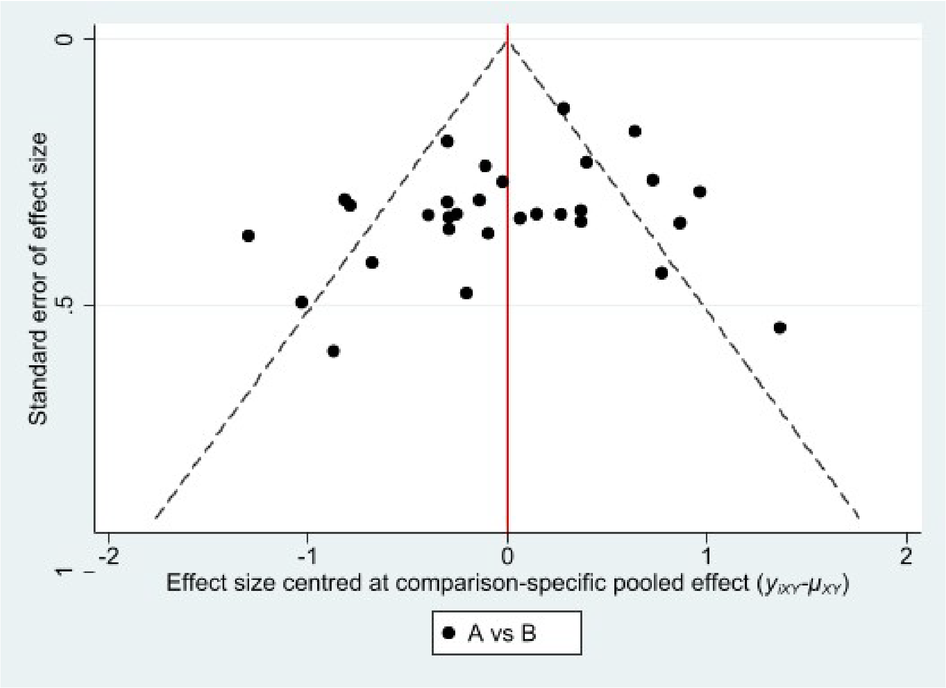

Egger's test for small-study effects:
Regress standard normal deviate of intervention
effect estimate against its standard error

```
Number of studies =   30                                  Root MSE      =    1.802
------------------------------------------------------------------------------
     Std_Eff |      Coef.   Std. Err.      t    P>|t|     [95% Conf. Interval]
-------------+----------------------------------------------------------------
       slope |  -.4440828   .2726045    -1.63   0.115    -1.002488    .1143221
        bias |  -1.638153   .9597919    -1.71   0.099    -3.604198    .3278916
------------------------------------------------------------------------------

Test of H0: no small-study effects          P = 0.099
```

© 2023 American Medical Association. All rights reserved.

2) 3W-CBT (11 studies)

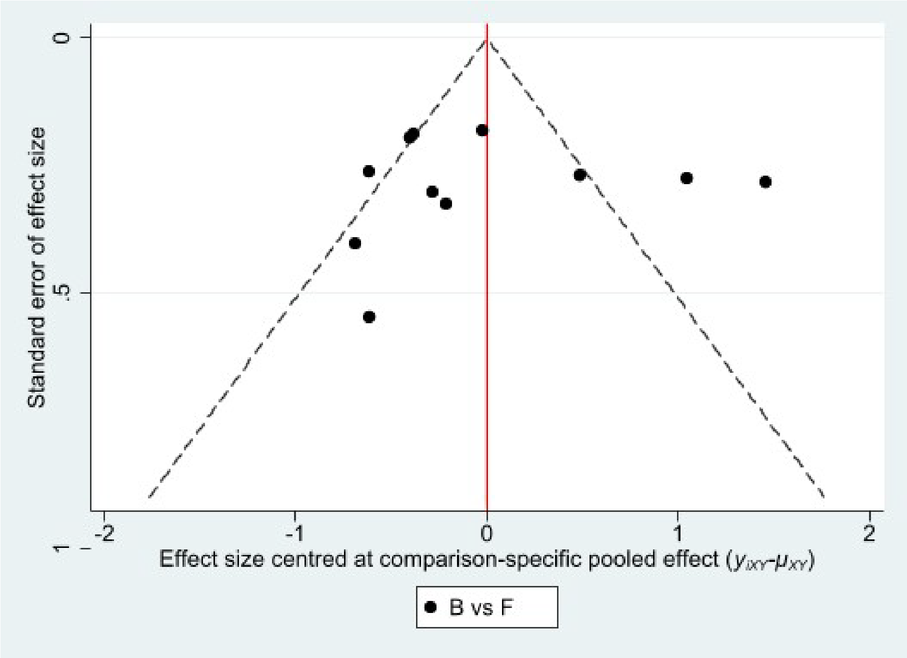

Egger's test for small-study effects:
Regress standard normal deviate of intervention
effect estimate against its standard error

```
Number of studies =  11                                  Root MSE      =   2.653
------------------------------------------------------------------------------
     Std_Eff |      Coef.   Std. Err.      t    P>|t|     [95% Conf. Interval]
-------------+----------------------------------------------------------------
       slope |  -.0807216   .7229205    -0.11   0.914    -1.716081    1.554638
        bias |   .7653089   2.834784     0.27   0.793    -5.647419    7.178037
------------------------------------------------------------------------------

Test of H0: no small-study effects          P = 0.793
```

GRADE appraisal (CINeMA)

The analysis of the certainty of the evidence was performed with the online application CINeMA, which follows the principles of the GRADE methodology. The following criteria were applied:

- Within-study bias: For each comparison, the histogram was interpreted according to a “Average risk of bias” rule;
- Across-studies bias was considered “undetected” when was not possible to evaluate the risk of publication bias;
- Imprecision: an effect size of 0.4 was considered as clinically important;
- Heterogeneity: an effect size of 0.4 was considered as clinically important;
- Incoherence: for all the comparisons for which only a direct or indirect estimation was available (Inconsistency measures: Not applicable) we reported “some concern”.

© 2023 American Medical Association. All rights reserved.

Final report

Comparison	N of studies	Within-study bias	Reporting bias	Indirectness	Imprecision	Heterogeneity	Incoherence	Confidence rating
3rd:cbt	11	Some concerns	Low risk	No concerns	No concerns	Major concerns	Major concerns	Very low
3rd:pe	1	Some concerns	Low risk	No concerns	Major concerns	No concerns	No concerns	Low
3rd:rt	2	Some concerns	Low risk	No concerns	Some concerns	Some concerns	No concerns	Low
3rd:sup	1	Some concerns	Low risk	No concerns	No concerns	Major concerns	No concerns	Low
3rd:tau	4	Some concerns	Low risk	No concerns	No concerns	Some concerns	No concerns	Moderate
3rd:wl	5	Some concerns	Low risk	No concerns	No concerns	No concerns	No concerns	Moderate
bt:cbt	2	Some concerns	Low risk	No concerns	Some concerns	Some concerns	No concerns	Low
bt:rt	1	Major concerns	Low risk	No concerns	Some concerns	Some concerns	No concerns	Low
bt:wl	3	Major concerns	Low risk	No concerns	No concerns	Major concerns	No concerns	Very low
cbt:cr	1	Some concerns	Low risk	No concerns	Some concerns	Some concerns	No concerns	Low
cbt:dyn	3	Major concerns	Low risk	No concerns	Some concerns	Some concerns	No concerns	Low
cbt:rt	8	Some concerns	Low risk	No concerns	Some concerns	Some concerns	No concerns	Low
cbt:sup	3	Major concerns	Low risk	No concerns	Some concerns	Some concerns	No concerns	Low
cbt:tau	8	Some concerns	Low risk	No concerns	No concerns	Some concerns	No concerns	Moderate
cbt:wl	30	Some concerns	Low risk	No concerns	No concerns	No concerns	No concerns	Moderate
cr:rt	1	Some concerns	Low risk	No concerns	Major concerns	No concerns	No concerns	Low
cr:sup	1	Some concerns	Low risk	No concerns	Major concerns	No concerns	No concerns	Low
cr:tau	1	Some concerns	Low risk	No concerns	Major concerns	No concerns	No concerns	Low
dyn:wl	1	Major concerns	Low risk	No concerns	Some concerns	Some concerns	No concerns	Low
rt:sup	1	Some concerns	Low risk	No concerns	Some concerns	Some concerns	No concerns	Low
rt:tau	1	Some concerns	Low risk	No concerns	No concerns	Major concerns	No concerns	Low
rt:wl	5	Some concerns	Low risk	No concerns	No concerns	Some concerns	No concerns	Moderate
sup:wl	1	Some concerns	Low risk	No concerns	No concerns	Major concerns	No concerns	Low
3rd:bt	0	Some concerns	Low risk	No concerns	Some concerns	Some concerns	No concerns	Low
3rd:cr	0	Some concerns	Low risk	No concerns	Some concerns	Some concerns	No concerns	Low
3rd:dyn	0	Some concerns	Low risk	No concerns	Some concerns	Some concerns	No concerns	Low
bt:cr	0	Some concerns	Low risk	No concerns	Major concerns	No concerns	No concerns	Low
bt:dyn	0	Major concerns	Low risk	No concerns	Major concerns	No concerns	No concerns	Very low
bt:pe	0	Some concerns	Low risk	No concerns	Major concerns	No concerns	No concerns	Low
bt:sup	0	Major concerns	Low risk	No concerns	Major concerns	No concerns	No concerns	Very low
bt:tau	0	Some concerns	Low risk	No concerns	Some concerns	Some concerns	No concerns	Low
cbt:pe	0	Some concerns	Low risk	No concerns	Major concerns	No concerns	No concerns	Low

© 2023 American Medical Association. All rights reserved.

cr:dyn	0	Some concerns	Low risk	No concerns	Major concerns	No concerns	No concerns	Low
cr:pe	0	Some concerns	Low risk	No concerns	Major concerns	No concerns	No concerns	Low
cr:wl	0	Some concerns	Low risk	No concerns	Some concerns	Some concerns	No concerns	Low
dyn:pe	0	Some concerns	Low risk	No concerns	Major concerns	No concerns	No concerns	Low
dyn:rt	0	Major concerns	Low risk	No concerns	Some concerns	Some concerns	No concerns	Low
dyn:sup	0	Major concerns	Low risk	No concerns	Major concerns	No concerns	No concerns	Very low
dyn:tau	0	Some concerns	Low risk	No concerns	Major concerns	No concerns	No concerns	Low
pe:rt	0	Some concerns	Low risk	No concerns	Major concerns	No concerns	No concerns	Low
pe:sup	0	Some concerns	Low risk	No concerns	Major concerns	No concerns	No concerns	Low
pe:tau	0	Some concerns	Low risk	No concerns	Major concerns	No concerns	No concerns	Low
pe:wl	0	Some concerns	Low risk	No concerns	Some concerns	Some concerns	No concerns	Low
sup:tau	0	Some concerns	Low risk	No concerns	Some concerns	Some concerns	No concerns	Low
tau:wl	0	Some concerns	Low risk	No concerns	Some concerns	Some concerns	No concerns	Low

© 2023 American Medical Association. All rights reserved.

eAppendix K. Primary outcome: acceptability

Treatment codes

WL	1	A
CBT	2	B
CR	3	C
BT	4	D
RT	5	E
3W	6	F
SP	7	G
PD	8	H
PE	9	I
TAU	10	J

BT=behaviour therapy; CBT=cognitive-behaviour therapy; CR=cognitive restructuring; PE=psychoeducation; RT=relaxation therapy; SP=supportive therapy; DYN= psychodynamic therapy; 3W=third-wave CBT; TAU=treatment as usual; WL=waiting list.

Network map

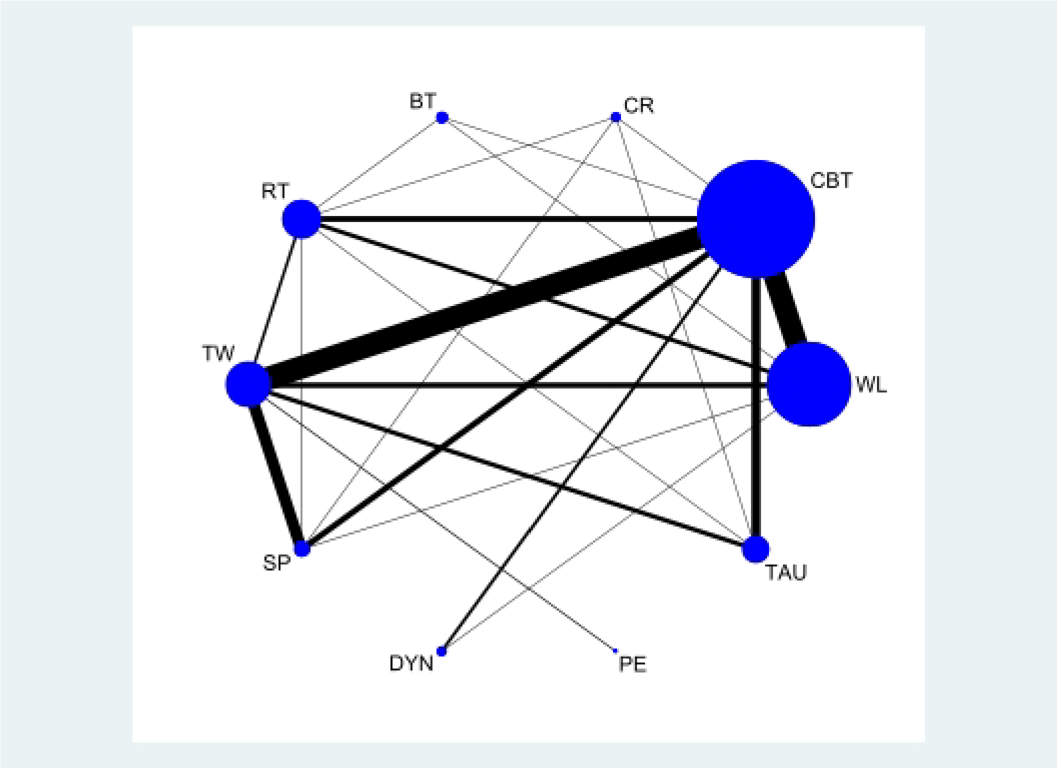

Pairwise meta-analysis

```
          Study       |      ES     [95% Conf. Interval]
----------------------+-----------------------------------------------------
     F - B
Afshari, 2020         |    0.667      0.119      3.741
```

© 2023 American Medical Association. All rights reserved.

Afshari, 2022	0.667	0.118	3.755
Avdagic, 2014	0.891	0.347	2.287
Jiang, 2022	0.883	0.568	1.372
Nordahl, 2018	0.879	0.164	4.718
Van der Heiden, 2012	1.027	0.568	1.858
Vera, 2021	1.000	0.432	2.315
Wetherell, 2011	3.636	0.484	27.331
Wong, 2016	0.857	0.306	2.403
Sub-total			
D+L pooled ES	0.936	0.708	1.239
J - B			
Alavi, 2020	0.500	0.097	2.577
Newman, 2020	0.636	0.269	1.508
Orvati Aziz, 2020	1.000	0.021	46.703
Stanley, 2009	3.063	1.169	8.024
Vera, 2021	0.375	0.110	1.279
Wong, 2016	1.743	0.737	4.124
Woodward, 1980	0.875	0.020	38.585
Sub-total			
D+L pooled ES	1.011	0.515	1.985
B - A			
Andersson, 2012	4.000	0.478	33.505
Barlow, 1992	0.345	0.139	0.857
Bowman, 1997	2.000	0.198	20.244
Butler, 1987	0.116	0.007	2.035
Butler, 1991	1.000	0.021	47.972
Carl, 2020	3.500	1.461	8.384
Cragan, 1984	1.050	0.070	15.676
Dugas, 2010	4.375	0.222	86.078
Dugas, 2003	1.080	0.164	7.099
Dugas, 2022	2.333	0.666	8.179
Hui, 2017	0.970	0.020	47.412
Jones, 2016	0.611	0.112	3.322
Ladouceur, 2000	0.867	0.018	40.684
Lindsay, 1987	0.333	0.015	7.323
Lorian, 2012	0.920	0.260	3.259
Mohlman, 2003	0.909	0.235	3.513
Nordahl, 2018	3.793	0.192	75.080
Paxling, 2011	6.136	0.770	48.913
Richards, 2016	0.752	0.368	1.538
Robinson, 2010	1.213	0.244	6.031
Rogiers, 2021	0.467	0.120	1.821
Stanley, 2003	2.628	0.898	7.689
Stanley, 2016	1.333	0.341	5.208
Titov, 2009	1.150	0.351	3.767
Van der Heiden, 2012	5.769	0.815	40.853
Wetherell, 2003	3.538	0.835	15.000
Zinbarg, 2007	3.300	0.151	72.084
Sub-total			
D+L pooled ES	1.316	0.925	1.872

© 2023 American Medical Association. All rights reserved.

H - A			
Andersson, 2012	1.000	0.066	15.180
Sub-total			
D+L pooled ES	1.000	0.066	15.180
H - B			
Andersson, 2012	0.250	0.030	2.094
Durham, 1994	2.844	1.146	7.062
Leichsenring, 2009	1.554	0.280	8.608
Sub-total			
D+L pooled ES	1.356	0.373	4.933
E - B			
Arntz, 2003	0.625	0.127	3.072
Barlow, 1992	2.175	0.786	6.019
Borkovec, 1993	1.250	0.384	4.072
Cragan, 1984	4.000	0.489	32.724
Dugas, 2010	2.296	0.577	9.137
Lindsay, 1987	1.000	0.022	46.053
Ost, 2000	2.500	0.248	25.153
Woodward, 1980	0.875	0.020	38.585
Sub-total			
D+L pooled ES	1.681	0.957	2.951
G - C			
Artiran, 2022	1.500	0.284	7.934
Sub-total			
D+L pooled ES	1.500	0.284	7.934
E - A			
Barlow, 1992	0.750	0.347	1.619
Cragan, 1984	4.200	0.512	34.435
Dugas, 2010	10.043	0.590	170.869
Hoyer, 2009	3.444	0.335	35.371
Lindsay, 1987	0.333	0.015	7.323
Sub-total			
D+L pooled ES	1.644	0.544	4.962
G - B			
Borkovec, 1993	0.575	0.117	2.814
Stanley, 1996	1.138	0.496	2.609
Wetherell, 2003	1.000	0.442	2.260
Sub-total			
D+L pooled ES	0.990	0.574	1.710
G - E			
Borkovec, 1993	0.460	0.100	2.117
Sub-total			
D+L pooled ES	0.460	0.100	2.117
D - A			
Butler, 1991	3.000	0.130	69.313
Hoyer, 2009	5.167	0.617	43.283

© 2023 American Medical Association. All rights reserved.

```
Pallavicini, 2009    |    1.667     0.082    33.749
 Sub-total           |
  D+L pooled ES      |    3.409     0.746    15.574
---------------------+--------------------------------------------------
     D - B
Butler, 1991         |    3.000     0.130    69.313
 Sub-total           |
  D+L pooled ES      |    3.000     0.130    69.313
---------------------+--------------------------------------------------
     F - A
Dahlin, 2016         |    1.226     0.526     2.856
Mennin, 2018         |    0.536     0.142     2.017
Nordahl, 2018        |    3.333     0.168    66.157
Roemer, 2008         |    0.533     0.114     2.499
Van der Heiden, 2012 |    5.926     0.840    41.821
 Sub-total           |
  D+L pooled ES      |    1.126     0.524     2.422
---------------------+--------------------------------------------------
     G - F
de Almeida Sampaio,  |    0.920     0.622     1.362
 Sub-total           |
  D+L pooled ES      |    0.920     0.622     1.362
---------------------+--------------------------------------------------
     F - E
Hayes-Skelton, 2013  |    1.281     0.564     2.913
Wells, 2010          |    0.333     0.015     7.323
 Sub-total           |
  D+L pooled ES      |    1.172     0.530     2.593
---------------------+--------------------------------------------------
     I - F
Hoge, 2013           |    3.911     1.166    13.117
 Sub-total           |
  D+L pooled ES      |    3.911     1.166    13.117
---------------------+--------------------------------------------------
     E - D
Hoyer, 2009          |    0.667     0.137     3.248
 Sub-total           |
  D+L pooled ES      |    0.667     0.137     3.248
---------------------+--------------------------------------------------
     J - F
Roy, 2021            |    0.108     0.006     1.925
Vera, 2021           |    0.375     0.110     1.279
Wong, 2016           |    2.033     0.816     5.066
Zargar, 2012         |    1.000     0.250     3.998
 Sub-total           |
  D+L pooled ES      |    0.761     0.261     2.219
---------------------+--------------------------------------------------
     G - A
Wetherell, 2003      |    3.538     0.835    15.000
 Sub-total           |
  D+L pooled ES      |    3.538     0.835    15.000
---------------------+--------------------------------------------------
     C - B
```

© 2023 American Medical Association. All rights reserved.

```
Woodward, 1980       |     0.875     0.020    38.585
 Sub-total           |
  D+L pooled ES      |     0.875     0.020    38.585
---------------------+---------------------------------------------------
     E - C
Woodward, 1980       |     1.000     0.022    44.498
 Sub-total           |
  D+L pooled ES      |     1.000     0.022    44.498
---------------------+---------------------------------------------------
     J - C
Woodward, 1980       |     1.000     0.022    44.498
 Sub-total           |
  D+L pooled ES      |     1.000     0.022    44.498
---------------------+---------------------------------------------------
     J - E
Woodward, 1980       |     1.000     0.022    44.498
 Sub-total           |
  D+L pooled ES      |     1.000     0.022    44.498
---------------------+---------------------------------------------------

Test(s) of heterogeneity:
               Heterogeneity  degrees of
                 statistic     freedom      P    I-squared**   Tau-
squared

F - B               2.27          8      0.972       0.0%       0.0000
J - B              10.84          6      0.093      44.7%       0.3270
B - A              33.87         26      0.138      23.2%       0.1806
H - A               0.00          0        .          .%        0.0000
H - B               4.31          2      0.116      53.5%       0.7035
E - B               3.12          7      0.874       0.0%       0.0000
G - C               0.00          0        .          .%        0.0000
E - A               6.34          4      0.175      37.0%       0.5782
G - B               0.56          2      0.757       0.0%       0.0000
G - E               0.00          0        .          .%        0.0000
D - A               0.37          2      0.831       0.0%       0.0000
D - B               0.00          0        .          .%        0.0000
F - A               5.42          4      0.247      26.2%       0.2008
G - F               0.00          0        .          .%        0.0000
F - E               0.68          1      0.409       0.0%       0.0000
I - F               0.00          0        .          .%        0.0000
E - D               0.00          0        .          .%        0.0000
J - F               7.06          3      0.070      57.5%       0.6448
G - A               0.00          0        .          .%        0.0000
C - B               0.00          0        .          .%        0.0000
E - C               0.00          0        .          .%        0.0000
J - C               0.00          0        .          .%        0.0000
J - E               0.00          0        .          .%        0.0000
** I-squared: the variation in ES attributable to heterogeneity)
```

© 2023 American Medical Association. All rights reserved.

```
Significance test(s) of ES=1

F - B                  z=  0.46     p = 0.645
J - B                  z=  0.03     p = 0.974
B - A                  z=  1.52     p = 0.127
H - A                  z=  0.00     p = 1.000
H - B                  z=  0.46     p = 0.644
E - B                  z=  1.81     p = 0.071
G - C                  z=  0.48     p = 0.633
E - A                  z=  0.88     p = 0.378
G - B                  z=  0.03     p = 0.972
G - E                  z=  1.00     p = 0.319
D - A                  z=  1.58     p = 0.114
D - B                  z=  0.69     p = 0.493
F - A                  z=  0.30     p = 0.761
G - F                  z=  0.42     p = 0.677
F - E                  z=  0.39     p = 0.695
I - F                  z=  2.21     p = 0.027
E - D                  z=  0.50     p = 0.616
J - F                  z=  0.50     p = 0.617
G - A                  z=  1.71     p = 0.086
C - B                  z=  0.07     p = 0.945
E - C                  z=  0.00     p = 1.000
J - C                  z=  0.00     p = 1.000
J - E                  z=  0.00     p = 1.000
-------------------------------------------------------------------
```

Interval plot

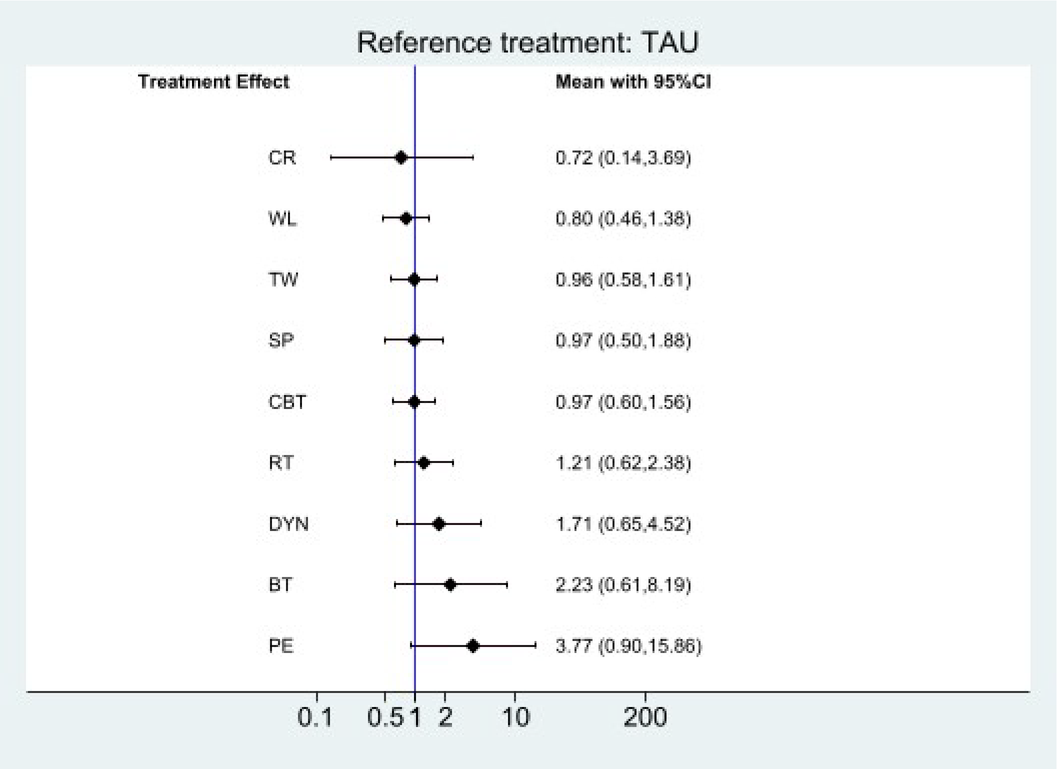

© 2023 American Medical Association. All rights reserved.

BT=behaviour therapy; CBT=cognitive-behaviour therapy; CR=cognitive restructuring; PE=psychoeducation; RT=relaxation therapy; SP=supportive therapy; DYN= psychodynamic therapy; 3W=third-wave CBT; TAU=treatment as usual; WL=waiting list.

Net league table

BT	0.43 (0.13,1.46)	0.32 (0.04,2.34)	0.77 (0.17,3.41)	1.69 (0.27,10.50)	0.54 (0.16,1.84)	0.43 (0.12,1.58)	0.43 (0.13,1.49)	0.45 (0.12,1.65)	0.36 (0.11,1.20)
2.31 (0.68,7.77)	CBT	0.74 (0.15,3.62)	1.77 (0.75,4.18)	3.91 (0.99,15.46)	1.25 (0.77,2.03)	1.00 (0.61,1.63)	1.00 (0.74,1.35)	1.04 (0.64,1.67)	0.83 (0.62,1.11)
3.11 (0.43,22.60)	1.35 (0.28,6.57)	CR	2.38 (0.39,14.50)	5.26 (0.66,42.12)	1.69 (0.33,8.61)	1.35 (0.29,6.29)	1.35 (0.27,6.59)	1.40 (0.27,7.18)	1.12 (0.22,5.55)
1.30 (0.29,5.78)	0.57 (0.24,1.33)	0.42 (0.07,2.55)	DYN	2.21 (0.44,11.18)	0.71 (0.26,1.92)	0.56 (0.21,1.52)	0.56 (0.23,1.41)	0.59 (0.22,1.55)	0.47 (0.19,1.15)
0.59 (0.10,3.66)	0.26 (0.06,1.01)	0.19 (0.02,1.52)	0.45 (0.09,2.29)	PE	0.32 (0.08,1.35)	0.26 (0.06,1.07)	0.26 (0.07,0.98)	0.27 (0.06,1.11)	0.21 (0.05,0.85)
1.84 (0.54,6.23)	0.80 (0.49,1.29)	0.59 (0.12,3.02)	1.41 (0.52,3.83)	3.12 (0.74,13.13)	RT	0.80 (0.42,1.51)	0.80 (0.48,1.34)	0.83 (0.42,1.62)	0.66 (0.39,1.11)
2.31 (0.63,8.42)	1.00 (0.61,1.63)	0.74 (0.16,3.47)	1.77 (0.66,4.78)	3.91 (0.94,16.32)	1.25 (0.66,2.38)	SP	1.00 (0.61,1.64)	1.04 (0.53,2.01)	0.83 (0.48,1.43)
2.31 (0.67,7.94)	1.00 (0.74,1.36)	0.74 (0.15,3.64)	1.77 (0.71,4.41)	3.91 (1.02,14.96)	1.25 (0.75,2.10)	1.00 (0.61,1.64)	TW	1.04 (0.62,1.73)	0.83 (0.57,1.21)
2.23 (0.61,8.19)	0.97 (0.60,1.56)	0.72 (0.14,3.69)	1.71 (0.65,4.52)	3.77 (0.90,15.86)	1.21 (0.62,2.38)	0.97 (0.50,1.88)	0.96 (0.58,1.61)	TAU	0.80 (0.46,1.38)
2.79 (0.83,9.33)	1.21 (0.90,1.63)	0.90 (0.18,4.46)	2.14 (0.87,5.27)	4.72 (1.17,19.02)	1.51 (0.90,2.54)	1.21 (0.70,2.09)	1.21 (0.83,1.76)	1.25 (0.72,2.16)	WL

Net league table: head-to-head comparisons. Effect sizes (ES) and 95% confidence intervals (CIs) are reported. Negative ES favour the column-defining treatment. Statistically significant results are highlighted in boldface. BT=behaviour therapy; CBT=cognitive-behaviour therapy; CR=cognitive restructuring; PE=psychoeducation; RT=relaxation therapy; SP=supportive therapy; DYN= psychodynamic therapy; 3W=third-wave CBT; TAU=treatment as usual; WL=waiting list.

Evaluation of heterogeneity and incoherence

Overall heterogeneity

SD=0.29; Restricted likelihood ratio test for heterogeneity: LRT = 1.53 (d.f. = 1) P = 0.108

Overall incoherence

chi2(45) = 16.81

Prob > chi2 = 0.8183

© 2023 American Medical Association. All rights reserved.

Loop-specific approach

* 22 triangular loops found
* 1 quadratic loops found

Note: Heterogeneity of loop C-E-G cannot be estimated due to insufficient observations - set equal to 0

Note: Heterogeneity of loop C-E-J cannot be estimated due to insufficient observations - set equal to 0

Note: Heterogeneity of loop E-F-G cannot be estimated due to insufficient observations - set equal to 0

Evaluation of inconsistency using loop-specific heterogeneity estimates:

Loop	ROR	z_value	p_value	CI_95	Loop_Heterog_tau2
A-E-G	4.680	0.953	0.341	(1.00,111.96)	0.578
A-F-G	3.415	1.109	0.267	(1.00,29.90)	0.201
C-E-G	3.261	0.524	0.600	(1.00,270.15)	0.000
A-B-G	2.944	1.206	0.228	(1.00,17.03)	0.109
E-F-G	2.345	0.946	0.344	(1.00,13.69)	0.000
A-B-E	2.202	1.673	0.094	(1.00,5.55)	0.005
B-E-F	2.104	1.440	0.150	(1.00,5.79)	0.000
A-B-H	2.039	0.458	0.647	(1.00,43.20)	0.218
B-C-E	1.949	0.243	0.808	(1.00,427.84)	0.000
A-D-E	1.870	0.477	0.633	(1.00,24.47)	0.181
B-E-J	1.600	0.235	0.814	(1.00,80.42)	0.076
A-E-F	1.356	0.338	0.735	(1.00,7.94)	0.290
B-C-G	1.325	0.132	0.895	(1.00,85.98)	0.000
E-F-J	1.294	0.114	0.909	(1.00,109.12)	0.531
C-F-G-J	1.241	0.083	0.934	(1.00,206.57)	0.645
B-F-J	1.233	0.401	0.688	(1.00,3.44)	0.108
B-D-E	1.190	0.096	0.924	(1.00,41.91)	0.000
B-E-G	1.174	0.181	0.857	(1.00,6.71)	0.000
A-B-D	1.173	0.086	0.931	(1.00,44.09)	0.163
B-C-J	1.151	0.048	0.961	(1.00,339.92)	0.402
B-F-G	1.150	0.375	0.707	(1.00,2.38)	0.000
A-B-F	1.037	0.093	0.926	(1.00,2.25)	0.030
C-E-J	.	.	.		0.000

*** Note: Loop C-E-J is formed only by multi-arm trial(s) - Consistent by definition

© 2023 American Medical Association. All rights reserved.

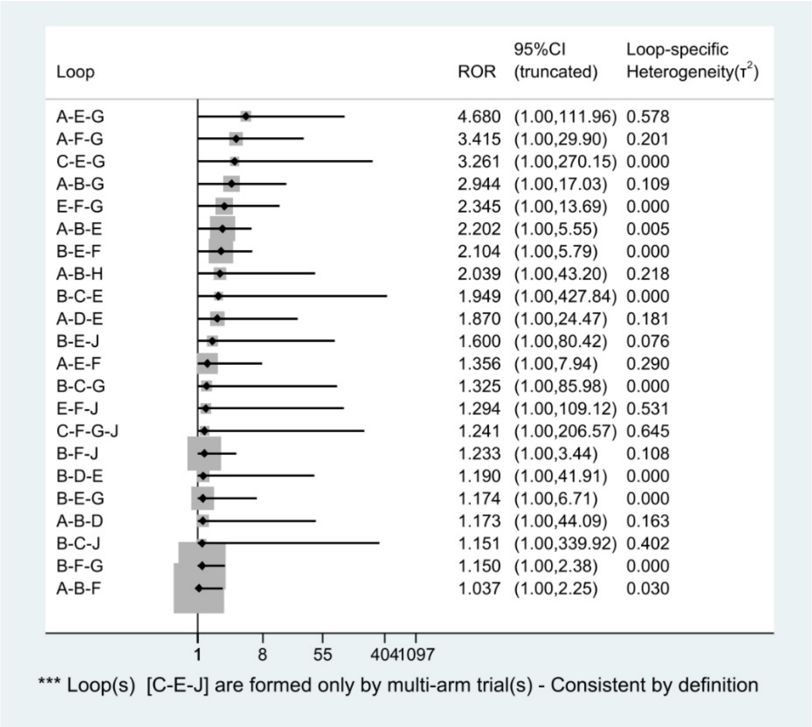

Consistency between direct and indirect estimates

Side	Direct Coef.	Std. Err.	Indirect Coef.	Std. Err.	Difference Coef.	Std. Err.	P>|z|
A B	.2232443	.1652651	.0088512	.3750929	.214393	.4032806	0.595
A D *	1.212954	.796009	.4719717	1.606592	.7409826	1.987213	0.709
A E	.2321649	.3957669	.5661743	.3508242	-.3340094	.5280518	0.527
A F	.0714339	.3338618	.2555202	.2465364	-.1840863	.4177976	0.659
A G	.9637637	.7316826	.0256053	.3151264	.9381584	.8186519	0.252
A H	4.52e-12	1.419142	.8479534	.4853898	-.8479534	1.499856	0.572
B C	-.1352021	1.954653	-.3314229	.887911	.1962208	2.146005	0.927
B D	1.090429	1.580054	.7824991	.6908761	.3079295	1.756613	0.861
B E	.5190512	.3034878	-.2737368	.3862487	.792788	.5004271	0.113
B F	-.051481	.1975041	.0903794	.2643136	-.1418604	.3298432	0.667
B G	-.0232775	.3450128	.0368394	.3911822	-.0601169	.5185882	0.908
B H *	.5462231	.440128	2.186775	2.24075	-1.640552	2.229242	0.462
B J	.0832448	.2705095	-.2020588	.554993	.2853036	.6077103	0.639
C E	4.16e-11	1.95947	.6395315	.9199872	-.6395315	2.164694	0.768
C G	.4054653	.9007976	-.0511358	1.620872	.4566011	1.854387	0.806
C J	-4.29e-13	1.959227	.405925	.9242492	-.405925	2.166289	0.851
D E	-.4691693	.8559689	-.7808822	.9552517	.3117129	1.310847	0.812
E F	.1210223	.493827	-.3651138	.3103499	.4861361	.580673	0.402
E G	-.6832824	.8064454	-.1374352	.3581166	-.5458471	.8822835	0.536
E J	-5.72e-12	1.959324	-.1976942	.350759	.1976942	1.990473	0.921
F G	-.0833809	.3821404	.0825725	.3642982	-.1659534	.5279624	0.753
F I *	1.363822	.6844725	.987705	199.9999	.3761165	199.9992	0.998
F J	-.0435436	.3701431	.1073157	.3751121	-.1508593	.5223758	0.773

SUCRA and cumulative probability plots

Estimated probabilities (%) of each treatment being the best (and other ranks)
- assuming the minimum parameter is the best
- using 5000 draws
- allowing for parameter uncertainty

id_study and Rank	Treatment 1	2	3	4	5	6	7	8	9	10
1										
Best	24.7	1.0	51.1	2.5	1.3	2.9	6.4	1.8	0.7	7.7

© 2023 American Medical Association. All rights reserved.

2nd	36.1	7.2	6.8	2.6	3.6	9.6	16.3	2.9	0.8	14.0
3rd	19.9	19.2	4.1	1.7	5.4	18.7	15.2	2.2	0.4	13.1
4th	10.8	26.0	3.9	1.8	6.4	22.2	12.9	2.6	0.6	12.8
5th	5.0	25.1	3.4	2.5	9.8	20.7	15.5	3.5	0.7	13.9
6th	2.6	15.3	5.3	3.3	18.0	16.3	15.9	6.6	1.5	15.4
7th	0.7	5.0	7.4	7.5	30.3	7.7	11.8	12.7	2.4	14.5
8th	0.2	1.1	9.4	17.6	19.9	1.8	4.9	30.9	7.3	6.8
9th	0.0	0.1	6.3	37.0	5.0	0.2	1.0	28.5	20.3	1.7
Worst	0.0	0.0	2.4	23.5	0.3	0.0	0.0	8.2	65.3	0.1
MEAN RANK	2.5	4.4	3.5	8.1	6.3	4.4	4.4	7.6	9.3	4.6
SUCRA	0.8	0.6	0.7	0.2	0.4	0.6	0.6	0.3	0.1	0.6

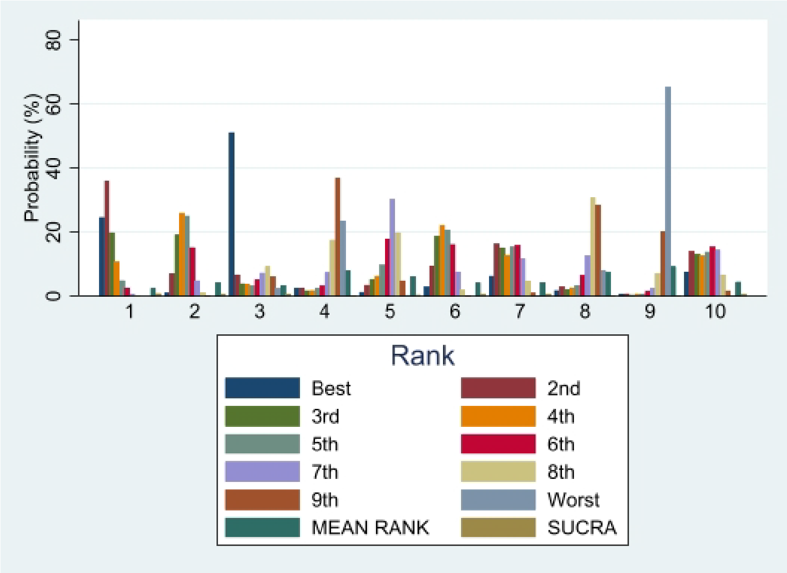

Treatment Relative Ranking of Model 1

Treatm~t	SUCRA	PrBest	MeanRank
WL	83.7	24.7	2.5
CBT	62.4	1.0	4.4
CR	72.4	51.1	3.5
BT	20.7	2.5	8.1
RT	41.5	1.3	6.3
TW	62.5	2.9	4.4
SP	62.2	6.4	4.4
DYN	27.0	1.8	7.6
PE	7.8	0.7	9.3
TAU	60.0	7.7	4.6

© 2023 American Medical Association. All rights reserved.

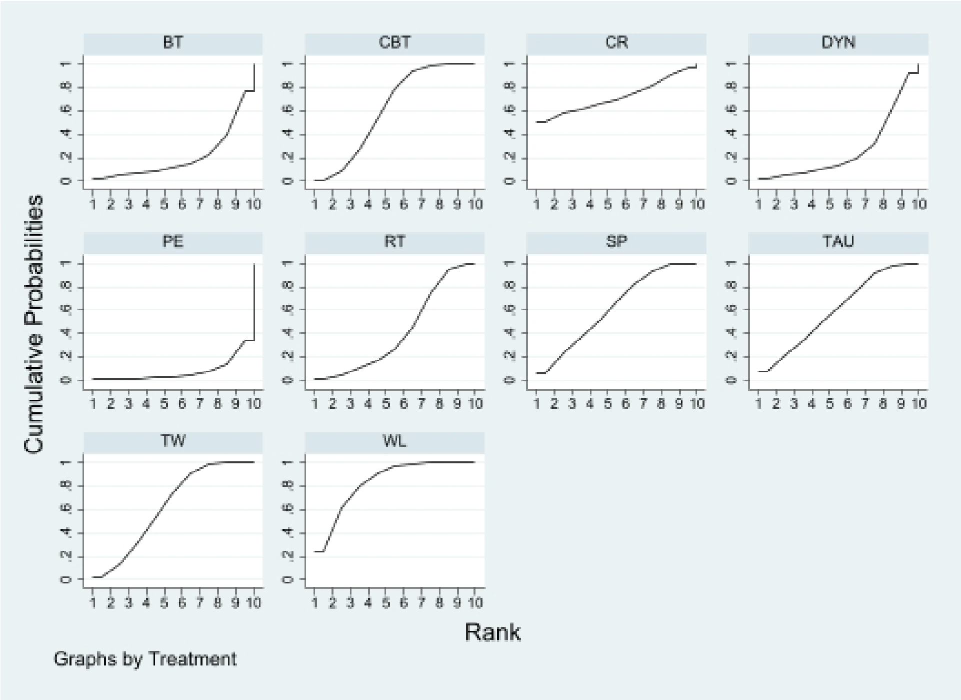

Funnel plot

2 comparisons with 10 one more studies: CBT-WL (30 studies); 3W-CBT (11 studies);

1) CBT-WL (27 studies)

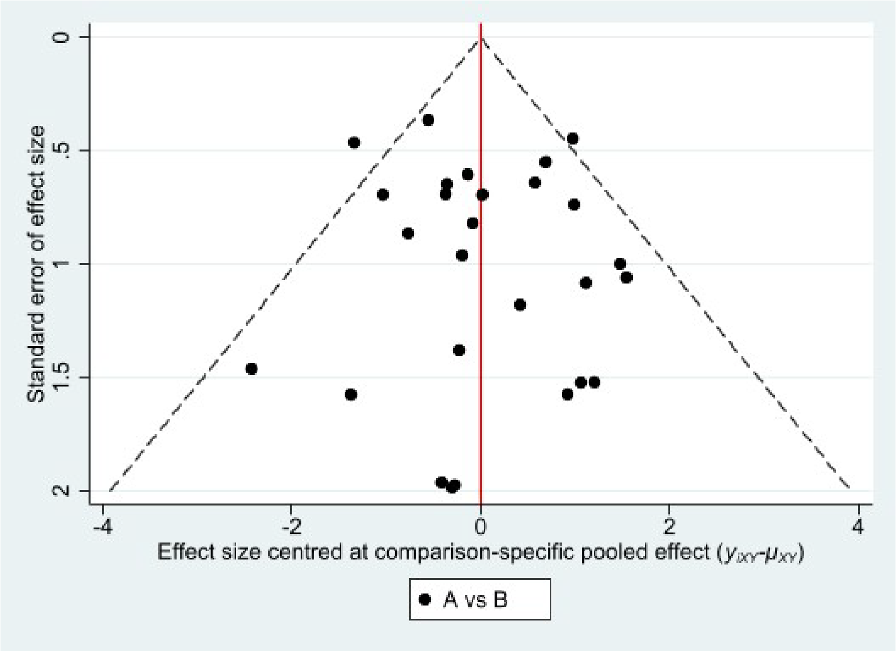

Egger's test for small-study effects:
Regress standard normal deviate of intervention
effect estimate against its standard error

© 2023 American Medical Association. All rights reserved.

```
Number of studies =  27                                 Root MSE      =   1.152
------------------------------------------------------------------------------
     Std_Eff |      Coef.   Std. Err.      t    P>|t|     [95% Conf. Interval]
-------------+----------------------------------------------------------------
       slope |  -.0215158   .3857815    -0.06   0.956    -.8160477    .7730162
        bias |   .3692352   .5089151     0.73   0.475     -.678895    1.417365
------------------------------------------------------------------------------

Test of H0: no small-study effects          P = 0.475
```

GRADE appraisal (CINeMA)

- Within-study bias: the "overall" risk of bias of each study was calculated as follows: (a) LOW risk if there "some concerns" on max two domains of the Cochrane RoB 2; (b) SOME CONCERNS if three domains were judged as having some concerns OR one domain was considered at high risk and two leaving some concerns; (c) HIGH RISK in all other cases (two or more high risk domains; three domains leaving some concerns + one high risk domain; 4 or more domains leaving some concerns).
  For each comparison, the histogram was interpreted according to a "Average risk of bias" rule;
- Across-studies bias was considered "undetected" when was not possible to evaluate the risk of publication bias;
- Imprecision: risk ratio between 0.667 to 1.5 was considered as a clinically important size of effect;
- Heterogeneity: risk ratio between 0.667 to 1.5 was considered as a clinically important size of effect;
- Incoherence: for all the comparisons for which only a direct or indirect estimation was available (Inconsistency measures: Not applicable) we reported "some concern".

Final report

Comparison	N of studies	Within-study bias	Reporting bias	Indirectness	Imprecision	Heterogeneity	Incoherence	Confidence rating
3rd:cbt	9	Some concerns	Low risk	No concerns	No concerns	No concerns	No concerns	Moderate
3rd:pe	1	Some concerns	Low risk	No concerns	No concerns	No concerns	No concerns	Moderate
3rd:rt	2	Some concerns	Low risk	No concerns	Some concerns	No concerns	No concerns	Moderate
3rd:sup	1	Major concerns	Low risk	No concerns	No concerns	Major concerns	No concerns	Very low
3rd:tau	4	Some concerns	Low risk	No concerns	Some concerns	No concerns	No concerns	Moderate
3rd:wl	5	Some concerns	Low risk	No concerns	Some concerns	No concerns	No concerns	Moderate
bt:cbt	1	Some concerns	Low risk	No concerns	Some concerns	Some concerns	No concerns	Low
bt:rt	1	Major concerns	Low risk	No concerns	Major concerns	No concerns	No concerns	Very low
bt:wl	3	Major concerns	Low risk	No concerns	Some concerns	No concerns	No concerns	Low
cbt:cr	1	Some concerns	Low risk	No concerns	Major concerns	No concerns	No concerns	Low
cbt:dyn	3	Major concerns	Low risk	No concerns	Some concerns	No concerns	No concerns	Low
cbt:rt	8	Some concerns	Low risk	No concerns	Some concerns	No concerns	Major concerns	Low
cbt:sup	3	Major concerns	Low risk	No concerns	Some concerns	Some concerns	No concerns	Low
cbt:tau	7	Some concerns	Low risk	No concerns	Some concerns	No concerns	No concerns	Moderate

© 2023 American Medical Association. All rights reserved.

cbt:wl	27	Some concerns	Low risk	No concerns	Some concerns	No concerns	No concerns	Moderate
cr:rt	1	Some concerns	Low risk	No concerns	Major concerns	No concerns	No concerns	Low
cr:sup	1	Some concerns	Low risk	No concerns	Major concerns	No concerns	No concerns	Low
cr:tau	1	Some concerns	Low risk	No concerns	Major concerns	No concerns	No concerns	Low
dyn:wl	1	Some concerns	Low risk	No concerns	Some concerns	No concerns	No concerns	Moderate
rt:sup	1	Major concerns	Low risk	No concerns	Some concerns	Some concerns	No concerns	Low
rt:tau	1	Some concerns	Low risk	No concerns	Major concerns	No concerns	No concerns	Low
rt:wl	5	Major concerns	Low risk	No concerns	Some concerns	No concerns	No concerns	Low
sup:wl	1	Some concerns	Low risk	No concerns	Some concerns	No concerns	No concerns	Moderate
3rd:bt	0	Some concerns	Low risk	No concerns	Some concerns	Some concerns	No concerns	Low
3rd:cr	0	Some concerns	Low risk	No concerns	Major concerns	No concerns	No concerns	Low
3rd:dyn	0	Some concerns	Low risk	No concerns	Some concerns	No concerns	No concerns	Moderate
bt:cr	0	Some concerns	Low risk	No concerns	Major concerns	No concerns	No concerns	Low
bt:dyn	0	Major concerns	Low risk	No concerns	Major concerns	No concerns	No concerns	Very low
bt:pe	0	Some concerns	Low risk	No concerns	Major concerns	No concerns	No concerns	Low
bt:sup	0	Major concerns	Low risk	No concerns	Some concerns	Some concerns	No concerns	Low
bt:tau	0	Some concerns	Low risk	No concerns	Major concerns	No concerns	No concerns	Low
cbt:pe	0	Some concerns	Low risk	No concerns	No concerns	No concerns	No concerns	Moderate
cr:dyn	0	Some concerns	Low risk	No concerns	Major concerns	No concerns	No concerns	Low
cr:pe	0	Some concerns	Low risk	No concerns	Some concerns	No concerns	No concerns	Moderate
cr:wl	0	Some concerns	Low risk	No concerns	Major concerns	No concerns	No concerns	Low
dyn:pe	0	Some concerns	Low risk	No concerns	Major concerns	No concerns	No concerns	Low
dyn:rt	0	Major concerns	Low risk	No concerns	Major concerns	No concerns	No concerns	Very low
dyn:sup	0	Major concerns	Low risk	No concerns	Some concerns	No concerns	No concerns	Low
dyn:tau	0	Some concerns	Low risk	No concerns	Some concerns	No concerns	No concerns	Moderate
pe:rt	0	Some concerns	Low risk	No concerns	Some concerns	No concerns	No concerns	Moderate
pe:sup	0	Some concerns	Low risk	No concerns	No concerns	No concerns	No concerns	Moderate
pe:tau	0	Some concerns	Low risk	No concerns	Some concerns	No concerns	No concerns	Moderate
pe:wl	0	Some concerns	Low risk	No concerns	No concerns	No concerns	No concerns	Moderate
sup:tau	0	Some concerns	Low risk	No concerns	Some concerns	Some concerns	No concerns	Low
tau:wl	0	Some concerns	Low risk	No concerns	Some concerns	No concerns	No concerns	Moderate

© 2023 American Medical Association. All rights reserved.

eAppendix L. Secondary outcome: efficacy at follow-up

Analysis	Network plot & Forest plot	Overall heterogeneity
Anxiety severity at 3 to 12 months of follow-up after completion of the intervention.	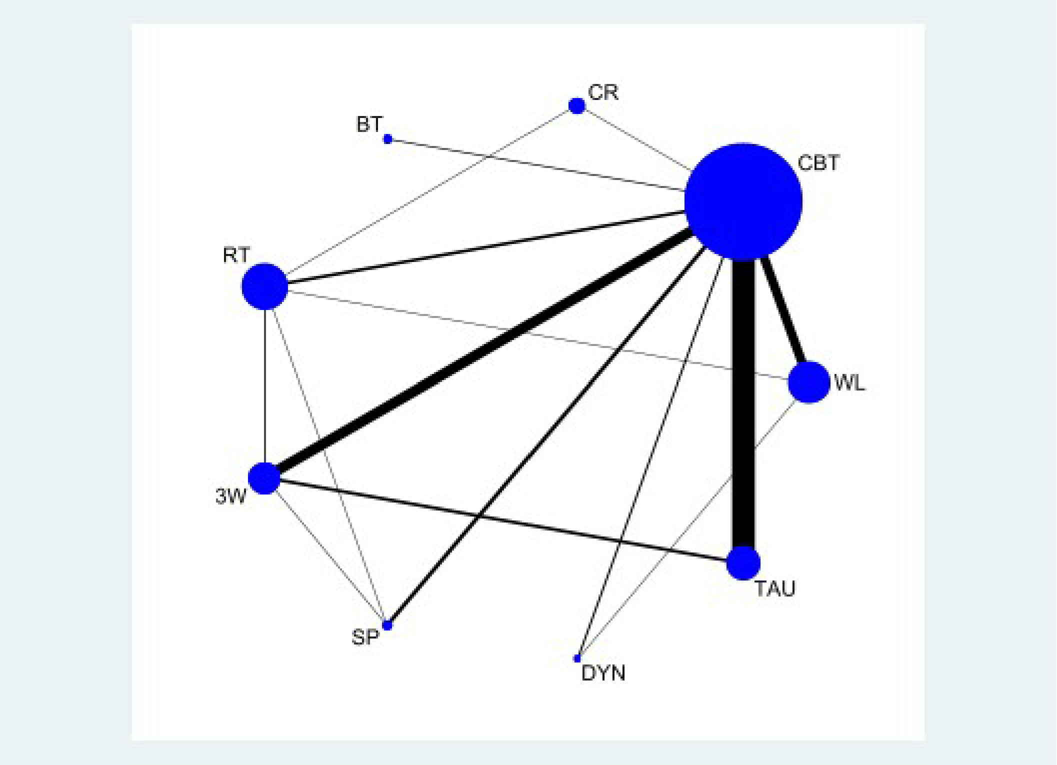<br>BT=behaviour therapy; CBT=cognitive-behaviour therapy; CR=cognitive restructuring; RT=relaxation therapy; SP=supportive therapy; DYN= psychodynamic therapy; 3W=third-wave CBT; TAU=treatment as usual; WL=waiting list.	SD=0.45; Restricted likelihood ratio test for heterogeneity: LRT = 35.37 (d.f. = 1) P = 0.000<br>**Overall incoherence**<br>chi2(11) = 5.05<br>Prob > chi2 = 0.9289<br>**Loop-specific approach**<br>No loop (0/8 loops) showed inconsistency<br>**Consistency between direct and indirect estimates**<br>No inconsistencies

© 2023 American Medical Association. All rights reserved.

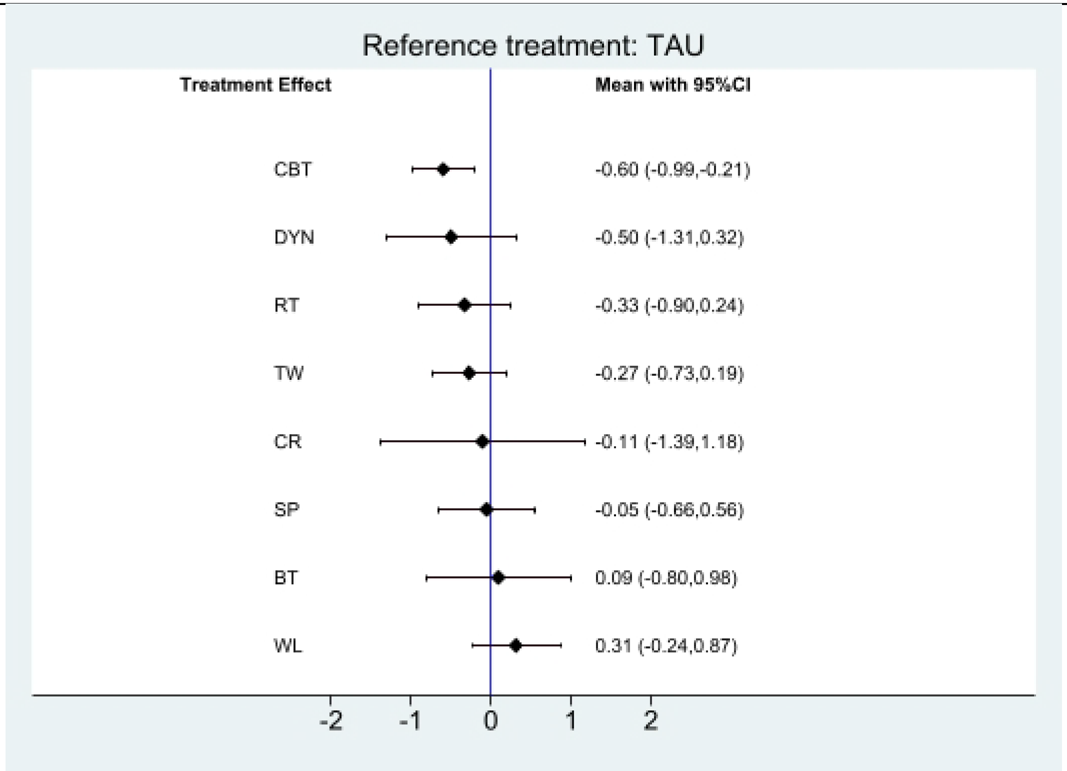

BT=behaviour therapy; CBT=cognitive-behaviour therapy; CR=cognitive restructuring; RT=relaxation therapy; SP=supportive therapy; DYN= psychodynamic therapy; 3W=third-wave CBT; TAU=treatment as usual; WL=waiting list.

© 2023 American Medical Association. All rights reserved.

eAppendix M. Sensitivity analyses (prespecified)

Sensitivity 1

Excerpt from the protocol: “We will examine the impact of studies that included participants without formal diagnosis of generalized anxiety disorder and studies that used DSM-III criteria to establish the diagnosis of generalized anxiety disorder by excluding such studies from the analysis for the primary outcome on efficacy.”

Analysis	Forest plot	Overall heterogeneity
Excluding studies that included participants without formal diagnosis of generalized anxiety disorder and studies that used DSM-III criteria to establish the diagnosis of generalized anxiety disorder*	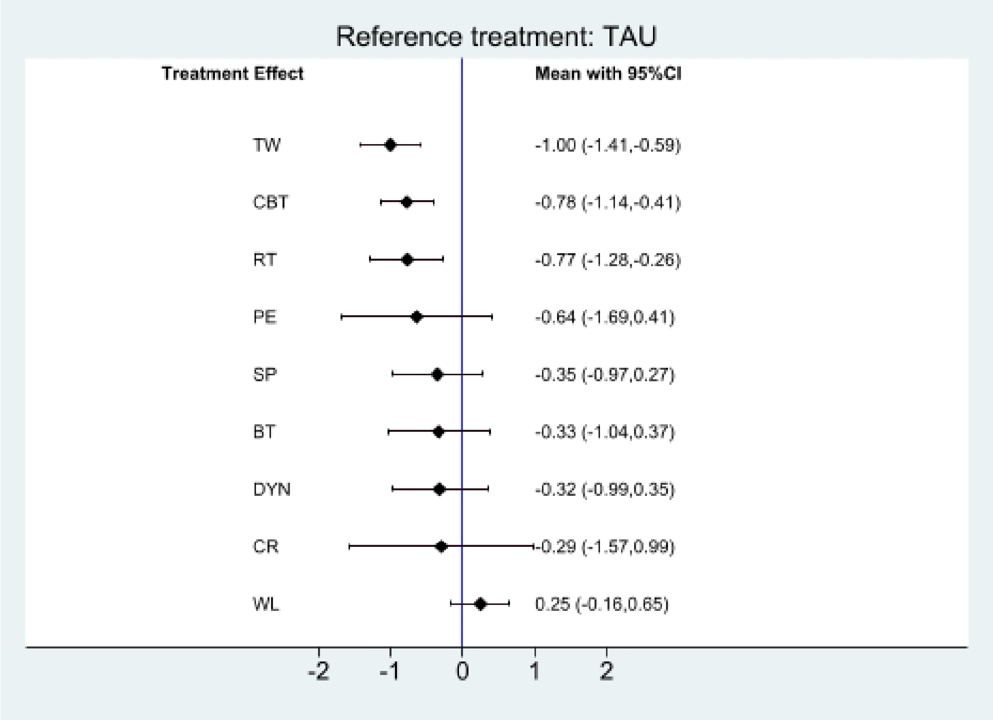 BT=behaviour therapy; CBT=cognitive-behaviour therapy; CR=cognitive restructuring; PE=psychoeducation; RT=relaxation therapy; SP=supportive therapy; DYN= psychodynamic therapy; 3W=third-wave CBT; TAU=treatment as usual; WL=waiting list.	SD=0.44; Restricted likelihood ratio test for heterogeneity: LRT = 54.73 (d.f. = 1) P = 0.000<br>**Overall incoherence**<br>chi2(20) = 10.27<br>Prob > chi2 = 0.9631<br>**Loop-specific approach**<br>1 (A-D-E) out of 15 loops showed inconsistency<br>**Consistency between direct and indirect estimates**<br>No inconsistencies

*Afshari 2020, Afshari 2022, Butler 1987, Cragan 1984, Durham 1987, Linsday 1987, Richards 2016, Vera 2021, Woodward 1980

© 2023 American Medical Association. All rights reserved.

Sensitivity 2

Excerpt from the protocol: “A further sensitivity analysis will be conducted to test whether the results could be influenced by the type of outcome hierarchy (Appendix D). In this sensitivity, measures of “worry” will be considered at the top of the hierarchy instead of being considered at the bottom.”

Analysis	Forest plot	Overall heterogeneity
Measures of “worry” considered at the top of the outcome hierarchy	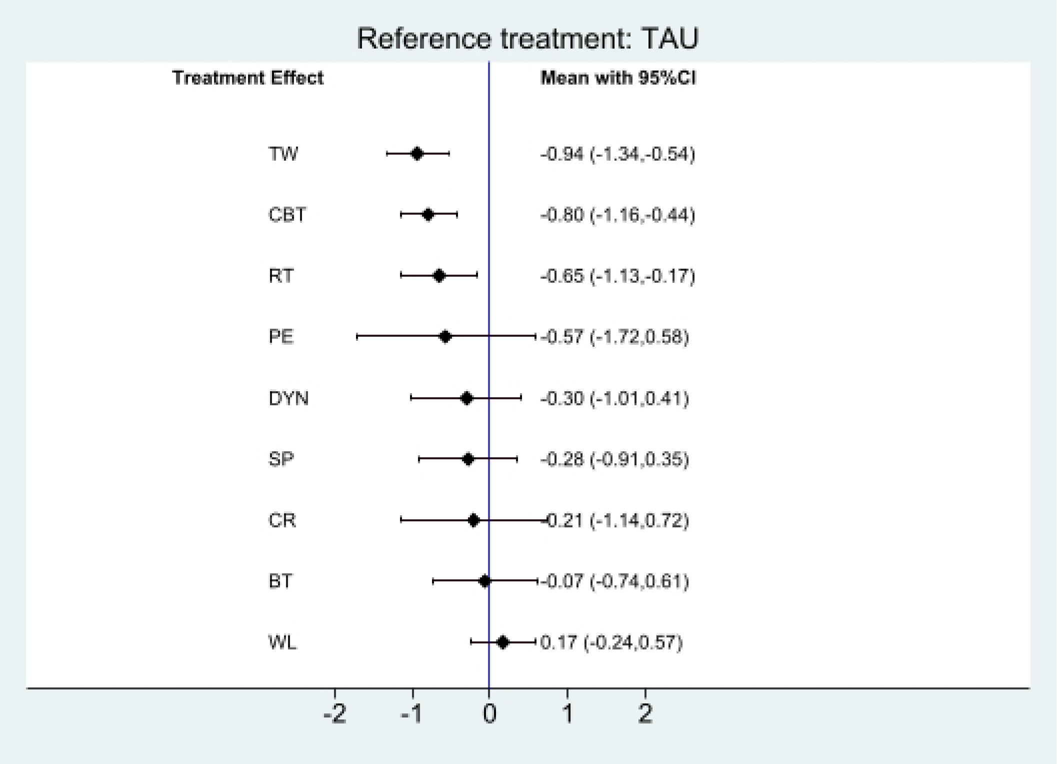<br>BT=behaviour therapy; CBT=cognitive-behaviour therapy; CR=cognitive restructuring; PE=psychoeducation; RT=relaxation therapy; SP=supportive therapy; DYN= psychodynamic therapy; 3W=third-wave CBT; TAU=treatment as usual; WL=waiting list.	SD=0.50; Restricted likelihood ratio test for heterogeneity: LRT = 110.37 (d.f. = 1) P = 0.000
		Overall incoherence
		chi2(8) = 14.09<br>Prob > chi2 = 0.94
		Loop-specific approach
		0/23 loops showed inconsistency
		Consistency between direct and indirect estimates
		AB = 0.041<br><br>AF = 0.044

© 2023 American Medical Association. All rights reserved.

Sensitivity 3

Excerpt from the protocol: “A series of three sensitivity analyses will be conducted to test whether the results could be influenced by the type of outcome hierarchy we have depicted in table 1. In these sensitivity analyses we will focus on each of three type of scales (Scales measuring generalized anxiety symptoms; Scales measuring anxiety symptoms; Scales measuring worry symptoms) separately”.

<table>
<tr><th>Analysis</th><th>Forest plot</th><th>Overall heterogeneity</th></tr>
<tr><td rowspan="7">Scales measuring GAD symptoms only</td><td rowspan="7">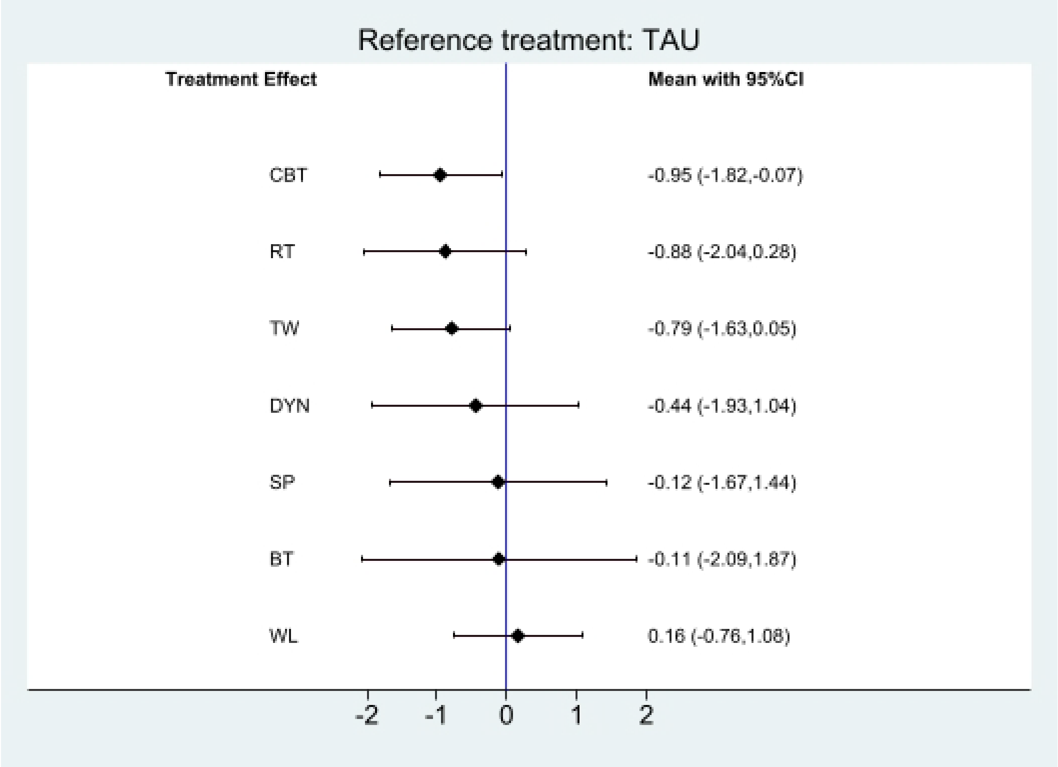

BT=behaviour therapy; CBT=cognitive-behaviour therapy; RT=relaxation therapy; SP=supportive therapy; DYN= psychodynamic therapy; 3W=third-wave CBT; TAU=treatment as usual; WL=waiting list.</td><td>SD=0.64; Restricted likelihood ratio test for heterogeneity: LRT = 69.76 (d.f. = 1) P = 0.000</td></tr>
<tr><td>Overall incoherence</td></tr>
<tr><td>chi2(8) = 13.22
Prob > chi2 = 0.02</td></tr>
<tr><td>Loop-specific approach</td></tr>
<tr><td>1/5 loops showed inconsistency</td></tr>
<tr><td>Consistency between direct and indirect estimates</td></tr>
<tr><td>AB = 0.032
AE = 0.031
BE = 0.004</td></tr>
</table>

© 2023 American Medical Association. All rights reserved.

Analysis	Forest plot	Overall heterogeneity
Scales measuring general anxiety only	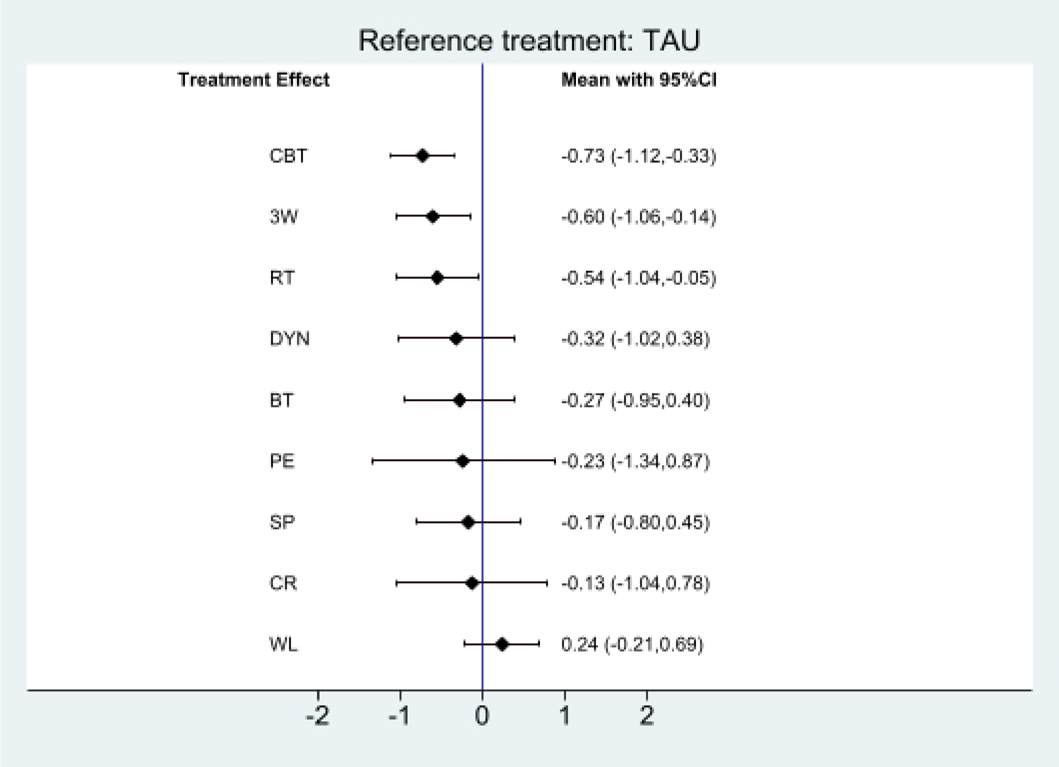<br>BT=behaviour therapy; CBT=cognitive-behaviour therapy; CR=cognitive restructuring; PE=psychoeducation; RT=relaxation therapy; SP=supportive therapy; DYN= psychodynamic therapy; 3W=third-wave CBT; TAU=treatment as usual; WL=waiting list.	SD=0.46; Restricted likelihood ratio test for heterogeneity: LRT = 55.84 (d.f. = 1) P = 0.000
		Overall incoherence
		chi2(8) = 11.35<br>Prob > chi2 = 0.97
		Loop-specific approach
		No loop (0/8 loops) showed inconsistency
		Consistency between direct and indirect estimates
		No inconsistencies

© 2023 American Medical Association. All rights reserved.

<table>
<tr><th>Analysis</th><th>Forest plot</th><th>Overall heterogeneity</th></tr>
<tr><td rowspan="7">Scales measuring worry symptoms only</td><td rowspan="7">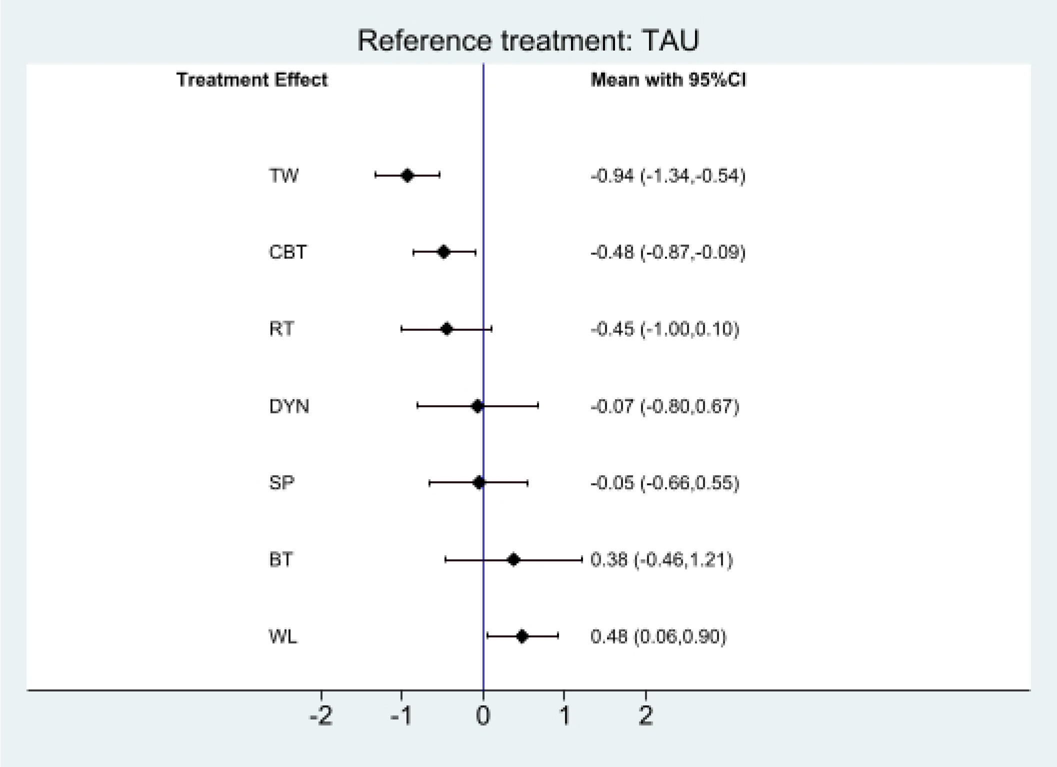

BT=behaviour therapy; CBT=cognitive-behaviour therapy; RT=relaxation therapy; SP=supportive therapy; DYN= psychodynamic therapy; 3W=third-wave CBT; TAU=treatment as usual; WL=waiting list.</td><td>SD=0.40; Restricted likelihood ratio test for heterogeneity: LRT = 46.14 (d.f. = 1) P = 0.000</td></tr>
<tr><td>Overall incoherence</td></tr>
<tr><td>chi2(8) = 8.66
Prob > chi2 = 0.95</td></tr>
<tr><td>Loop-specific approach</td></tr>
<tr><td>No loop (0/13 loops) showed inconsistency</td></tr>
<tr><td>Consistency between direct and indirect estimates</td></tr>
<tr><td>No inconsistencies</td></tr>
</table>

© 2023 American Medical Association. All rights reserved.

eAppendix N. Sensitivity analyses (post-hoc)

Excluding studies at high risk of bias

<table>
<tr><th>Analysis</th><th>Forest plot</th><th>Overall heterogeneity</th></tr>
<tr><td rowspan="7">Excluding studies at high risk of bias</td><td rowspan="7">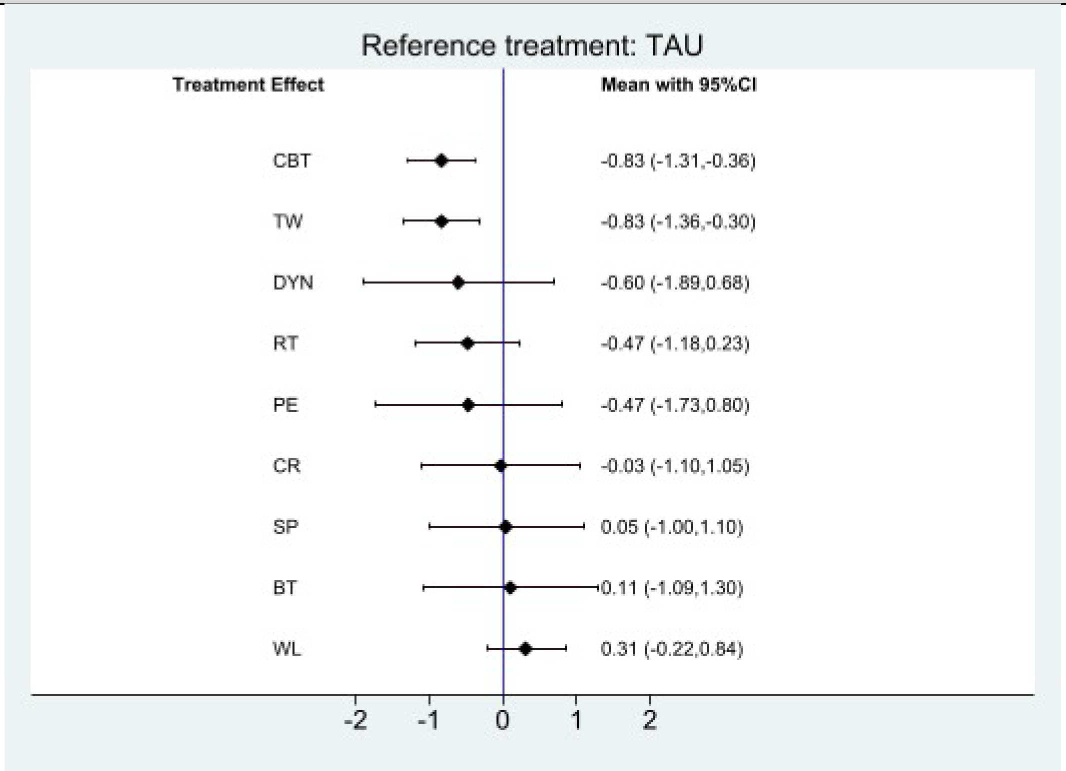

BT=behaviour therapy; CBT=cognitive-behaviour therapy; CR=cognitive restructuring; PE=psychoeducation; RT=relaxation therapy; SP=supportive therapy; DYN= psychodynamic therapy; 3W=third-wave CBT; TAU=treatment as usual; WL=waiting list.</td><td>SD=0.54; Restricted likelihood ratio test for heterogeneity: LRT = 95.64 (d.f. = 1) P = 0.000</td></tr>
<tr><td>Overall incoherence</td></tr>
<tr><td>chi2(8) = 8
Prob > chi2 = 0.89</td></tr>
<tr><td>Loop-specific approach</td></tr>
<tr><td>No loop (0/14 loops) showed inconsistency</td></tr>
<tr><td>Consistency between direct and indirect estimates</td></tr>
<tr><td>No inconsistencies</td></tr>
</table>

© 2023 American Medical Association. All rights reserved.

Considering behavioural experiments meant to test beliefs as part of the cognitive restructuring protocol

Analysis	Forest plot	Overall heterogeneity
Considering behavioural experiments meant to test beliefs as part of the cognitive restructuring protocol*	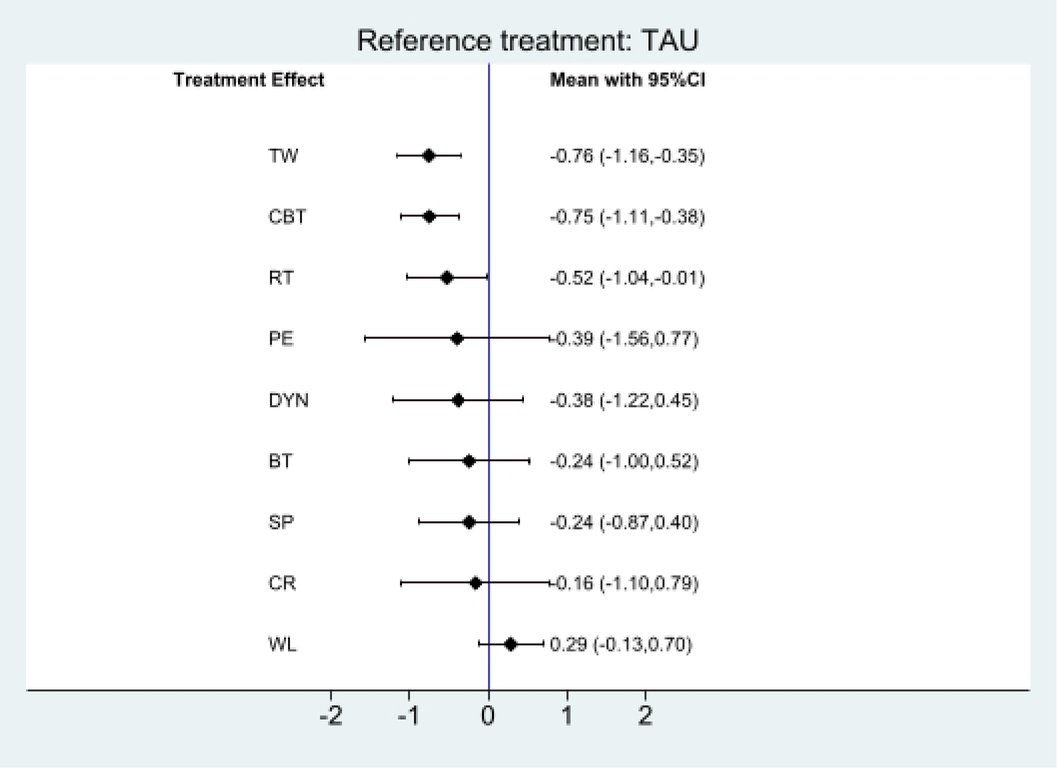<br>BT=behaviour therapy; CBT=cognitive-behaviour therapy; CR=cognitive restructuring; PE=psychoeducation; RT=relaxation therapy; SP=supportive therapy; DYN= psychodynamic therapy; 3W=third-wave CBT; TAU=treatment as usual; WL=waiting list.	SD=0.51; Restricted likelihood ratio test for heterogeneity: LRT = 101.26 (d.f. = 1) P = 0.000<br>**Overall incoherence**<br>chi2(8) = 14.02<br>Prob > chi2 = 0.90<br>**Loop-specific approach**<br>No loop (0/23 loops) showed inconsistency<br>**Consistency between direct and indirect estimates**<br>No inconsistencies

*Arntz 2003, Durham 1994,Durham 1987, Ost 2000

© 2023 American Medical Association. All rights reserved.

Excluding trials implementing a per-protocol analysis

Analysis	Network plot & Forest plot	Overall heterogeneity
Per protocol analysis RCT excluded	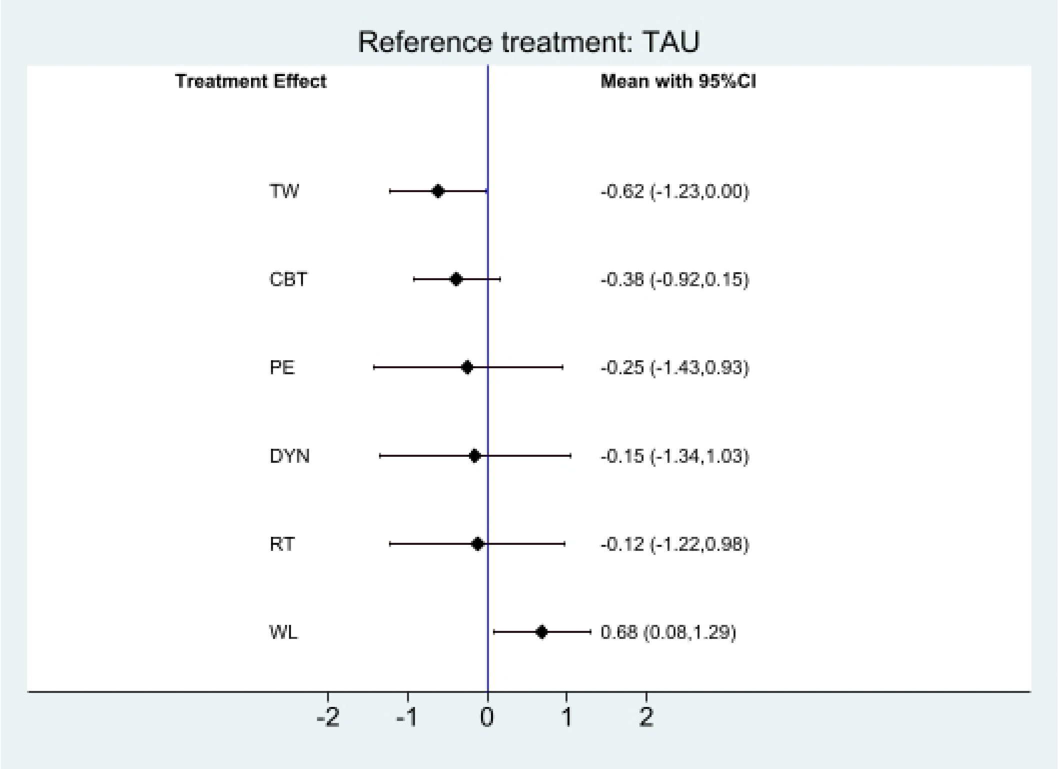	SD=0.47; Restricted likelihood ratio test for heterogeneity: LRT = 32.38 (d.f. = 1) P = 0.000<br>**Overall incoherence**<br>chi2(8) = 4.03<br>Prob > chi2 = 0.673<br>**Loop-specific approach**<br>0/3 loops showed inconsistency<br>**Consistency between direct and indirect estimates**<br>No inconsistencies

© 2023 American Medical Association. All rights reserved.

eAppendix O. Differences between protocol and review

The differences between this review and its registered protocol are:

1) We did not conduct the subgroup analysis on task-sharing because all the studies were conducted by mental health professionals (i.e., no intervention was task-shared).

2) We did not conduct the subgroup on “high-income countries versus studies conducted in low- and middle-income countries” because the great majority of RCTs (57 out of 66 – 86,4%) was conducted in HICs. Thus, the network for LMIC trials would have been informed by too small a number of studies to be statistically valid and provide clinically meaningful information.

© 2023 American Medical Association. All rights reserved.
