# Supplementary material for: Psychotherapies for Generalized Anxiety Disorder in Adults: A Systematic Review and Network Meta-Analysis of Randomized Clinical Trials
Source: JAMA Psychiatry. 2023 Oct 18;81(3):250–9. doi: 10.1001/jamapsychiatry.2023.3971 (PMC10585589; doi:10.1001/jamapsychiatry.2023.3971)
Supplement: Supplement 2. — Data Sharing Statement [file jamapsychiatry-e233971-s002.pdf]

Data Sharing Statement

Data
Data available: No

Additional Information
Explanation for why data not available: this study did not involve collection of primary data
